# Novel 2-(5-Arylthiophen-2-yl)-benzoazole Cyclometalated Iridium(III) dppz Complexes Exhibit Selective Phototoxicity in Cancer Cells by Lysosomal Damage and Oncosis

**DOI:** 10.1021/acs.jmedchem.3c01978

**Published:** 2023-12-23

**Authors:** Jana Kasparkova, Alba Hernández-García, Hana Kostrhunova, Marta Goicuría, Vojtěch Novohradsky, Delia Bautista, Lenka Markova, María Dolores Santana, Viktor Brabec, José Ruiz

**Affiliations:** † Czech Academy of Sciences, Institute of Biophysics, Kralovopolska 135, Brno CZ-61200, Czech Republic; ‡ Departamento de Química Inorgánica, Universidad de Murcia, and Biomedical Research Institute of Murcia (IMIB-Arrixaca), Murcia E-30100, Spain; § ACTI, Universidad de Murcia, Murcia E-30100, Spain

## Abstract

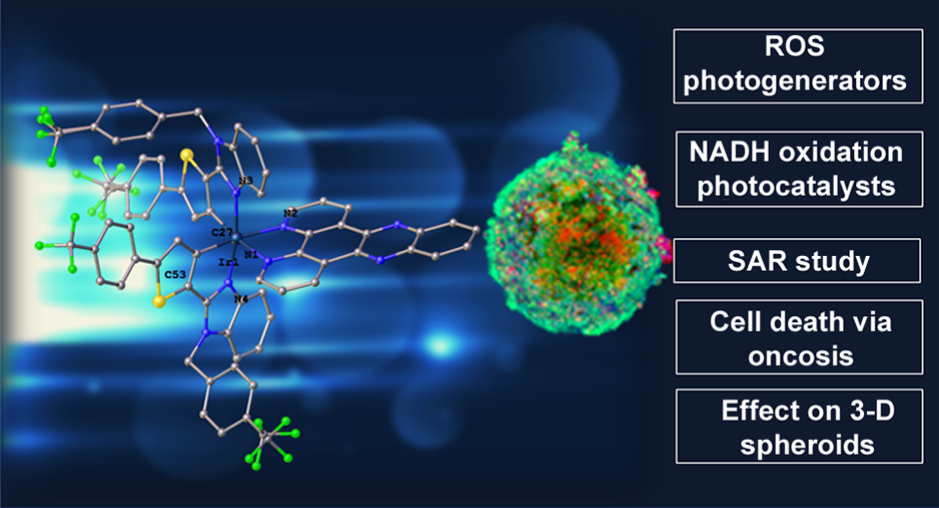

A second-generation series of biscyclometalated 2-(5-aryl-thienyl)-benzimidazole and -benzothiazole Ir(III) dppz complexes [Ir(C^N)_2_(dppz)]^+^, **Ir1**–**Ir4**, were rationally designed and synthesized, where the aryl group attached to the thienyl ring was *p*-CF_3_C_6_H_4_ or *p*-Me_2_NC_6_H_4_. These new Ir(III) complexes were assessed as photosensitizers to explore the structure–activity correlations for their potential use in biocompatible anticancer photodynamic therapy. When irradiated with blue light, the complexes exhibited high selective potency across several cancer cell lines predisposed to photodynamic therapy; the benzothiazole derivatives (**Ir1** and **Ir2**) were the best performers, **Ir2** being also activatable with green or red light. Notably, when irradiated, the complexes induced leakage of lysosomal content into the cytoplasm of HeLa cancer cells and induced oncosis-like cell death. The capability of the new Ir complexes to photoinduce cell death in 3D HeLa spheroids has also been demonstrated. The investigated Ir complexes can also catalytically photo-oxidate NADH and photogenerate ^1^O_2_ and/or ^•^OH in cell-free media.

## Introduction

Cancer is one of the most challenging diseases for modern medicine to tackle,^1^ and chemotherapy is the frontline of cancer treatment. Platinum-based chemotherapy drugs, such as cisplatin, oxaliplatin, and carboplatin, are used to treat many types of cancer, including lung, breast, ovarian, and testicular cancer. However, these drugs still exhibit serious problems, such as high general toxicity and drug resistance.^2,3^ The development of novel metal-based antitumor drugs that have high tumor selectivity and novel mechanisms of action is indeed a pressing need. Recently, a novel and central mode-of-action for the lead anticancer ruthenium compound BOLD-100, targeting several onco-metabolic pathways, has been identified.^4^

Photodynamic therapy (PDT) is an approved anticancer strategy that provides spatial and temporal control over drug activation and has attracted great attention in anticancer drug development to combat multidrug resistance, showing fewer side effects and higher selectivity than conventional therapies.^5−14^ Ruthenium complexes, with their rich photophysical and photochemical characteristics, have long been at the forefront of metal-based photosensitizers (PSs).^15−19^ The photodynamic therapy-based Ru(II) therapeutic, TLD-1433, prepared by McFarland and co-workers, has entered clinical trials and is currently in phase II for nonmuscle invasive cancer (NMIBC).^20^ Iridium complexes offer the advantages of long phosphorescence lifetime, significant photostability, and multiple photosensitization mechanisms.^21−26^

**Scheme 1:**
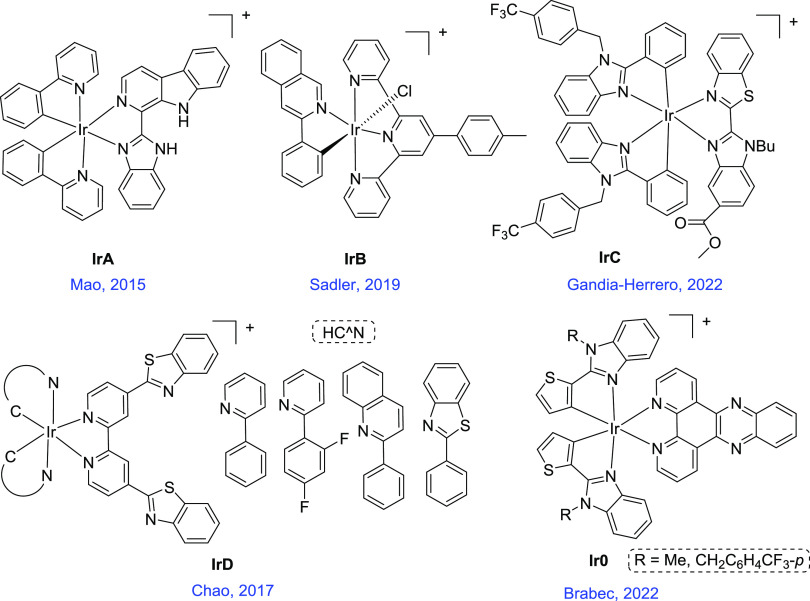
Chemical Structures of Representative Organoiridium(III) Complexes as Apoptosis Inducers through Photoinduced Lysosomal Damage (**IrA**),^27^ NADH Photooxidation Catalysts (**IrB**),^28^ Oncosis Inducers (IrC-IrD),^31,32^ and dppz Photosensitisers (**Ir0**) for PDT Closely Related to This Work^33,34^

Interestingly, Mao et al. prepared the Ir(III) complex **IrA** (Scheme 1), which can induce extensive cell apoptosis in cancer cells through photoinduced lysosomal damage.^27^ On the other hand, Ir(III) complex **IrB** (Scheme 1) has been shown to induce cancer cell death via the photooxidation of cellular coenzyme I, nicotinamide adenine dinucleotide (NADH) and reduction of cytochrome C_Fe(III)_,^28^ and recently, it has been suggested that the concept of in-cell photoredox catalysis has the potential to improve the efficiency of PDT significantly.^29^

Organometallic antitumor agents can also exhibit a variety of alternative modes-of-action to apoptosis, including translation inhibition, ferroptosis, oncosis, necroptosis, or paraptosis.^30^ Some examples of oncosis inducers, **IrC**-**IrD**, are also shown in Scheme 1.^31,32^

Previously, we reported some photoactive dipyridophenazine (dppz) biscyclometalated 2-thienyl-benzimidazole Ir(III) complexes **Ir0** (Scheme 1), able to induce efficient reactive oxygen species (ROS) photogeneration both under normoxic and hypoxic conditions using blue light irradiation,^33^ the methyl derivative being also a selective phototoxic agent toward cancer stem cells able to target mitochondria.^34^

**Scheme 2:**
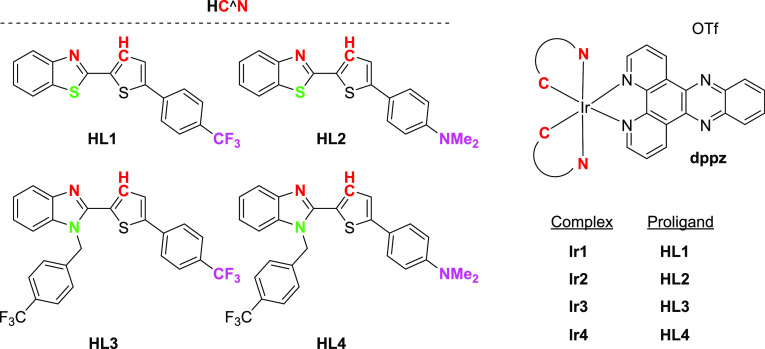
Structures of the New Ir(III) Tested Compounds

Herein, we rationally designed and synthesized a series of cationic Ir(III) photosensitizers **Ir1**–**Ir4** obtained by the cooperation of chromophoric ligand dppz, with four different cyclometalated ligands, 2-(5-arylthiophen-2-yl) benzothiazoles (**HL1** and **HL2**) and 2-(5-arylthiophen-2-yl)-1-(4-(trifluoromethyl)benzyl)-1*H*-benzo[*d*]imidazoles (**HL3** and **HL4**), where the aryl group attached to the thienyl ring is *p*-CF_3_C_6_H_4_ or *p*-Me_2_NC_6_H_4_, as shown in Scheme 2 to explore the structure–activity correlations for biocompatible anticancer photodynamic therapy. There are innumerable examples of benzimidazole-based compounds of pharmacological importance, and some of its organic derivatives are in clinical trials as potential anticancer drugs.^35^ The choice of the *p*-trifluoromethylbenzyl group on the nitrogen atom of the benzimidazole supports a higher lipophilic nature of the ligand. Some of the complexes were also designed to shift the absorption bands toward the more tissue-penetrating red region of the spectrum due to the chromophoric nature of 2-(5-arylthiophen-2-yl)benzothiazoles, such as **HL2** with an electron-donating *N*,*N*-dimethylaminophenyl ring connected to an electron-withdrawing benzothiazole. The new Ir(III) complexes are also assessed on their photophysical and photocatalytic properties, including their ability to photo-oxidate NADH, the evaluation for ^1^O_2_ and**/**or •OH photogeneration in cell-free media, as well as photosensitizers in 2D- and 3D- cancer models.

## Results and Discussion

### Synthesis and Characterization of Proligands (**HL1**–**HL4**) and Iridium(III) Complexes (**Ir1**–**Ir4**)

Four HC^N proligands **HL1**–**HL4** were prepared via Suzuki–Miyaura coupling starting from the corresponding intermediate bromoderivatives **A** and **B1** as depicted in Scheme 3 (see also Scheme S1 and the Experimental Section for details regarding the synthesis of intermediates **A** and **B**), **HL2** was previously reported as a nonlinear optical chromophore.^36^ The NMR spectra and positive ion HR ESI–MS of the intermediates and new proligands are shown in Figures S1–S14.

**Scheme 3:**
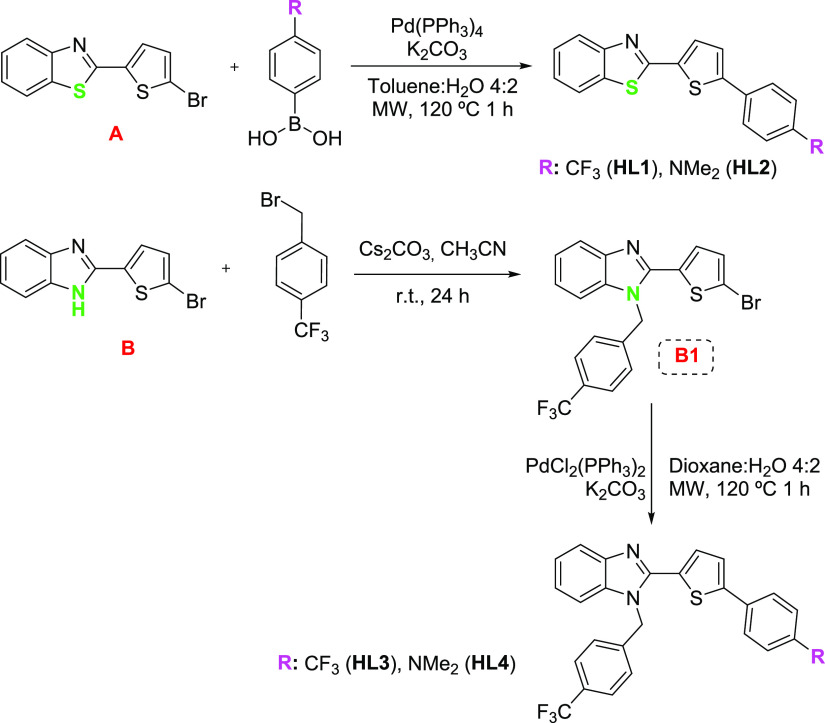
Synthetic Procedure for Intermediate **B1** and Proligands **HL1**–**HL4**

Preparation of complexes **Ir1**–**Ir4** as CF_3_SO_3_^•^ salts was achieved via two-step synthesis following reported standard literature procedures.^36^ The corresponding chloride-bridged dimeric iridium(III) complexes, [Ir(C^N)_2_(μ-Cl)]_2_, and the dppz ligand in a 1:2 molar ratio served as starting materials (Scheme S2). The obtained monomeric Ir(III) was fully characterized by ^1^H, ^1^H–^1^H COSY, and ^13^C{^1^H} and ^19^F{^1^H} NMR spectroscopy (Figures S15–S30). The ^1^H NMR spectra of all complexes show aromatic hydrogen peaks from 6 to 10 ppm, whereas the characteristic signal of the *p*-Me_2_NC_6_H_4_ group of the C^N ligands in complexes **Ir2** and **Ir4** appears around 3 ppm. The benzyl derivates **Ir3** and **Ir4** also show two signals around 6 ppm. The signals of the −CF_3_ moieties were also detected by ^19^F NMR spectra of the corresponding compounds. Final evidence of the correct formation of the compounds has been obtained from the high-resolution mass spectra with the identification of the molecular peaks corresponding to [Ir(C^N)_2_(dppz)] with the expected isotopic distribution (Figures S31–S34). The purities of complexes were checked by elemental analysis of C, H, N, and S. It was also confirmed that the purities of complexes were higher than 95% through RP-HPLC/MS in ACN/H_2_O (Table S1 and Figures S35 and S36).

### Crystal Structure by X-Ray Diffraction

**Figure 1:**
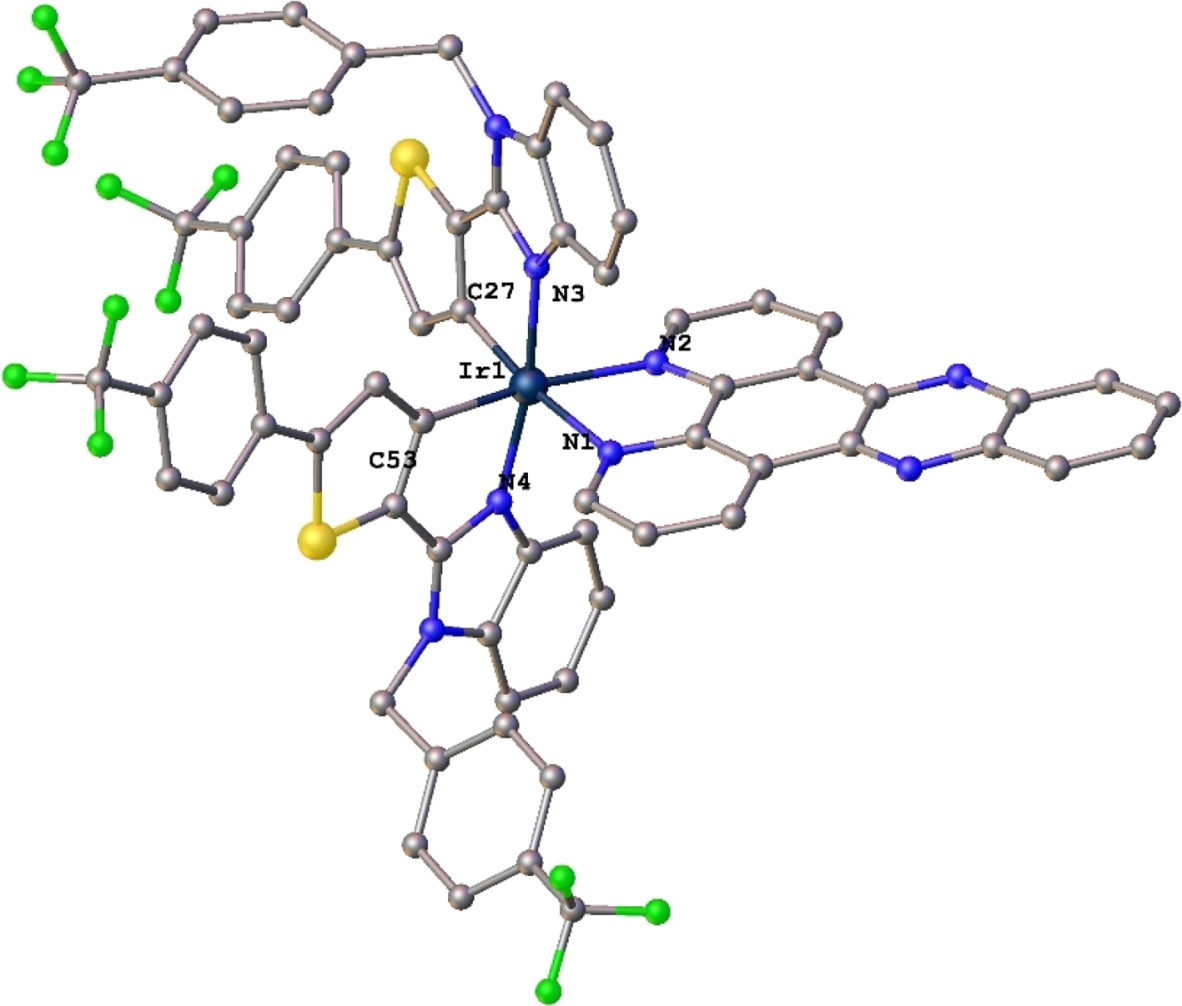
Molecular structure of **Ir3**. Hydrogen atoms, counterion, and solvent molecules are omitted for clarity. Selected bond lengths (Å) and angles (deg) for **Ir3**: Ir–C27:2.015(5), Ir–C53:1.997(5), Ir–N4:2.056(5), Ir–N1:2.131(4), Ir–N2:2.134(4), Ir–N3:2.060(4), C53–Ir–N4:95.2(2), C27–Ir–N3:79.8(2), and N1–Ir–N2:77.58(17). CCDC reference number is 2302438.

Suitable single crystals of **Ir3** for X-ray diffraction analysis were obtained by slow diffusion of hexane into a saturated dichloromethane solution in 3 days at room temperature. The crystal structure of **Ir3** is shown in Figure 1.

Crystallographic data are given in Table S2. The X-ray structure confirms the predicted geometry. The Ir atom is in a distorted octahedral coordination environment where the cyclometalated ligands present the two Ir–C and Ir–N bonds in a cis and trans arrangement, respectively, as previously observed. The distances around the Ir atom and C^N ligands are in the expected ranges for them, ∼2 Å, while the distances between Ir and N atoms of the ancillary ligand, dppz, are longer due to the *trans* influence of C^N ligands.^24,37^ Apart from the important cation–anion Coulomb interactions, the packing in the structure of **Ir3** is organized by intra- and intermolecular interactions C–H···X (X = F, O, N, and S, Table S3 and Figure S37), π–π interactions (Table S4 and Figure S38), and C–H···π interactions (Table S5 and Figure S39).

### Photophysical Characterization of the Compounds

As indicated above, **HL2** has been previously reported as a nonlinear optical chromophore,^36^ the greater electron-donating character of the dialkylamino group leading to a bathochromic shift in the absorption maxima, as the longest-wavelength transition is shifted from 360 nm for **HL1** to 405 nm for **HL2** (Figure S40 for UV/vis absorption spectra of **HL1**–**HL4** in acetonitrile).

**Figure 2:**
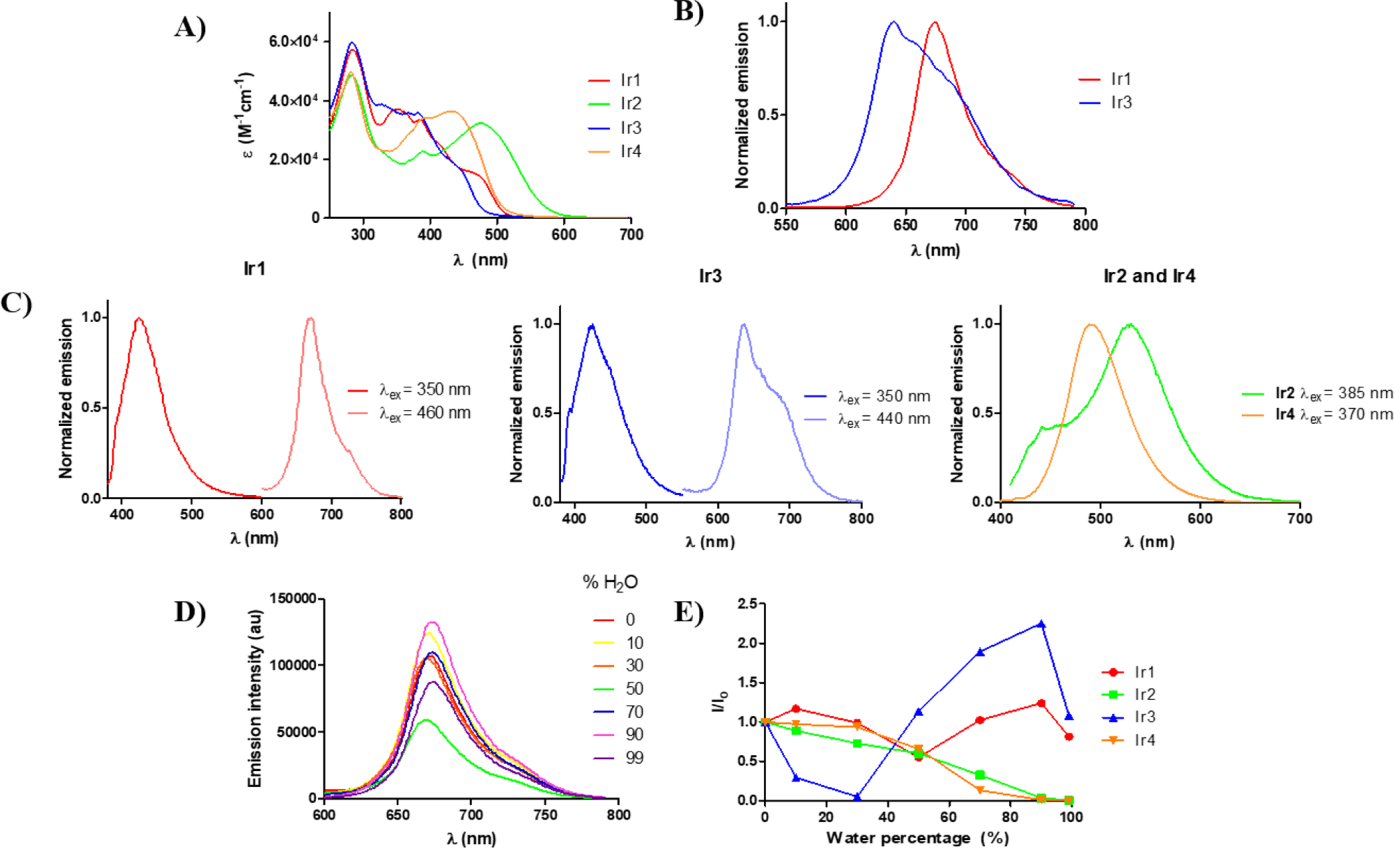
(A) UV/vis absorption spectra of **Ir1**–**Ir4** in water (1% DMSO). (B) Emission spectra of **Ir1** and **Ir3** complexes in water (λ_exc_ = 405 nm, 10 μM). (C) Emission spectra of iridium complexes in aerated acetonitrile. (D) Emission spectra of **Ir1** (λ_exc_ = 405 nm, 10 μM) in DMSO/water mixtures with different *f*_w_. (E) Plots of *I*/*I*_0_ versus fw for **Ir1**–**Ir4**. *I*_0_ represents the emission intensity of a pure DMSO solution.

The UV/vis absorption spectra of complexes **Ir1**–**Ir4** were recorded in water (1% dimethyl sulfoxide (DMSO), Figure 2A and Table S6) and acetonitrile (Figure S41 and Table S6). As observed, all UV/vis absorption spectra of the cyclometalated iridium(III) complexes show intense absorption bands below 350 nm, which could be attributed to spin-allowed ligand centered π–π* transitions located on the C^N and dppz ligands (Figure 2A). At longer wavelengths (λ >350 nm), the less intense absorption bands could be assigned to spin-allowed metal-to-ligand (^1^MLCT), ligand-to-ligand charge transfer (LLCT) transitions, or ligand spin forbidden singlet-to triplet (^3^MLCT) nature, as a consequence of the spin–orbit coupling of an Ir(III) heavy atom (ζ = 3909 cm^–1^),^38^ which allows for fast and efficient intersystem crossing (ISC) to convert singlet excitons to triplets.^39,40^ The triplet nature of these complexes, supported on the long lifetime determined experimentally for the emissive states (*vide infra*) and also on the high Stokes shifts, could make them appropriate for bioimaging and PDT.^41^ In addition to the above characteristics, we could observe that the new complexes presented tails in their absorption spectra until 520 nm or even until 620 nm (in the case of **Ir2**), which is desirable for PDT.

All the new complexes **Ir1**–**Ir4** were emissive in aerated acetonitrile, as shown in Figure 2C, **Ir1** and **Ir3** being dual emitters. In deaerated acetonitrile, the absolute emission quantum yields of complexes **Ir1** and **Ir3** were 0.015 and 0.013, respectively (Table 1), while for **Ir2** and **Ir4** were lower than 0.01. The emission lifetimes in deaerated acetonitrile for **Ir1** and **Ir3** were about 1 μs. The emission properties of **Ir1** and **Ir3** were also studied in water (λ_exc_ = 405 nm, 10 μM, Figure 2B), exhibiting red and orange phosphorescent emissions, respectively, whereas **Ir2** and **Ir4** were nonluminescent in this solvent, maybe due to their aggregation (*vide infra* and Figure 2E).

**Table 1:** Excitation (λ_exc_) and Emission (λ_em_) Wavelengths of Ir Complexes in Aerated Acetonitrile^b^

complex	solvent	**λ**_**exc**_^a^(nm)	**λ**_**em**_ (nm)	**τ**_**em**_^b^ (**μs**)	**ϕ**_**em**_^b^ (%)
**Ir1**	CH_3_CN	350	426	1.02	0.015
		460	672		
**Ir2**	CH_3_CN	385	531		<0.01
**Ir3**	CH_3_CN	350	425	1.01	0.013
		440	636		
**Ir4**	CH_3_CN	370	489		<0.01

a λ_exc_ maxima.

b Emission lifetimes (τ_em_, λ_NanoLED_ = 372 nm) and absolute emission quantum yields (ϕ_em_) of complexes in dearated acetonitrile.

The aggregation-induced emission (AIE) and aggregation-caused quenching (ACQ) effects of the new PSs were next evaluated in DMSO/water mixtures with varied water volumetric fractions (*f*_w_). As shown in Figure 2D,E and Figure S42, **Ir2** and **Ir4** complexes, containing the *p*-Me_2_NC_6_H_4_ group on the thienyl ring, show classic ACQ properties. In contrast, **Ir1** and **Ir3**, containing the *p*-CF_3_C_6_H_4_ substituent, exhibit typical AIE optical characteristics,^42^ reaching the latest maximum emission intensity at 90% water, making both of them good candidates for bioimaging purposes (*vide infra*).

### Stability and Photostability Studies

**Figure 3:**
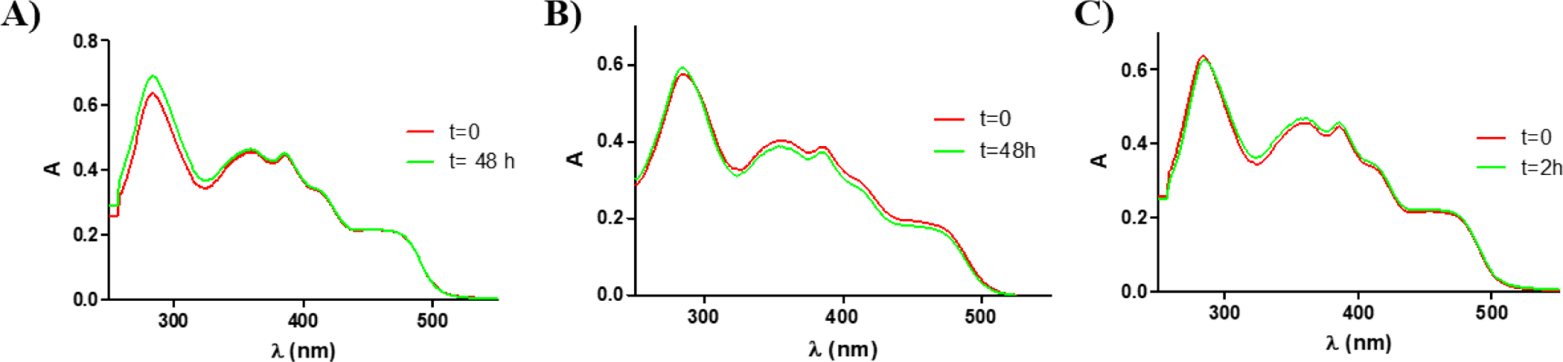
UV/vis absorption spectra of complex **Ir1** (10 μM) (A) in DMSO for 48 h, (B) after incubation at 37 °C for 48 h in RPMI (5% DMSO), and (C) upon blue light irradiation (4 mW cm^–2^) for 2 h in DMSO.

The dark and light stabilities are essential for photosensitizers. The stabilities of complexes **Ir1**–**Ir4** under the dark were studied in DMSO and the Roswell Park Memorial Institute (RPMI) cell culture medium (5% DMSO) at 37 °C using UV/vis spectroscopy (Figure 3A,B for **Ir1** and Figures S43 and S44 for **Ir2**–**Ir4**). As shown, the spectra were unchanged in these conditions at least for 48 h, suggesting that the investigated complexes are stable in both DMSO and cell culture media. Furthermore, the dark stabilities of complexes **Ir1** and **Ir3** were also studied in biological relevant conditions by HPLC-MS, i.e., dissolved in RPMI (1% DMSO), finding that they were completely stable after 24 h incubation at 37 °C (Figures S45 and S46). On the other hand, the photostabilities in DMSO for the new complexes were tested under blue light irradiation (λ = 465 nm, 4 W m^–2^). As shown in Figure 3C (for **Ir1**) and Figure S47 (for **Ir2**–**Ir4**), their absorption spectra remained unaltered after light exposure for 2 h. In addition, the photostabilities of **Ir1**–**Ir4** in DMSO-*d*_6_ (1 mM) were also tracked by ^1^H NMR (Figures S48–S51). The results showed that their ^1^H NMR spectra remained unchanged after 6 h under blue light irradiation (λ = 465 nm, 4 mW/cm^2^) at 25 °C.

### Photooxidation of NADH and Evaluation for ^1^O_2_ and/or ^**•**^OH Photogeneration in Cell-Free Media

**Figure 4:**
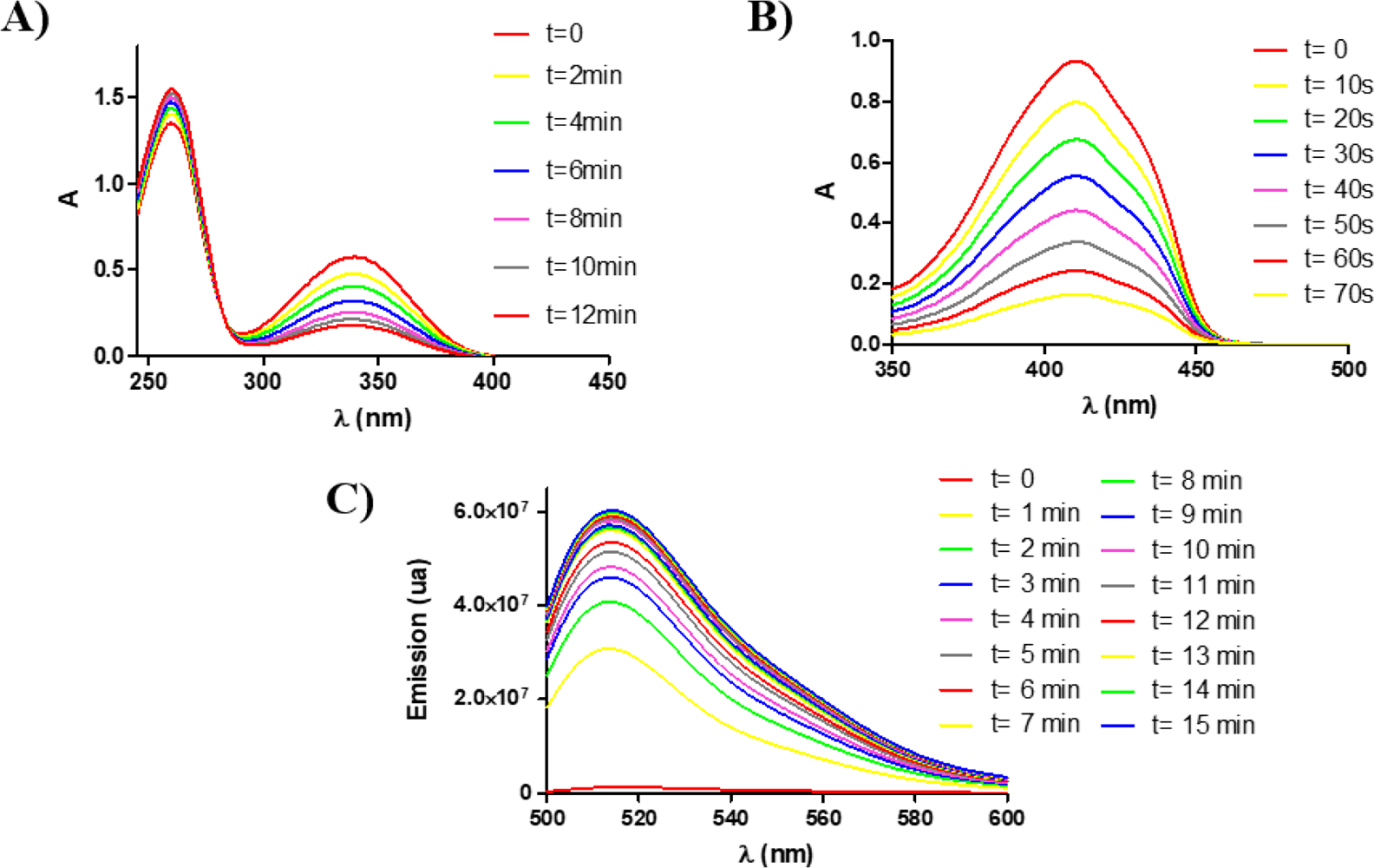
(A) Decreasing of NADH absorption spectra (100 μM) in the presence of **Ir1** (1 μM) in PBS (5% DMF) under blue light (4.2 mW cm^–2^). (B) Absorbance decrease of 1,3-diphenylbenzofuran (DPBF) (50 μM) in the presence of **Ir1** in acetonitrile when irradiated with blue light (0.5 mW cm^–2^). (C) Increase of the fluorescence spectra emission of 3′-*p*-(hydroxyphenyl)fluorescein (HPF) upon photoirradiation of **Ir1** with blue light (4.2 mW cm^–2^) in PBS (5% DMF). HPF fluorescence was excited at 490 nm.

NADH is an important coenzyme, which participates in the maintenance of intracellular redox balance.^28^ To evaluate the capacity of the complexes to induce photocatalytic oxidization of the coenzyme in aerated solutions, **Ir1**–**Ir4** complexes (1 μM) were incubated in the presence of NADH (100 μM) in PBS (5% dimethylformamide (DMF)). As shown in Figure S52, UV/vis spectra of NADH remained unchanged in the presence of the complexes in dark conditions and after light irradiation without using any complex. However, the absorbance of NADH decreased gradually with all complexes in a very low concentration after light irradiation (Figure 4A for **Ir1** and Figure S53 for **Ir2**–**Ir4**).

By measuring the changes at λ = 339 nm (absorption peak of NADH), turnover number (TON) and turnover frequency (TOF) values were calculated, obtaining surprising values for all complexes. The introduction of the *p*-Me_2_NC_6_H_4_ substituent on the thienyl ring improves the ability to oxidize NADH after irradiation with light, compared to trifluoromethyl group derivatives. **Ir4** was the most active and interesting compound with a TOF (h^–1^) value of 403, whereas **Ir3** was the less active compound with a TOF (h^–1^) of 241 (see the Supporting Information for further details; Figure S54 and Table S7). **Ir2** and **Ir4**, containing the *p*-Me_2_NC_6_H_4_ group on the thienyl ring and more intense bands around 520 nm, also show high TOF (h^–1^) values when irradiating with green light (71 and 39, respectively).

Next, we investigated which type of ROS iridium compounds produce in cell-free media. First, the ability of synthesized Ir(III) complexes to produce ^1^O_2_ was evaluated spectroscopically by the decreasing of 1,3-diphenylbenzofuran (DPBF) absorbance at 411 nm (Figure 4B and Figures S55 and S56) upon irradiation with blue light (465 nm, 0.5 mW cm^–2^). **Ir1** and **Ir3**, which contain the *p*-CF_3_C_6_H_4_ group on the thienyl ring, showed a medium-high singlet oxygen quantum yield (∼65%), whereas **Ir2** and **Ir4**, which contain *p*-Me_2_NC_6_H_4_ group, exhibit a less singlet oxygen quantum yield (∼10%).

We also investigated the ability of the new compounds to produce hydroxyl radicals, a specific type-I ROS, in PBS (5% DMF) by using a spectroscopic method based on the oxidation of the nonfluorescent HPF probe by OH· to the corresponding fluorescent product.^43,44^ As shown in Figure 4C and Figure S57, under blue light irradiation, all the newly synthesized compounds increased the fluorescence intensity of HPF, which indicates the generation of a hydroxyl radical. We could observe that Ir(III) complexes **Ir1** and **Ir3** containing the *p*-CF_3_C_6_H_4_ substituent on the thienyl ring reached the highest maximum emission intensity after 15 min of irradiation compared with their analogs containing the NMe_2_ group.

### Antiproliferative and Phototoxic Effect of Iridium Complexes

The photoactivities of complexes **Ir1**–**Ir4** were determined against human cervix adenocarcinoma (HeLa) cells, human skin melanoma cells A375, and human colon adenocarcinoma HCT116 cells. Cervical, skin, and colon tumors are predisposed to photodynamic therapy due to their accessibility to irradiation; therefore, cell lines derived from these tissues have been selected for this study.

The cells were treated with tested compounds diluted in Earle’s balanced salt solution (EBSS) for 1 h in the dark to allow the complexes to penetrate the cells. Afterward, the cells were irradiated for 1 h with blue light (LZC-4 photoreactor equipped with 16 lamps LZC-420, λ_max_ = 420 nm) or sham irradiated. EBSS containing an Ir complex was then removed, and cells were incubated in the complete, drug-free Dulbecco’s modified Eagle’s medium (DMEM).

The metabolic activity of the cells (proportional to number of viable cells) was determined 72 h after irradiation using the standard 3-[4,5-dimethylthiazol-2-yl]-2,5 diphenyl tetrazolium bromide (MTT) assay. The IC_50_ values (defined as concentration of the agent inhibiting cell growth by 50%) were calculated from curves constructed by plotting relative absorbance (related to that found for untreated, irradiated, or sham irradiated cells) versus drug concentration. It has also been confirmed that the irradiation under the conditions used throughout our study had a negligible effect on the viability of untreated control cells. For comparative purposes (and at the reviewer’s request), the clinically used metallodrug cisplatin was included in the experiment.

As indicated in Table 2, all investigated complexes show a significant phototoxic effect on cervical, melanoma, and colon carcinoma cells with IC_50_ values in the submicromolar (**Ir1** and **Ir2**) or low micromolar (**Ir3** and **Ir4**) range. Importantly, without irradiation, they did not show any evident effect on cellular viability and proliferation of HeLa and HCT116 cells, even at 50 μM concentrations; the higher concentrations could not be tested due to the limited solubility of Ir complexes in media. Melanoma A375 cells were slightly more sensitive, particularly to the complexes **Ir1** and **Ir2** (Table 2). Nevertheless, phototoxicity indexes for A375 cells are eminent, 239 and 117 for **Ir1** and **Ir2**, respectively.

**Table 2:** IC_50_ Values (μM) Obtained for Cells Treated with the Ir Complexes and Irradiated by Blue Light (1 h, λ_max_ = 420 nm, 58 ± 2 W m^–2^) or Sham Irradiated as Determined by the MTT Assay^a^

	HeLa		HCT116		A375		hTERT EP156T	MRC5
complex	irrad	sham irrad	irrad	sham irrad	irrad	sham irrad	48 h dark	48 h dark
**Ir1**	0.31 ± 0.02	>50	0.30 ± 0.03	>50	0.18 ± 0.05	43 ± 5	39 ± 2	>50
**Ir2**	0.7 ± 0.1	>50	0.77 ± 0.07	>50	0.4 ± 0.2	47 ± 4	47 ± 3	>50
**Ir3**	1.22 ± 0.09	>50	1.6 ± 0.4	>50	0.9 ± 0.1	>50	>50	>50
**Ir4**	3.7 ± 0.8	>50	3.4 ± 0.9	>50	2.6 ± 0.4	>50	>50	>50
**cisPt**	27 ± 3	23 ± 4	27 ± 7	19 ± 4	15 ± 2	14 ± 1	ND	6.0 ± 0.7

a The data are expressed as mean values ± SD, *n* ≥6. ND = not determined. **cisPt** = cisplatin.

Several Ir complexes have previously been shown to affect mitochondrial metabolism.^45−47^ As the MTT assay is based on mitochondrial metabolization, the results may be affected by the possible impact of tested compounds on mitochondria. Therefore, the above-described phototoxicity experiments have also been performed using a Sulforhodamine B (SRB) assay based on measuring cellular protein content, i.e., the mechanism other than mitochondrial metabolism. As shown in Table S8, the SRB assay confirmed the same trend in the biological activity of all tested complexes after irradiation (as well as their dark inactivity) as found by MTT, with IC_50_ values in good agreement for both MTT and SRB assays. Thus, the data indicate that, regardless of whether the complexes target mitochondria, mitochondrial dehydrogenases are not affected by Ir complexes tested in this work.

The low toxicity of nonirradiated Ir complexes toward human noncancerous cells was confirmed by the fact that their effect was very low or even undetectable during long-term exposure when the human primary prostate epithelial hTERT EP156T and human lung fibroblast MRC5 cells were exposed to the complexes continuously for 48 h (Table 2).

Absorption spectra (Figure 2A) of the complexes reveal that the **Ir2** shows slight but significant absorbance even at wavelengths longer than those corresponding to the blue light. Therefore, the photoactivation of **Ir2** was tested also using a green (λ_max_ = 545 nm) or red (λ_max_ = 613 nm) light irradiation. For this experiment, samples were irradiated with a visible cool white lamp (LZC-Vis, Luzchem), and the appropriate green or red filter was applied; spectral characteristics can be seen in Figure S58.

As indicated (Table 3), **Ir2** was photoactivatable if irradiated by green or red light. In concord with the lower absorption of the Ir complexes at these wavelengths, the activity was weaker than when using the blue light. Nevertheless, the IC_50_ values range over low micromolar concentrations, confirming the possibility of utilizing longer wavelengths to activate this complex.

**Table 3:** IC_50_ Values (μM) Obtained by the MTT Assay for HeLa Cells Treated with **Ir2** and Irradiated by Blue, Green, and Red Light^a^

irradiation	IC_50_ (μM)
blue light (λ_max_ 420 nm, 58 ± 2 Wm^−2^)	0.7 ± 0.1
green light (λ_max_ 545 nm, 23 ± 1 Wm^−2^)	4.3 ± 0.8
red light (λ_max_ 613 nm, 20 ± 1 Wm^−2^)	8.5 ± 0.9

a The data are expressed as mean values ± SD, *n* ≥4.

Further experiments were aimed at a deeper description of the mechanism underlying the photoactivity of the Ir complexes. For these experiments, HeLa cells were used to compare already published data obtained with a previous series of Ir complexes of similar structure.^33^

### Intracellular Accumulation

The ability to penetrate cells and intracellular accumulation is an essential prerequisite for the biological effect of low molecular mass drugs. Therefore, to evaluate the cellular uptake and accumulation of individual Ir complexes, the intracellular content of Ir in HeLa cells was determined by inductively coupled plasma mass spectrometry (ICP-MS) after the cells were treated for 2 h with tested compounds at their equimolar (3 μM) concentrations. Generally, the cellular uptake of Ir complexes was in the following order: **Ir1** ≈ **Ir2** > **Ir3** > **Ir4** (Table 4), which roughly corresponds to their photoefficacy (Table 2).

**Table 4:** Accumulation of Ir Complexes in Hela Cells^b^

compound	ng Ir/10^6^ cells^a^
**Ir1**	138 ± 13
**Ir2**	127 ± 6
**Ir3**	100 ± 3
**Ir4**	30 ± 5

a The data are expressed as mean values ± SD, *n* = 3.

b Cells were treated with the investigated Ir compounds (3 μM, 2 h, dark, 37°C).

Interestingly, preincubation of the cells with inhibitors of endocytosis chloroquine and methyl-beta-cyclodextrin led to a significant decrease in the amount of Ir accumulated in the cells (Table S9), confirming endocytic pathways as a mechanism significantly participating in the uptake of the Ir complexes.

As indicated above, a correlation between photoactivity and the accumulation of the Ir(III) complexes in cancer cells was observed. As shown, when comparing the two benzothiazole Ir(III) derivatives (**Ir1** and **Ir2**), both the accumulation of **Ir1** (a compound containing the *p*-CF_3_C_6_H_4_ group on the thienyl ring) and its photoactivity in the three cancer cell lines are higher than those of **Ir2** (Table 2). Similar observations were found when comparing the two benzimidazole derivatives (**Ir3** and **Ir4**). On the other hand, the photoactivation of the benzothiazole compounds (**Ir1** and **Ir2**) in cancer cells was higher than that of the benzimidazole Ir complexes (**Ir3** and **Ir4**). Important to note, the best performer, **Ir1**, is also the best intracellular ROS generator of the series, after irradiation with blue light (*vide infra*).

### Intracellular ROS Production

**Figure 5:**
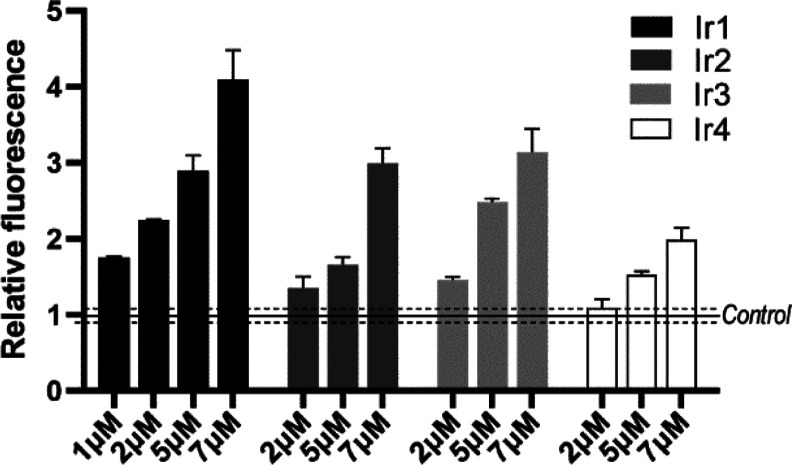
Generation of ROS in HeLa cells pretreated with **Ir1**–**Ir4** and irradiated, as detected by the CellROX flow cytometry assay. Data were normalized to those obtained for control (untreated) irradiated cells and represented mean ± SEM of two independent measurements.

Several Ir(III) complexes, including those structurally similar to Ir(III) complexes tested here, have been shown to induce ROS production; the phototoxicities of these complexes were attributed to their ability to arouse ROS.^33,34^ Therefore, the CellROX assay was employed to assess intracellular levels of ROS in HeLa cells treated with Ir complexes **1**–**4**. In this assay, the fluorescence intensity at 660 nm was determined to measure ROS concentration. After irradiation, the intracellular ROS level was significantly elevated for cells treated with all tested complexes (Figure 5), with **Ir1** and **Ir4** being the most and least effective, respectively. The results of this experiment correlate with the data on phototoxicity (Table 2), suggesting that the photoactivity of the tested Ir complexes likely results from the intracellular ROS generation along with the apparent ability of the complexes to accumulate in tumor cells (Table 4).

### Mechanism of Cell Death

**Figure 6:**
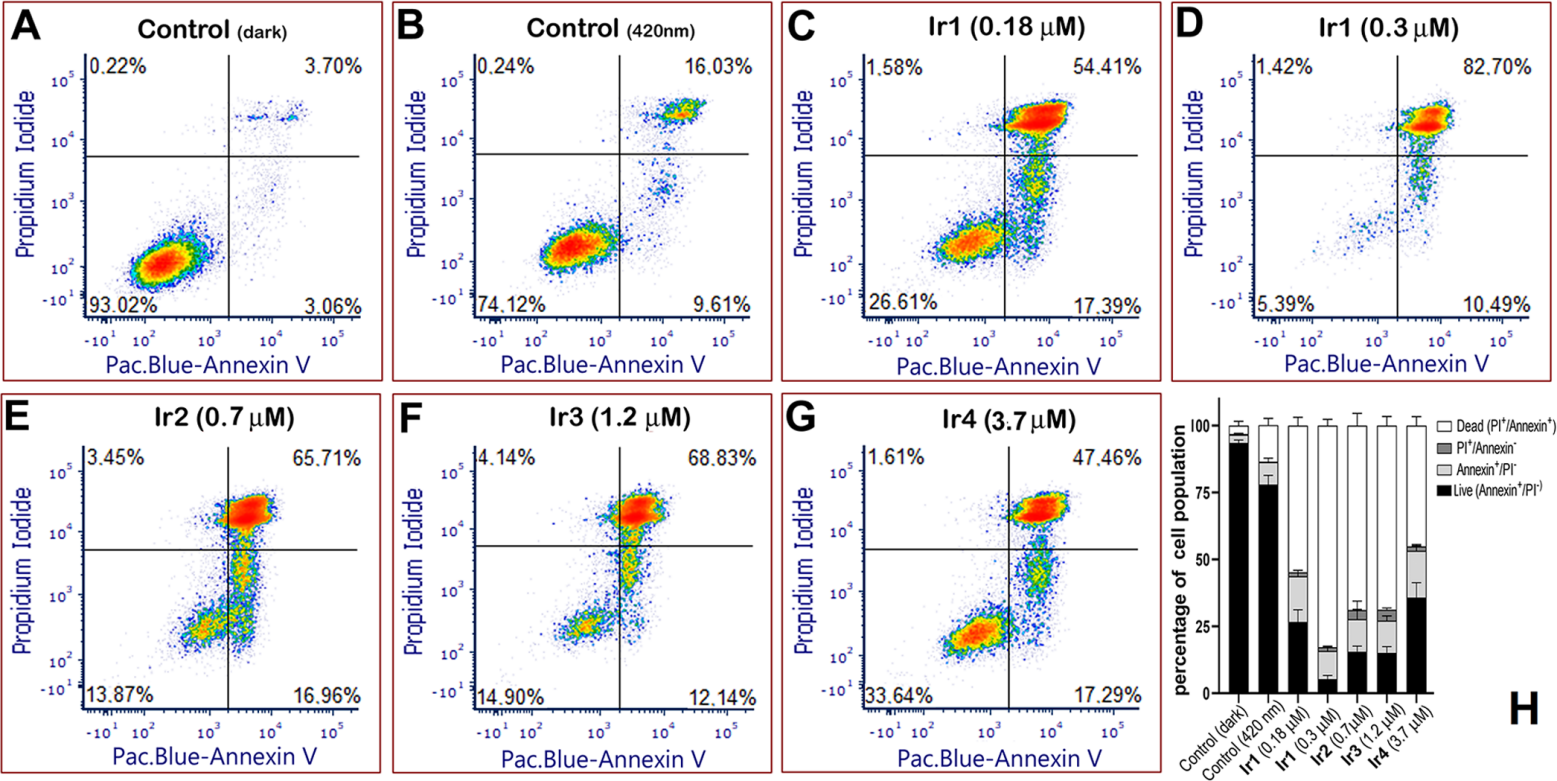
Detection of cell death mode by staining with Annexin V Pacific blue and PI using flow cytometry. HeLa cells were incubated for 1 h with the equitoxic concentration of Ir complexes (IC_50,72h_), irradiated for 1 h, and then recovered for 24 h in compound-free media. Control untreated cells (nonirradiated and irradiated) were also included in the experiment. The percentages of cell population in respective quadrants are indicated. Panels A–G: representative 2D density plots. Panel H: quantitative evaluation of the experiment. Columns represent a mean ± SD from three independent experiments, 30,000 cells were analyzed in each sample.

Next, cell death mode was studied by the annexin V propidium iodide (PI) dual staining assay 24 h after the cells were irradiated to unravel the cellular response to the tested Ir complexes. Figure 6 shows that treatment of Hela cells with Ir complexes **1**–**4** followed by irradiation induced a noticeable increase in the annexin V positive/PI-negative cell population (right bottom quadrant in Figure 6) compared to the control, untreated cells. Moreover, the population of the cells in the late stages of death (both annexin V and PI positive cells, right upper quadrant) was also markedly enlarged. It suggests that, after being irradiated, the Ir complexes effectively caused cell death. Interestingly, **Ir1** was much more effective in killing cells than the other three complexes, producing ca. 83% of the cell population already dead, although the concentrations of the Ir complexes used in this experiment were equitoxic [IC_50,72h_ (Table 2), i.e., 0.3, 0.7, 1.2, and 3.7 μM for **Ir1**, **Ir2**, **Ir3**, and **Ir4**, respectively]. To achieve the effectivity of **Ir1** similar to that of Ir complexes **2** and **3**, **Ir1** had to be used at a considerably lower concentration (0.18 μM) (Figure 6C). Thus, the results of this experiment (Figure 6) revealed a difference in the efficiency of the investigated Ir complexes **1**–**4** to induce death in cancer cells, with **Ir1** acting much faster than Ir complexes **2**–**4**, so that the effect of **Ir1** after 24 h is significantly higher, while after 72 h, the effects are roughly equal (equitoxic concentrations corresponding to IC_50_,_72h_ were used).

The use of fluorescently labeled annexin V in this assay is designed to detect apoptosis by targeting the loss of phospholipid asymmetry of the plasma membrane. Apoptotic cell death is accompanied by a change in the plasma membrane structure by surface exposure to phosphatidylserine (PS), while the membrane integrity remains intact. Externalization of PS is detected by its affinity for annexin V.^48^ Therefore, the PI-negative/annexin V positive cell population is commonly considered demonstrably apoptotic. However, examples of PS exposure prior to membrane compromise have also been observed in oncotic cells, so this may not necessarily be a feature unique to apoptosis.^49,50^ Therefore, further experiments were aimed to distinguish between apoptotic and oncotic modes of cell death.

### Morphology of the Cell and Caspase-3 Activation

As apoptosis and oncosis share several features (translocation of PS to the outer surface, DNA laddering, etc.),^50^ morphological alterations induced in cells treated with the investigated compounds provide the major unequivocal evidence of cell death mode.^51^ Prelethal changes typical for oncosis are characterized by cell swelling and karyolysis, clearing of the cytosol, nuclear chromatin clumping, formation of cytoplasmic bulges or blisters that are organelle-free, and increased membrane permeability.^52,53^ In contrast to oncosis, classic apoptosis is caspase-3 dependent and is accompanied by cell shrinkage and the formation of apoptotic bodies and budding.^54^

**Figure 7:**
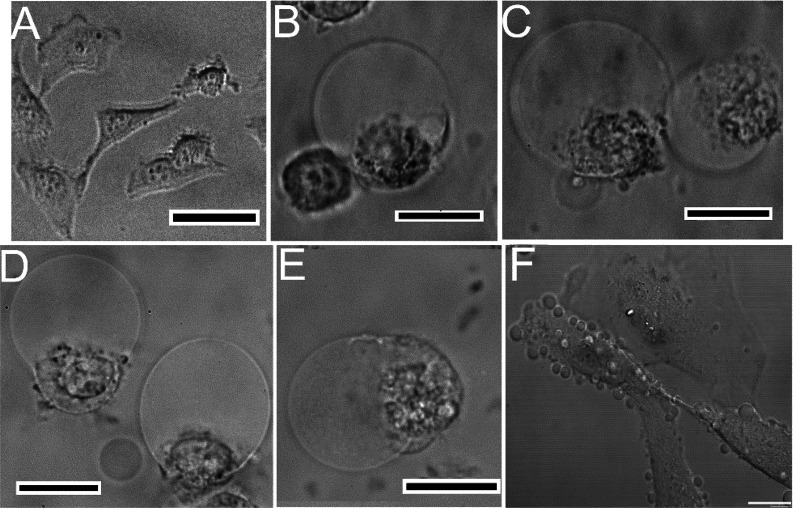
Microscopic images of HeLa cell morphology. (A–E) Vacuolization of cytoplasm and cell swelling, as revealed by inverted optical microscope. Cells were treated with Ir**1** (0.3 μM, panel B), **Ir2** (0.7 μM, panel C), **Ir3** (1.2 μM, panel D), **Ir4** (3.7 μM, panel E), or untreated (panel A) for 1 h, irradiated, and incubated in compound-free media for 2 h. Scale bars represent 50 μm. (F) Confocal image of plasma membrane blebbing. Hela cells were incubated with **Ir1** (0.3 μM), and the image was taken 15 min after irradiation. Scale bar in confocal image: 15 μm.

Figure 7 and Figure S59 show the morphological alteration observed 2 h after the HeLa cells were treated with Ir complexes **1**–**4** at their equitoxic concentrations (IC_50,72h_) and irradiated. The most striking feature was the cytoplasm vacuolization when the vacuoles filled up almost the entire cytoplasm and showed the absence of organelles. The whole cells were swollen and rounded (Figure 7B–E). A significant cytoplasm blebbing was evident in the early stages of the process (Figure 7F). The bubbles around the cells were completely clean inside (Figure 7F), distinguishing them from the budding process typical of apoptosis. Thus, the morphology of cells suggests that the Ir complexes, if irradiated, induce oncosis-like cell death. In accordance with this conclusion, no noticeable increase in the caspase-3 activity was observed after incubation with the Ir complexes, while incubation with apoptosis inducer staurosporine caused a significantly increased signal (Figure S60).

### Porimin Expression and Plasma Membrane Permeability

**Figure 8:**
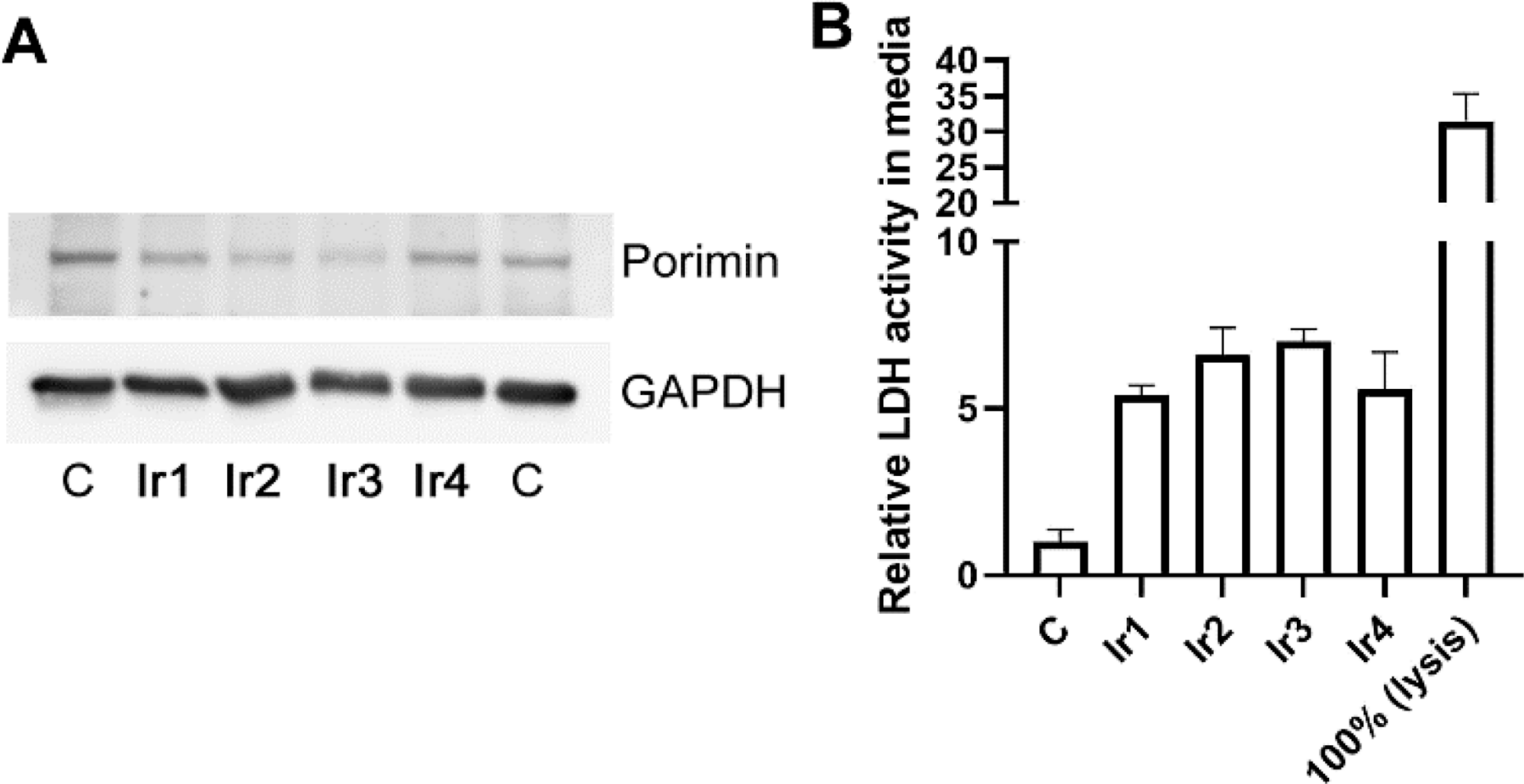
Panel A: Western Blott analysis of cellular content of porimin. A representative membrane is shown. Panel B: results of the LDH leakage assay. Data represent an average and 95% CI (confidence interval) of two independent experiments, each performed in tetraplicate. In both assays, HeLa cells were incubated with **Ir1**–**Ir4** at their equitoxic concentrations (0.3, 0.7, 1.2, and 3.7 μM for **Ir1**, **Ir2**, **Ir3**, and **Ir4**, respectively), irradiated for 1 h, and left to recover in compound-free media for 2 h.

A cell surface receptor porimin (pro-oncosis receptor) is assumed to mediate oncosis.^52^ It is responsible for abnormal membrane permeability and cell swelling in the process of oncosis.^55^ As indicated in Figure 8A, no significant increase in the expression of porimin upon incubation and irradiation of cells with Ir complexes **1**–**4** was seen. This might be explained by the fact that the total time of cells exposure to the irradiated Ir complexes was too short (1 h irradiation plus 2 h recovery) to enable the activation of the expression apparatus and the relocation of newly formed proteins into the cell membrane. However, during this short period, swelling of the cells was already clearly observed (Figure 7); it is, therefore, evident that a porimin-independent mechanism causes the phenomenon of cell swelling.

To elucidate how the cells were swollen, a further experiment was performed. During the oncotic process, the cell membrane becomes leaky due to the development of a nonselective increase in membrane permeability.^52^ Therefore, we tested the plasma membrane permeability by the lactate dehydrogenase (LDH) leakage assay (homogeneous membrane integrity assay). After the treatment and irradiation of the cells, a significant elevation of LDH signals in media was observed (approximately 5–8 times, Figure 8B), indicating an increase in membrane permeability of the cells. However, the cell membrane was not completely disintegrated, as evident from comparison with a sample of cells undergoing a complete lysis. Thus, the expanded cell volume can be related to the increase of cell membrane permeability^32^ induced by the direct membrane injury due to the photoactivity of Ir complexes.

### Intracellular Distribution

**Figure 9:**
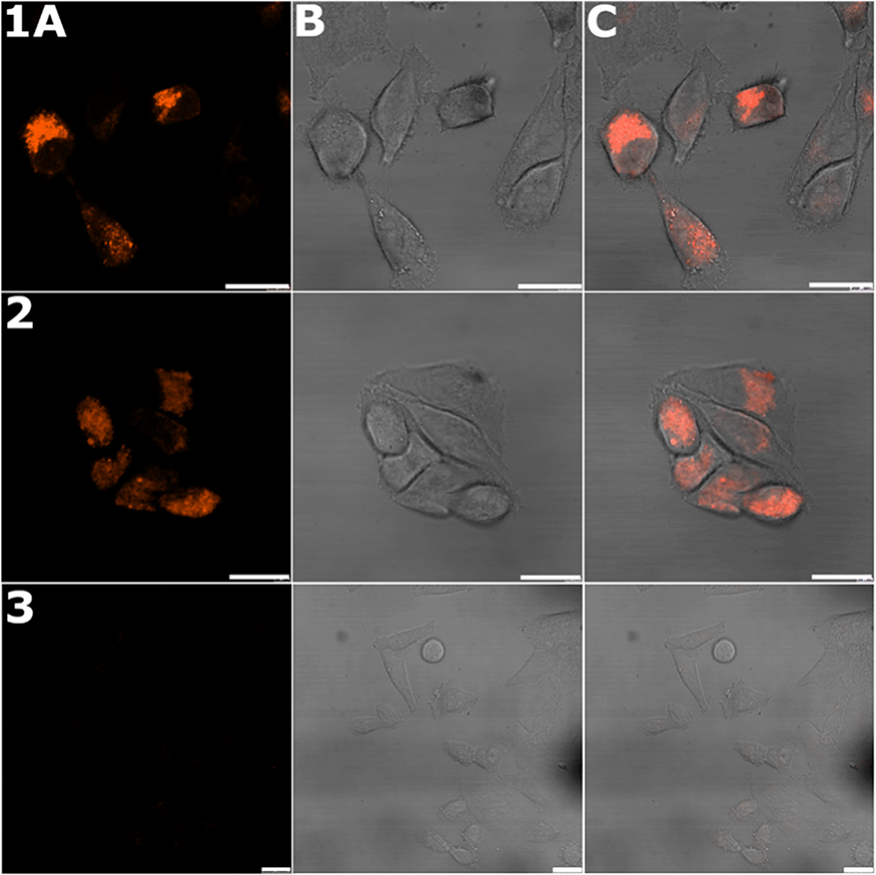
Cellular localization of Ir compounds in HeLa cells. Samples were treated for 5 h with **Ir1** or **Ir3** (2.5 μM) in the dark or left untreated. Samples: panel 1, cells treated with **Ir1**; panel 2, cells treated with **Ir3**; panel 3, untreated control cells. Channels: (A) fluorescence signal, (B) bright field, and (C) overlay of the bright field a fluorescence channels. The scale bar indicates 20 μm.

To reveal the intracellular localization of the complexes, we took advantage of the fluorescent properties of **Ir1** and **Ir3**. Laser confocal microscopic images were obtained, showing the fluorescence signal originating from the complexes localized in HeLa cells after 5 h of incubation in the dark. After removing Ir-containing media, samples were analyzed on a confocal laser-scanning microscope with excitation at 405 nm and the emission channel in the 450–750 nm range. As indicated, the signals from **Ir1** and **Ir3** were localized mainly out of the cell nucleus, with most of the signal associated with the cytoplasm (Figure 9, panels 1A and 2A). This limits the likelihood of DNA being the predominant target site of these complexes.

The specific cellular target of the complexes was determined by a colocalization assay with LysoTracker and MitoTracker dyes. As shown in Figure S61, the signal of the LysoTracker correlated well with those of the complexes, giving correlation coefficients 0.76 ± 0.07 and 0.8 ± 0.1 for **Ir1** and **Ir3**, respectively (Table S10). Colocalization with mitochondria was noticeably less prominent (Figure S62 and Table S10). The preferential lysosomal localization of the Ir complexes corresponds with endocytosis as a mechanism of cellular uptake (*vide supra*).

**Figure 10:**
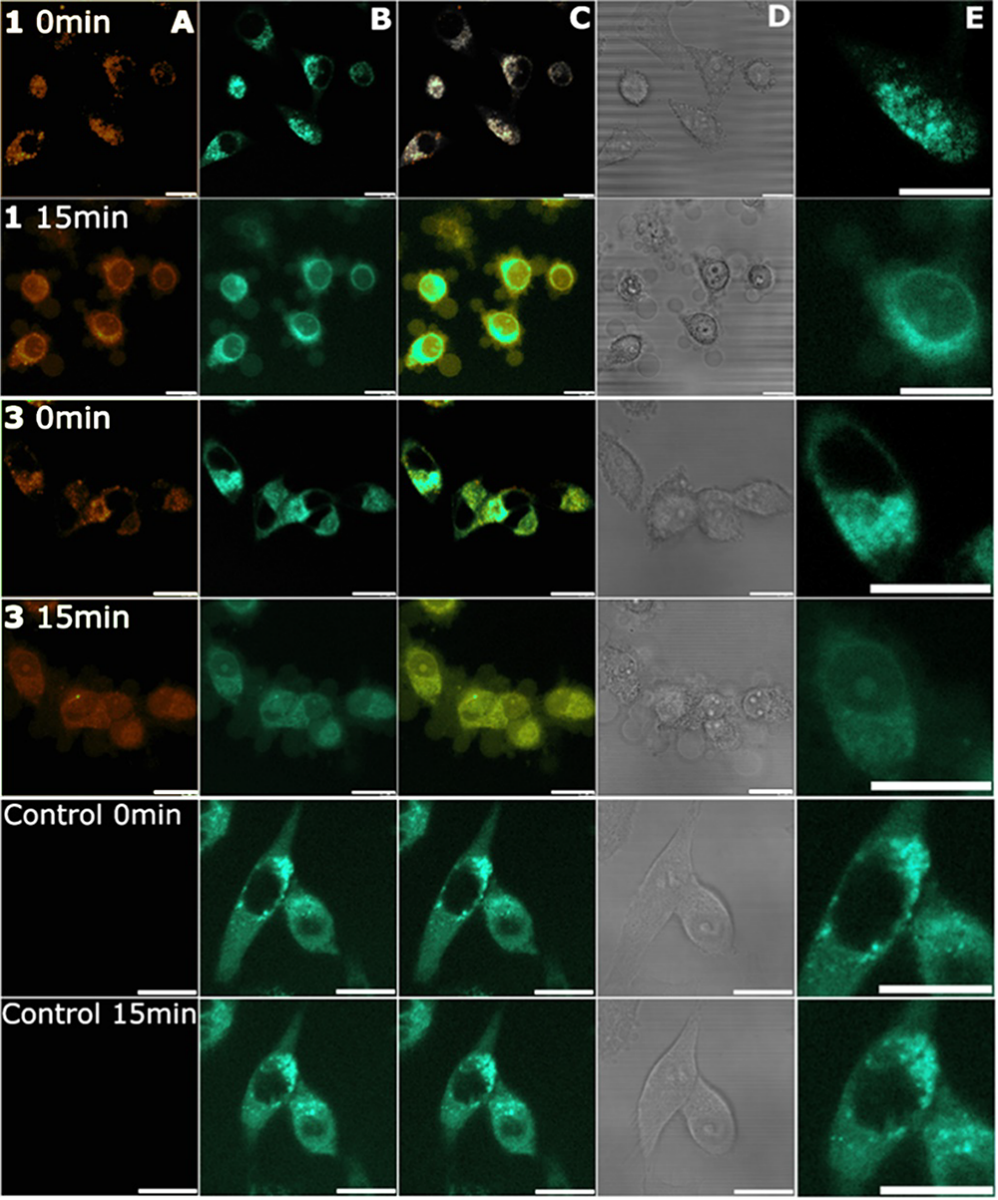
Time-lapse monitoring and colocalization of iridium compounds **Ir1** and **Ir3** with lysosomes in Hela cells determined by confocal microscopy. Cells were treated with 2 μM of tested compounds and incubated for 3 h before staining with LysoTracker. Channels: (A) fluorescence signal coming from tested iridium compounds, (B) fluorescence signal from LysoTracker Green DND-26, (C) overlay of both fluorescence channels, (D) bright field channel, (E) cropped details from the LysoTracker channel for more precise determination of changes in lysosomal morphology. The scale bar indicates 20 μm. (Note: line spacing on the panels dedicated to the bright field channel is caused by occasional faults of the CCD sensor. The beam path for fluorescence channels was not influenced by this issue).

When performing colocalization experiments, we surprisingly observed that the intensity and location of the signal originating from LysoTracker changed over time. At the beginning of the experiment, the signal was localized in distinct puncta due to localized accumulation in the acidic environment of lysosomes (Figure 10, panels “0 min”). However, a short time after exposure to the excitation light, the translocation of lysosomally localized LysoTracker into the cytosol became apparent, so a diffuse staining pattern throughout the cytosol was observed (Figure 10, panels “15 min”). This phenomenon was characteristic of the cells pretreated with Ir complexes, whereas the signal of LysoTracker in control, untreated cells steadily appeared in punctuate structures inside lysosomes, regardless of the analysis time. This observation can be interpreted to mean that the Ir complexes, when irradiated, cause lysosomal membrane permeabilization and subsequent release of lysosomal content from the lysosomal lumen into the cytosol, resulting in cytoplasm acidification. This conclusion is also supported by the fact that monitoring the release of substances selectively accumulated in lysosomes, including LysoTrackers, is one of the standard assays for detecting the permeabilization of the lysosomal membrane.^56−58^

Rapidly dividing cancer cells are strongly dependent on effective lysosomal function, and dramatic changes in lysosomal volume, composition, and cellular distribution occur during cancer transformation and progression; these changes promote the invasive growth of tumors.^59^ This makes cancer cells more sensitive to the impairment of lysosomes. Moreover, apoptosis-resistant cancer cells can still undergo lysosomal cell death.^59^ Thus, the targeting of lysosomes and induction of lysosome-dependent cell death represent promising therapeutic strategies for cancer treatment.^56,60−62^ From this point of view, the Ir complexes described in this study offer exciting potential, mainly because the disintegration of lysosomes and subsequent cellular effects can be triggered explicitly by light at the tumor site.

### Effect on 3D Spheroids

**Figure 11:**
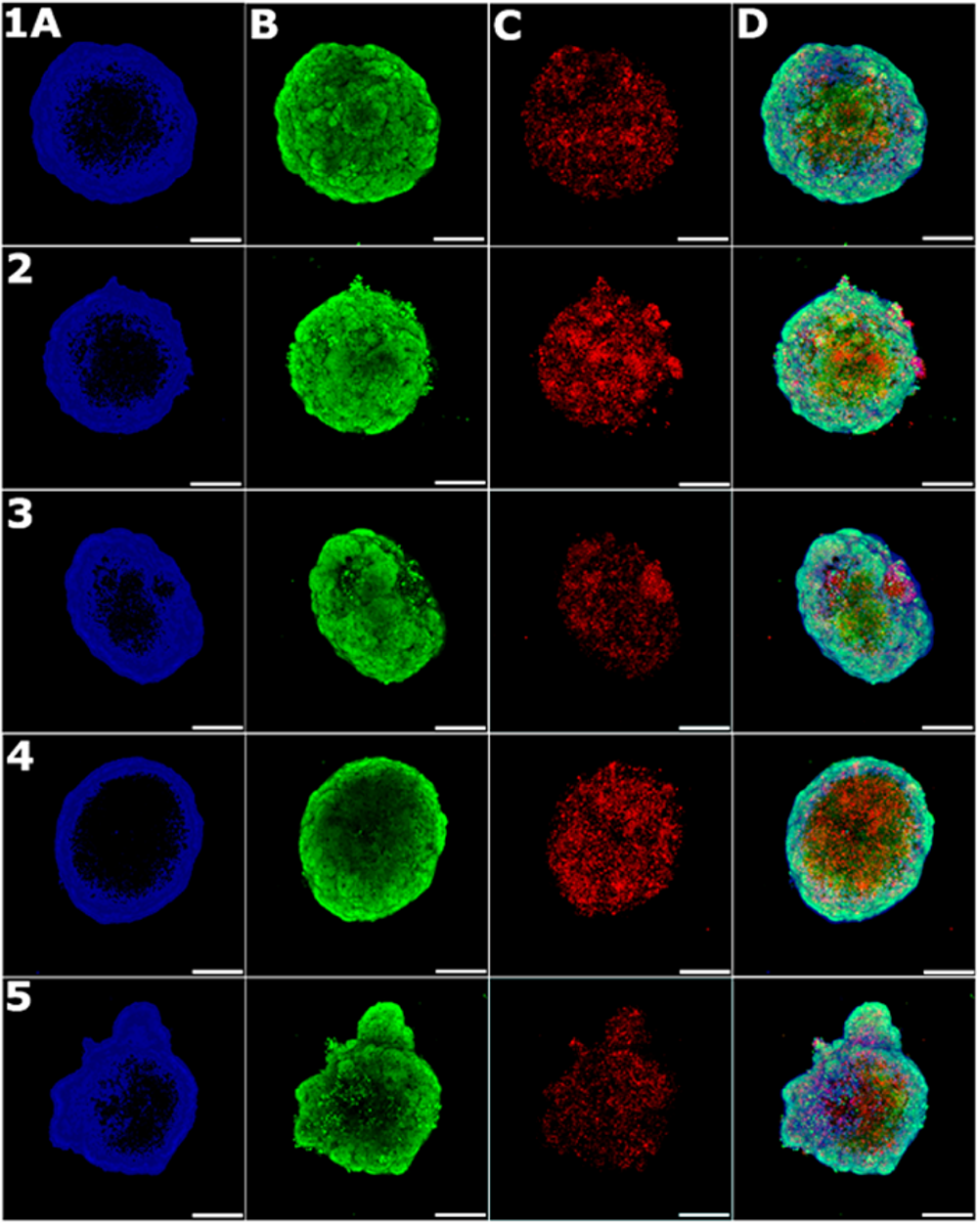
Analysis of the HeLa spheroids on confocal microscopy. Spheroids were stained with (A) Hoechst 33258 dye, (B) calcein AM, and (C) propidium iodide. Merged channels are in panel D. Samples: (1) untreated control, (2 and 3) cells treated with **Ir1** (2 μM), and (4 and 5) cells treated with **Ir3** (2 μM). Samples in panels 1, 2, and 4 were irradiated for 5 min with blue (405 nm) laser light 24 h before analysis. Samples 3 and 5 were kept in the dark. Pictures represent two independent experiments; each picture was obtained as the maximal projection from 10 z-stacks. The scale bar represents 200 μm.

Since three-dimensional (3D) cell cultures are considered to be a more representative model for *in vitro* anticancer drug screening,^63−66^ effects of the Ir complexes were also determined in the 3D culture of Hela cells. The cells were seeded on 96-well ultralow attachment U-shape plates and incubated for 72 h. Preformed spheroids were transferred as single spheres to Matrigel embed and kept for 24 h in a 3D forming culture medium. Then, the spheroids were treated with **Ir1** or **Ir3** for 5 h, washed and transferred to confocal dishes, and irradiated with 405 nm laser for 5 min (final power of 1 mW) or kept in the dark. Subsequently, spheroids were cultured for another 24 h and stained with calcein AM (a membrane-permeable live-cell labeling dye) and propidium iodide (PI, a stain for nonviable cells of disturbed cell membrane integrity) after this period. Samples were imaged on a confocal microscope in 10 z-stack scans, and images were analyzed for PI fluorescence as a measure of the proportion of dead cells in each spheroid. For correct quantitative evaluation, Hoechst staining was also applied to define the contours of spheroids in all samples precisely.^63,67,68^ The resulting representative images are shown in Figure 11.

As indicated, irradiation of the spheroid pretreated with Ir complexes **1** or **3** increased PI fluorescence (Figure 11 and Table 5), indicating an elevated number of dead cells in the spheroid volume. The increase represents 58 or 48% for spheroids treated with **Ir1** or **Ir3**, respectively, compared to the untreated irradiated control (Table 5). In contrast, the changes in propidium iodine fluorescence were insignificant when treated samples were kept nonirradiated.

**Table 5:** Analysis of the Mean PI Fluorescence Intensity^a^

sample	mean fluorescence intensity (a.u.)
control—irradiated	8.3 ± 0.4
control—dark	8.0 ± 0.4
**Ir1**—irradiated	13.1 ± 0.5
**Ir1**—dark	9.1 ± 0.2
**Ir3**—irradiated	12.3 ± 0.7
**Ir3**—dark	9.5 ± 0.3

a Fluorescence intensity was analyzed in the maximal projection of the obtained z-stacks on confocal microscopy.

The data demonstrate the capability of Ir complexes to induce cell death even in 3D spheroids, although the effect is less pronounced than that observed in cells cultured in a 2D monolayer arrangement. This may reflect properties typical for 3D but not for 2D cultures, such as impaired penetration to the cells inside 3D spheroids. As shown (Figure S63), **Ir1** was localized after a relatively short (5 h) treatment, mainly in the surface layer of the spheroid. Thus, this may represent a limiting factor that could restrict the photoactivity of tested Ir complexes in the above-mentioned experiment. Therefore, further efforts will be devoted to improving the penetration capabilities of these types of Ir complexes.

## Conclusions

In summary, we designed and synthesized novel substitution-inert, octahedral Ir(III) complexes **Ir1**–**Ir4** of the type [Ir(C^N)_2_(N^N)][CF_3_SO_3_] with a rational choice of the C^N and N^N ligands, based on the cooperation of dppz chromophore with four different cyclometalated ligands, 2-(5-arylthiophen-2-yl) benzothiazoles **HL1** and **HL2**, and 2-(5-arylthiophen-2-yl)-1-(4-(trifluoromethyl)benzyl)-1*H*-benzo[*d*]imidazoles **HL3** and **HL4**. Complexes **Ir1** and **Ir3**, containing the *p*-CF_3_C_6_H_4_ substituent on the thienyl ring, exhibited aggregation-induced emission features, whereas **Ir2** and **Ir4**, containing the *p*-Me_2_NC_6_H_4_ group, showed aggregation-caused quenching characteristics. Complexes **Ir1**–**Ir4** could oxidize NADH under blue light irradiation photocatalytically and photogenerate ^1^O_2_ and/or ^•^OH in cell-free media. Compounds **Ir1**–**Ir4** showed very low toxicity in the dark, even at the highest attainable concentration (50 μM) in cervical, melanoma, and colon cancer cells. They also showed high phototoxicity after blue light irradiation, **Ir1** being the most active with the highest phototoxicity indexes (>160). This complex also showed the highest accumulation in HeLa cells and photoinduced ROS generation. **Ir2** was also activated by green and red light, although with lower effectivity. Importantly, nonirradiated Ir compounds show very low toxicity in noncancerous human cells even with prolonged incubation, suggesting their potential as possible drug candidates for PDT.

The data presented here clearly indicate that **Ir1**–**Ir4** accumulated in the membrane of intracellular organelles, particularly lysosomes, and if irradiated, they induce a leakage of lysosomal content into the cytoplasm. This is likely to be associated with photoinduced ROS generation, as ROS can directly damage the lysosomal membranes, leading to lysosomal hydrolases and H^+^ leakage from the affected lysosomes and acidification of the intracellular environment. Subsequently, this can lead to erosion and permeabilization of the cell membrane, thus resulting in oncotic-like cell death.

Although the investigated Ir complexes show superior photoactivities in 2D cell cultures, their effects on cells in 3D arrangement are less pronounced but still evident. The reason may be poorer penetration of the complexes into the deep layers of the spheroid. This may be related to the high lipophilicity and self-assembly/aggregation of the compounds, when the molecules of the complex remain associated with the membrane components of the outer cells and thus do not penetrate the intercellular space, from where they could spread to other layers of the 3D cell culture. Further research will, therefore, focus on improving properties that will allow better photophysical properties and better penetration into three-dimensional culture.

## Experimental Section

### Reagents, Chemicals, Cell Lines, and Culture Conditions

4-Trifluoromethylphenylboronic acid, 4-(dimethylamino)phenylboronic acid, *o*-phenylenediamine, 2-aminothiophenol, 4-(trifluoromethyl)benzyl bromide, 5-bromo-2-thiophenecarboxaldehyde, potassium triflate, trifluoroacetic acid, cesium carbonate, potassium carbonate, sodium bisulfite, tetrakis(triphenylphosphine)palladium(0), and bis(triphenylphosphine)palladium(II) chloride were obtained from Sigma-Aldrich (Madrid, Spain). IrCl_3_ was obtained from Johnson Matthey. Deuterated solvents were obtained from Euriso-top. The purities ≥95% of the synthesized complexes used for biological evaluation were determined by RP-HPLC.

### Preparation of HC^N Proligands

#### Synthesis of 2-(5-Bromothiophen-2-yl)benzo[*d*]thiazole (**A**)

Intermediate **A** was synthesized using a previously described procedure.^4^ A suspension of 5-bromo-2-thiophenecarboxaldehyde (0.59 mL, 5 mmol) and sodium bisulfite (1.05 g, 10 mmol) in water (10 mL) was stirred at 80 °C for 1 h. Then, *o*-aminothiophenol (0.55 mL, 5 mmol) was dissolved in ethanol (EtOH) (10 mL), added to the reaction mixture, and stirred at 80 °C overnight. After completion of the reaction, EtOH was removed under reduced pressure, and an extraction was performed with dichloromethane (3 × 15 mL). The organic phase was dried with anhydrous magnesium sulfate, and the solvent was removed under reduced pressure. The intermediate **A** was precipitated with dichloromethane (DCM) and hexane and washed twice with hexane to obtain the final pure product.

The previously reported intermediate **A** was obtained as a pale-yellow solid (1.07 g, 72%).^69^^1^H NMR (401 MHz, chloroform-*d*, 298 K, δ ppm): 8.00 (ddd, *J* = 8.2, 1.2, 0.6 Hz, 1H), 7.84 (ddd, *J* = 8.0, 1.3, 0.7 Hz, 1H), 7.48 (m, 1H), 7.41–7.34 (m, 2H), 7.09 (d, *J* = 4.0 Hz, 1H).

#### Synthesis of 2-(5-Bromothiophen-2-yl)-1*H*-benzo[*d*]imidazole (**B**)

Intermediate **B** was synthesized using a previously described procedure.^70^ A suspension of 5-bromo-2-thiophenecarboxaldehyde (0.59 mL, 5 mmol) and sodium bisulfite (1.05 g, 10 mmol) in water (10 mL) was stirred at 80 °C for 1 h. Phenylenediamine (540 mg, 5 mmol) was dissolved in EtOH (10 mL) and added to the reaction mixture. Then, it was stirred at 80 °C overnight. EtOH was removed, and an extraction was performed with DCM (3 × 20 mL). The organic phase was dried with anhydrous magnesium sulfate, and the solvent was removed under reduced pressure to obtain the final product. Hexane was used to precipitate the intermediate **B**.

The previously reported intermediate **B** was achieved as a pale-yellow solid (315 mg, 22%).^71^^1^H NMR (300 MHz, DMSO-*d*_6_, 298 K, δ ppm): 7.64 (d, *J* = 4.0 Hz, 1H), 7.61–7.50 (m, 2H), 7.36 (d, *J* = 3.9 Hz, 1H), 7.26–7.11 (m, 2H).

#### Synthesis of 2-(5-Bromothiophen-2-yl)-1-(4-(trifluoromethyl)benzyl)-1*H*-benzo[*d*]imidazole (**B1**)

Intermediate **B1** was synthesized using a procedure described previously by us.^24^ Intermediate **B** (180 mg, 0.65 mmol) and 4-trifluoromethylbencil bromide (161 mg, 0.67 mmol) were dissolved in acetonitrile. Once dissolved, Cs_2_CO_3_ (410 mg, 1.26 mmol) was added and stirred at room temperature for 24 h. After the completion of the reaction, the mixture reaction was filtered into Celite to remove the excess salts, and the solvent was removed under reduced pressure. Intermediate **B1** was precipitated and washed with hexane.

##### Intermediate **B1**

White solid. Isolated yield: 178 mg (63.2%). ^1^H NMR (401 MHz, chloroform-*d*, 298 K, δ ppm): 7.85 (d, *J* = 8.1, 1H), 7.61 (d, *J* = 8.1, 2H), 7.33 (ddd, *J* = 8.2, 7.2, 1.3 Hz, 1H), 7.30–7.25 (m, 1H), 7.23 (s, 1H), 7.22–7.17 (m, 2H), 7.03 (d, *J* = 4.0 Hz, 1H), 6.97 (d, *J* = 4.0 Hz, 1H), 5.61 (s, 2H). ESI-MS (positive mode, CHCl_3_): *m*/*z* = 436.9935 (M+H)^+^, calcd *m*/*z* = 435.9851 [M]^+^.

#### Synthesis of 2-(5-(4-(Trifluoromethyl)phenyl)thiophen-2-yl)benzo[*d*]thiazole (**HL1**)

Intermediate **A** (296.23 mg, 1 mmol), 4-trifluoromethylphenylboronic acid (285 mg, 1.5 mmol), Pd(PPh_3_)_4_ (58 mg, 0.05 mmol), and K_2_CO_3_ (414.63 mg, 3 mmol) were dissolved in 6 mL of toluene:H_2_O 2:1 and stirred under microwave at 120 °C for 1 h. After the completion of the reaction, water and dichloromethane were added, and an extraction was performed. The organic phase was dried using anhydrous magnesium sulfate, and the solvent was removed under reduced pressure. The final compound was precipitated and washed with hexane.

##### **HL1**

Gold-green bright solid. Isolated yield: 61% (163 mg, 0.772 mmol). ^1^H NMR (401 MHz, chloroform-*d*, 298 K, δ ppm): 8.05 (d, *J* = 8.2, 1H), 7.91–7.84 (m, 1H), 7.77 (d, *J* = 8.0, 2H), 7.68 (d, *J* = 8.1 Hz, 2H), 7.64 (d, *J* = 3.9, 1H), 7.50 (m, 1H), 7.44–7.36 (m, 2H). ^19^F NMR (377 MHz, DMSO, 298 K, δ ppm): −61.10.

#### Synthesis of 4-(5-(Benzo[*d*]thiazol-2-yl)thiophen-2-yl)-*N*,*N*-dimethylaniline (**HL2**)

The synthetic procedure was the same as for HL1, using 4-(*N*,*N*-dimethylamino)phenylboronic acid (198 mg, 1.2 mmol). The purification method was also the same.

The previously reported proligand **HL2** was achieved as a yellow solid (212.6 mg, 63.8%).^36^^1^H NMR (401 MHz, chloroform-*d*, 298 K, δ ppm): 8.04–7.96 (m, 1H), 7.86–7.80 (m, 1H), 7.61–7.53 (m, 3H), 7.50–7.42 (m, 1H), 7.34 (m, 1H), 7.18 (d, *J* = 3.9 Hz, 1H), 6.87–6.49 (m, 2H), 3.02 (s, 6H).

#### Synthesis of 1-(4-(Trifluoromethyl)benzyl)-2-(5-(4-(trifluoromethyl)phenyl)thiophen-2-yl)-1*H*-benzo[*d*]imidazole (**HL3**)

A suspension of intermediate **B1** (219 mg, 0.5 mmol), 4-trifluoromethylphenylboronic acid (99.53 mg, 0.55 mmol), PdCl_2_(PPh_3_)_2_ (17.5 mg, 0.025 mmol), and K_2_CO_3_ (207 mg, 1.5 mmol) in 6 mL of mixture dioxane:H_2_O 4:2 was stirred at 130 °C for 1 h. After the completion of the reaction, water and DCM were added, and an extraction was performed (3 × 20 mL). The organic phase was dried with anhydrous sulfate magnesium, and the solvent was removed under reduced pressure. **HL3** was precipitated and washed with hexane.

##### Proligand **HL3**

White solid. Isolated yield: 36% (90 mg). ^1^H NMR (401 MHz, chloroform-*d*,298 K, δ ppm): 7.89 (d, *J* = 8.0 Hz, 1H), 7.71 (d, *J* = 8.3 Hz, 2H), 7.67–7.59 (m, 4H), 7.39–7.31 (m, 2H), 7.31–7.24 (m, 3H), 7.23–7.18 (m, 1H), 5.68 (s, 2H). ^13^C NMR (101 MHz, CDCl_3_, 298 K, δ ppm): 147.4, 145.6, 143.1, 139.9, 136.7, 136.2, 132.4, 130.6, 130.3, 130.2, 129.87, 128.4, 126.3, 126.3, 126.2, 126.1, 126.1, 126.0, 125.1, 123.8, 123.4, 120.2, 109.7, 47.9. ^19^F NMR (377 MHz, CDCl_3_, 298 K, δ ppm) −62.66, −62.70. ESI-MS (positive ion mode, CHCl_3_): *m*/*z* = 503.10 [M + H]^+^, calcd *m*/*z* = 502.09 [M]^+^.

#### Synthesis of *N*,*N*-dimethyl-4-(5-(1-(4-(trifluoromethyl)benzyl)-1*H*-benzo[*d*]imidazol-2-yl)thiophen-2-yl)aniline (**HL4**)

The synthesis of HL4 was the same as for HL3 but using 4-(N,N-dimethylamino)phenylboronic acid.

##### Proligand **HL4**

Yellow-green solid. Isolated yield: 197 mg (82%). ^1^H NMR (401 MHz, chloroform-*d*, 298 K, δ ppm): 7.86 (d, *J* = 8.0, 1H), 7.61 (d, *J* = 8.1 Hz, 2H), 7.54–7.45 (m, 2H), 7.31 (ddt, *J* = 8.1, 7.0, 1.2 Hz, 1H), 7.27 (s, 1H), 7.26–7.21 (m, 2H), 7.19–7.13 (m, 2H), 7.08 (dd, *J* = 3.9, 1.1 Hz, 1H), 6.76–6.67 (m, 2H), 5.66 (s, 2H), 2.99 (s, 6H). ^13^C NMR (101 MHz, CDCl_3_, 298 K, δ ppm): 150.5, 149.1, 148.3, 143.2, 140.2, 136.2, 128.5, 128.3, 127.0, 126.3, 126.2, 126.2, 123.2, 123.1, 121.5, 121.4, 119.9, 112.4, 109.5, 47.9, 40.3. 19F NMR (377 MHz, CDCl_3_, 298 K, δ ppm): −62.63. ESI-MS (positive ion mode, CHCl_3_): *m*/*z* = 478.16 [M + H]^+^, calcd *m*/*z* = 477.15 [M]^+^.

### Preparation of New Ir(III) Complexes

#### Synthesis of Dimer Complexes [Ir(C^N)_2_(μ-Cl)]_2_

The dimeric iridium(III) precursor was synthesized as previously published. IrCl_3_·H_2_O (50 mg, 0.16 mmol) and the corresponding proligands **HL1**–**HL4** (0.35 mmol) were dissolved in 8 mL of 2-ethoxyethanol:H_2_O 3:1 mixture and stirred under a nitrogen atmosphere at 110 °C for 48 h (**HL1**–**HL3**) or 24 h (**HL4**). After the completion of the reaction, the reaction was cooled down to room temperature, and water was added (10 mL). Orange to red precipitates were filtered and washed with cooled water. In the case of **HL4**, the dimeric precursor was soluble. The solvent was removed under reduced pressure and recrystallized with MeOH/ethyl ether. Products were used in the following reaction without further purification.

#### Synthesis of Monomeric Complexes [Ir(C^N)_2_(dppz)]OTf

The corresponding dimeric iridium(III) precursor (1 equiv), dppz (2.1 equiv), and potassium triflate (2.5 equiv) were added into a Schlenk flask and dissolved in 10 mL of MeOH:DCM (3:2) mixture. The mixture reaction was stirred at 58 °C for 24 h. After finishing the reaction, it was cooled to room temperature, and the solvent was removed under reduced pressure. Pure products were obtained after an alumina column using DCM:CH_3_CN 1:1 as an eluent. Pure tubes were collected, and the solvent was removed under reduced pressure. Finally, the new iridium complexes were recrystallized with DCM and hexane and washed several times with hexane to obtain the final pure iridium complex.

#### Complex **Ir1**

Yellow solid. Isolated yield: 37% (57 mg). ^1^H NMR (401 MHz, acetonitrile-*d*_3_, 298 K, δ ppm): 9.61 (dt, *J* = 8.3, 1.2 Hz, 2H), 8.65 (dt, *J* = 5.3, 1.2 Hz, 2H), 8.33 (m, 2H), 8.11–8.05 (m, 2H), 8.02 (ddd, *J* = 8.3, 5.3, 0.8 Hz, 2H), 7.91 (d, *J* = 8.0 Hz, 2H), 7.57 (d, *J* = 8.8 Hz, 4H), 7.52 (d, *J* = 8.4 Hz, 4H), 7.20 (ddt, *J* = 8.2, 7.3, 1.0 Hz, 2H), 6.92 (ddt, *J* = 8.4, 7.3, 1.1 Hz, 2H), 6.84 (d, *J* = 0.8 Hz, 2H), 6.06–5.96 (m, 2H). ^13^C NMR (101 MHz, CD_3_CN, 298 K, δ ppm): 173.5, 158.5, 153.2, 151.3, 149.6, 149.0, 142.4, 139.1, 136.2, 135.6, 133.9, 132.3, 131.6, 130.5, 129.8, 129.4, 129.2, 129.1, 128.3, 127.8, 126.3, 125.6, 125.6, 125.5, 125.5, 125.1, 125.0, 123.6, 122.4, 116.2. ^19^F NMR (377 MHz, CD_2_Cl_2_, 298 K, δ ppm): −63.09, −78.93. ESI-MS (positive ion mode): calc.: [M-CF_3_SO_3_]^+^=1195.0792 *m*/*z*; exp: 1195.0796 *m*/*z*. Anal. Calcd for C_55_H_28_F_9_IrN_6_O_3_S_5_: C, 49.14; H, 2.10; N, 6.25; S, 11.93. Found: C, 49.36; H, 2.22; N, 6.30; S, 11.72 (%).

#### Complex **Ir2**

Reddish solid. Isolated yield: 24% (41 mg). ^1^H NMR (401 MHz, chloroform-*d*, 298 K, δ ppm): 9.91 (dd, *J* = 8.2, 1.5 Hz, 2H), 8.64 (dd, *J* = 5.2, 1.5 Hz, 2H), 8.43 (dd, *J* = 6.6, 3.4 Hz, 2H), 8.20 (dd, *J* = 8.3, 5.3 Hz, 2H), 8.05 (dt, *J* = 6.6, 3.2 Hz, 2H), 7.71 (d, *J* = 8.2, 2H), 7.40–7.31 (m, 4H), 7.12 (ddd, *J* = 8.1, 7.3, 1.1 Hz, 2H), 6.84 (ddd, *J* = 8.5, 7.3, 1.2 Hz, 2H), 6.63–6.55 (m, 4H), 6.44 (s, 2H), 5.88 (d, *J* = 8.4 Hz, 2H), 2.96 (s, 12H). ^13^C NMR (75 MHz, CDCl_3_, 298 K, δ ppm): 161.6, 158.1, 154.0, 152.0, 150.4, 144.2, 139.9, 137.5, 133.6, 132.1, 131.8, 130.9, 129.3, 128.9, 128.7, 126.9, 125.5, 124.5, 116.3, 113.0, 41.1. ^19^F NMR (377 MHz, CD_2_Cl_2_, 298 K, δ ppm): −78.91. ESI-MS (positive ion mode): calc.: [M-CF_3_SO_3_]^+^= 1145.1888 *m*/*z*; exp: 1145.1893 *m*/*z*. Anal. Calcd for C_57_H_40_F_3_IrN_8_O_3_S_5_: C, 52.89; H, 3.11; N, 8.66; S, 12.38. Found: C, 52.83; H, 3.27; N, 8.73; S, 12.59 (%).

#### Complex **Ir3**

Yellow solid. Isolated yield: 44% (77.5 mg). ^1^H NMR (401 MHz, chloroform-*d*, 298 K, δ ppm): 9.92 (dd, *J* = 8.3, 1.5 Hz, 2H), 8.79 (dd, *J* = 5.2, 1.5 Hz, 2H), 8.43 (dt, *J* = 6.2, 3.1 Hz, 2H), 8.21 (dd, *J* = 8.2, 5.2 Hz, 2H), 8.09–7.99 (m, 2H), 7.52–7.48 (m, 12H), 7.43–7.32 (m, 6H), 7.11 (ddd, *J* = 8.3, 7.4, 1.0 Hz, 2H), 6.78 (ddd, *J* = 8.4, 7.4, 1.0 Hz, 2H), 6.64 (s, 2H), 6.06 (d, *J* = 17.1 Hz, 2H), 5.92 (d, *J* = 17.1 Hz, 2H), 5.58 (dt, *J* = 8.3, 0.9 Hz, 2H). ^13^C NMR (101 MHz, CDCl_3_, 298 K, δ ppm): 161.2, 156.8, 153.9, 150.4, 150.2, 143.1, 140.1, 139.1, 139.0, 136.4, 136.1, 133.8, 132.7, 131.0, 130.7, 130.5, 130.2, 129.9, 128.8, 128.0, 127.1, 126.3, 126.2, 126.2, 125.9, 125.9, 125.1, 125.0, 124.8, 123.8, 123.6, 122.4, 122.3, 112.5, 111.5, 48.8. ^19^F NMR (377 MHz, CD_2_Cl_2_, 298 K, δ ppm): −63.13, −63.14, −78.91. ESI-MS (positive ion mode): calc.: [M-CF_3_SO_3_]^+^= 1477.2255 *m*/*z*; exp: 1477.2277 *m*/*z*. Anal. Calcd for C_71_H_40_F_15_IrN_8_O_3_S_3_: C, 52.43; H, 2.48; N, 6.89; S, 5.91. Found: C, 52.47; H, 2.80; N, 7.00; S, 5.96 (%).

#### Complex **Ir4**

Brown solid. Isolated yield: 32% (44 mg) ^1^H NMR (401 MHz, chloroform-*d*) δ 9.92 (dd, *J* = 8.2, 1.5 Hz, 2H), 8.85 (dd, *J* = 5.2, 1.5 Hz, 2H), 8.46 (dd, *J* = 6.6, 3.4 Hz, 2H), 8.15 (dd, *J* = 8.2, 5.2 Hz, 2H), 8.06 (dt, *J* = 6.9, 3.5 Hz, 2H), 7.53 (d, *J* = 8.1 Hz, 4H), 7.42 (d, *J* = 8.1 Hz, 4H), 7.28 (m, 4H), 7.03 (t, *J* = 7.8 Hz, 2H), 6.71 (t, *J* = 7.8 Hz, 2H), 6.64–6.52 (m, 4H), 6.43 (s, 2H), 6.00 (d, *J* = 16.9 Hz, 2H), 5.80 (d, *J* = 17.0 Hz, 2H), 5.51 (d, *J* = 8.2 Hz, 2H), 2.95 (s, 12H). ^13^C NMR (101 MHz, CDCl_3_) δ 162.14, 158.73, 154.18, 154.06, 150.96, 150.66, 143.27, 140.67, 139.44, 139.35, 135.84, 133.81, 132.69, 130.73, 130.04, 127.93, 127.33, 127.31, 126.43, 126.39, 125.31, 124.39, 122.84, 120.91, 119.48, 112.21, 111.14, 48.74, 40.27. ^19^F NMR (377 MHz, CD_2_Cl_2_, 298 K, δ ppm): −62.92, −78.95. ESI-MS (positive ion mode): calc.: [M-CF_3_SO_3_]^+^=1427.3351 *m*/*z*; exp: 1427.3363 *m*/*z*). Anal. Calcd for C_74_H_56_F_9_IrN_10_O_3_S_3_: C, 55.81; H, 3.54; N, 8.79; S, 6.04. Found: C, 55.88; H, 3.41; N, 8.90; S, 6.12 (%).

#### Methods and Instrumentation

##### Microwave

The last step in the synthetic route for ligands was done in an Anton Paar Monowave 50 (315 W) microwave.

##### Nuclear Magnetic Resonance (NMR) Spectroscopy

The ^1^H, ^13^C{^1^H}, and bidimensional NMR spectra were recorded on a Bruker AC 300E, Bruker AV 400, or Bruker AV 600 NMR spectrometer, and chemical shifts were determined by reference to the residual ^1^H and ^13^C{^1^H} solvent peaks.

##### Elemental Analysis

The C, H, N, and S analyses were performed with a Carlo Erba model EA 1108 microanalyzer with EAGER 200 software.

##### Mass Spectrometry (MS)

ESI mass (positive mode) analyses were performed on an RP/MS TOF 6220. The isotopic distribution of the heaviest set of peaks matched very closely to that calculated for formulating the complex cation in every case.

##### Photophysical Characterization

UV/vis spectroscopy was performed on a PerkinElmer Lambda 750 S spectrometer with operating software. Solutions of all complexes were prepared in acetonitrile and water (1% DMSO) at 10 μM. The emission spectra were obtained with a Horiba Jobin Yvon Fluorolog 3-22 modular spectrofluorometer with a 450 W xenon lamp. Measurements were performed in a right-angled configuration using 10 mm quartz fluorescence cells for solutions at 298 K. Emission lifetimes (τ) were measured using an IBH FluoroHub TCSPC controller and a NanoLED (372 nm) pulse diode excitation source (τ <10 μs); the estimated uncertainty is ±10% or better. Emission quantum yields (Φ) were determined using a Hamamatsu C11347 absolute PL quantum yield spectrometer; the estimated uncertainty is ±10% or better. Solutions of all complexes were prepared in acetonitrile and water (1% DMSO) at 10 μM. For lifetimes and quantum yield measurements, the samples in acetonitrile were previously degassed by bubbling argon for 30 min.

##### X-Ray Structure Determinations

Intensities were registered at low temperatures on a Bruker D8QUEST diffractometer using monochromated Mo *K*α radiation (λ = 0.71073 Å). Absorption corrections were based on multiscans (program SADABS).^72^ Structures were refined anisotropically using SHELXL-2018.^73^ Hydrogen atoms were included using rigid methyl groups or a riding model.

Special features: the structure contains poorly resolved regions of residual electron density; this could not be adequately modeled, and so was “removed” using the program SQUEEZE, which is part of the PLATON system.^74^ The void volume per cell was 322 eÅ^3^ with a void electron count per cell of 150. This additional solvent was not considered when calculating derived parameters, such as the formula weight, because the nature of the solvent was uncertain. Three of the four CF_3_ ligands are disordered over two positions, ca. 66:44%, 82:18% and 90:10% each. For these ligands, appropriate SHELXL commands like SADI and RIGU were used.

##### NADH Photooxidation

Reactions between the Ir(III) complexes and NADH (100 μM) were monitored by UV/vis in the dark and under irradiation with blue light (465 nm, 4.2 mW cm^–2^), green light (520 nm, 2.0 mW cm^–2^), or red light (620 nm, 15 mW cm^–2^) in PBS (5% DMF). TON is defined as the number of moles of NADH that Ir complex could convert in 7 or 12 min, whereas TOF was calculated as the ratio of the concentration of oxidized NADH to the concentration of the compound (1 μM in the case of blue light and 5 μM for green and red light). The concentration of NADH at 339 nm was obtained using the value of the molar extinction coefficient (ε_339_ = 6220 M^–1^ cm^–1^).

##### Singlet Oxygen Quantum Yields

Singlet oxygen quantum yields were calculated in aerated acetonitrile solution using DPBF as a chemical trap upon blue light irradiation and using [Ru(bpy)_3_]Cl_2_ as a reference. Photolysis of DPBF in the presence of iridium complexes was monitored by UV/vis, and the absorbance of DPBF at 411 was plotted against irradiation times and slopes calculated. Finally, singlet oxygen quantum yields were calculated using the following equation:
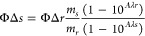
where ΦΔ_*r*_ is the singlet oxygen quantum yield of the reference, as said [Ru(bpy)_3_]Cl_2_ (Φ_Δ*r*_ = 0.57 in acetonitrile), *m_s_* and *m_r_* are the slopes of complexes and the reference, and *A*_λs_ and *A*_λr_ are the absorbance of the compounds and of the reference at the irradiation wavelength, respectively.

##### Hydroxyl Radical Generation

All compounds (10 μM) were prepared in PBS (5% DMF). To this solution, HPF was added with a final concentration of 10 μM. Then, samples were irradiated by blue light (465 nm, 4.2 mW cm^–2^) for indicated time intervals. Fluorescence spectra were obtained with a Horiba Jobin Yvon Fluorolog 3-22 modular spectrofluorometer with a 450 W xenon lamp. Measurements were performed in a right-angled configuration using 10 mm quartz fluorescence cells for solutions at 298 K. The excitation wavelength was set to 490 nm, and the excitation and emission slit widths were 3 nm.

##### Cell Lines, Culture Conditions, and Stock Solution Preparation

A375 human skin melanoma cells and HeLa human cervix adenocarcinoma cells were purchased from ECACC (UK). HCT166 and MRC5pd30 were obtained from ATCC, Manassas, VA, USA. A375, HeLa, HCT116, and MRC5 cells were cultured in the DMEM growth medium (high glucose, 4.5 g L^–1^, Biosera) supplemented with gentamycin (50 mg mL^–1^) and 10% heat-inactivated FBS (Biosera); media for the MRC5 cells were further enriched by 1% nonessential amino acids (Sigma-Aldrich, Prague, Czech Republic). A human telomerase reverse transcriptase (hTERT)-immortalized EP156T prostatic epithelial cell line was purchased from the American Type Culture Collection (CRL3289, ATCC, Manassas, VA, USA).

For the biological experiments, the stock solutions of Ir complexes were prepared in DMSO and further diluted to the EBSS or DMEM medium. The final concentration of DMSO in biological experiments did not exceed 1%.

### Phototoxicity Testing

The phototoxic potency of Ir complexes **1**–**4** was determined against human cancer Hela, A375, and HCT116 cell lines. Cells were seeded on 96-well tissue culture plates at a density of 5 × 10^3^ cells/well in 100 μL of complete DMEM medium and cultured overnight in a humidified incubator. The medium was then removed, the tested compounds diluted in EBSS (EBSS = Earle’s Balanced Salt Solution) were added to the cells, and these were then incubated for 60 min in the dark. Control cells were incubated with complex-free EBSS containing the same concentration of DMSO (always less than 1%) as in the cells treated with Ir complexes. It was verified that this concentration of DMSO in vehicle controls did not affect the viability of cells. Subsequently, the cells were irradiated or sham irradiated for 1 h. The cells were irradiated using an LZC-4 photoreactor (Luzchem Research, Gloucester, Canada) equipped by 16 lamps LZC-420 with a maximum centered at 420 nm. Afterward, the EBSS medium with Ir complexes was removed, and cells were cultured for 72 h in a drug-free complete DMEM medium. The number of cells was determined using a standard MTT or SRB assay. The IC50 values were obtained from dose–response curves. The phototoxic index (PI) was calculated as a ratio of IC_50_ (dark)/IC_50_ (irradiated).

To assess the long-term effect on noncancerous cells, human MRC5 and hTERT EP156T cells seeded in 96-well plates at a density of 4 x10^3^ cells/well were incubated for 48h in a complete DMEM medium containing **Ir1**–**Ir4**. After the period of incubation, an MTT assay was performed and evaluated.

### Intracellular Accumulation

The level of Ir accumulated in HeLa cells treated with tested compounds at their equimolar concentrations (3 μM) for 2 h at 37 °C was measured as already described^24,75,76^ by ICP-MS (Agilent Technologies, CA, USA). To assess the impact of endocytosis inhibitors, cells were pretreated with chloroquine (0.1 mM) or methyl-β-cyclodextrin (20 μM) for 1 h and subsequently incubated with Ir complex (3 μM) for 2 h in the dark at 37 °C.

### Determination of Intracellular ROS

HeLa cells seeded on 96-well plates at a density of 1 × 10^4^ cells per well were treated with tested compounds in EBSS at indicated equimolar concentrations and irradiated as described above. Afterward, the Ir-containing EBSS was removed, the cells were washed with PBS and harvested by trypsinization, and 5 μM CellROXDeep Red reagent (Life Technologies) was added to the cells and incubated for 30 min at 37 °C. Cells were then washed with PBS, and the fluorescence intensity (λexc: 640 nm, λemis: 660 nm) was analyzed by flow cytometer (BD FACS Verse). Data were analyzed using FCS Express 6 (DeNovo software; Glendale, CA). It was verified that the free complexes (in cell-free media) do not contribute to the final fluorescence signal.

### Cell Death Detection

Hela cells were seeded at a 6-well plate at the density of 1.5 × 10^5^ cells/well, left to sit overnight, treated with indicated concentrations of Ir complexes, and then irradiated as described above. Then, the Ir-containing EBSS was removed, and cells were incubated for a further 22 h in drug-free media. Afterward, the cells were collected by trypsinization, washed in PBS (4 °C), and stained with PI (1 μg mL^–1^) and Annexin V PacificBlue (5 μL per 100 μL of the cell suspension, Thermo Fischer Scientific) for 15 min at room temperature. Immediately after the staining, cells were analyzed by flow cytometry (BD FACSVerse), and data were analyzed using FCS Express 6 software (DeNovo software; Glendale, CA). Dot plots representative of three independent experiments are shown.

### Confocal Microscopy

HeLa cells were seeded on 35 mm glass bottom confocal culture dishes (Mattek Co., MA, USA) at 1.5 × 10^5^ cells/dish density and incubated overnight. Then, the cells were treated with tested compounds **1** and **3** (2.5 μM) in a phenol red-free medium and incubated for 5 h. After incubation, cells were washed twice with PBS and incubated in a drug-free culture medium. Subsequently, samples were analyzed on a confocal laser-scanning microscope Leica TCS SP5 (Leica Microsystems GmbH, Wetzlar, Germany). The investigated Ir complexes were excited at 405 nm, and the emission was detected in the 450–750 nm range.

### Caspase-3/7 Activity Assay

The activation of caspase-3 was detected using CellEvent Caspase-3/7 Green - Active Caspase-3/7 Assay Kit (Thermo Fisher Scientific). Briefly, HeLa cells were seeded at a 6-well plate at 3 × 10^5^ cells/well density and treated and irradiated as described above (1 h preincubation in the dark, 1 h irradiation at 420 nm). After 2 h of recovery in compound-free media, cells were stained with the CellEventCaspase 3/7 Green Detection Reagent according to the manufacturer’s protocol, and the fluorescence signal was analyzed by flow cytometry. It was verified that fluorescence of Ir complexes **1** and **3** does not interfere with the signal.

### Western Blotting

HeLa cells were treated and irradiated as described above (1 h treatment in the dark and 1 h irradiation). After irradiation, the complex containing EBSS was removed, and cells were incubated in cell-free media for 2 h. The cells were then scraped, washed with PBS, pelleted by centrifugation, and lysed for 40 min with ice-cold RIPA buffer supplemented with phenylmethylsulfonyl fluoride (PMSF), sodium orthovanadate, and protease inhibitor cocktail according to the manufacturer’s protocol (Santa Cruz Biotechnology, INC.) The resulting extracts were cleared (15,000*g,* 10 min) and combined with 2 × LBS buffer (4% sodium dodecyl sulfate (SDS); 10% 2-mercaptoethanol; 20% glycerol; 0.125 M Tris.HCl and 0.004% bromophenol blue) and heated for 5 min at 95 °C. The samples were separated by SDS-PAGE (4–15%; Mini-PROTEAN TGXTM Precast Gels) and transferred to PVDF membrane, and porimin and GAPDH were detected using specific primary antibodies (Anti-Porimin (G2) (Santa Cruz Biotechnology, sc-377295), Anti-GAPDH antibody (Sigma-Aldrich, G8795; 1:200)) and secondary antibodies (Goat Anti-Rabbit IgG (HRP) (Abcam, ab205718; 1:1000), and Goat Anti-Mouse IgG (HRP) (ThermoFisher Scientific, 32430). After the substrate (SignalFireTM ECL Reagent A+B) was added, the luminescence was recorded with the Amersham Imager 680. The quantitative evaluation was performed using Aida image software.

### Membrane Integrity Assay

HeLa cells were seeded at a 6-well plate at 2 × 10^5^ cells/well density and treated and irradiated as described above. After 2 h of recovery in cell-free media, the medium was removed (10 μL) and transferred to a black 96-well plate. To determine LDH activity in the media, the CytoTox-ONE Homogeneous Membrane Integrity Assay (Promega) was used according to the manufacturer’s protocol.

### Intracellular Localization

HeLa cells were seeded on the 35 mm confocal Petri dishes (Mattek) at 1.5 × 10^5^ cells/dish density and incubated overnight. Then, the cells were treated with 2 μM of tested compounds and incubated for 3 h. After that, samples were stained with MitoTracker Red FM or LysoTracker Green DND-26 (Thermo Fisher Scientific). Samples stained with MitoTracker were fixed with 3.7% paraformaldehyde before scanning, whereas samples stained with LysoTracker were scanned under continuous incubation at 37 °C, 5% CO_2_. Colocalization studies were analyzed on the confocal microscope Leica CM SP5 (Wetzlar, Germany), and further image processing and calculations of Pearson’s colocalization coefficients were done using ImageJ software.

Scanning details for mitochondrial colocalization: Tested compounds were excited with a 405 nm blue laser (1 mW), whereas the MitoTracker Red FM probe was excited by supercontinuum WLL at 600 nm (0.2 mW). Samples were excited sequentially in the frame-switching mode to eliminate a possible fluorescence overlap. Detection windows were 600–650 nm for tested Ir compounds and 650–700 nm for the MitoTracker Red FM probe; both fluorescence channels were detected by separate PMT detectors.

Scanning details for lysosomal colocalization: Initial irradiation by 405 nm blue light laser was 5 s at the power of 3 mW. Then, tested compounds were excited with a 405 nm blue laser (1 mW), or the LysoTracker Green DND-26 probe was excited by supercontinuum WLL at 488 nm (0.2 mW). Samples were excited sequentially in the frame-switching mode to eliminate possible fluorescence overlap. Detection windows were 600–650 nm for tested Ir compounds and 500–550 nm for the LysoTracker probe; both fluorescence channels were detected by separate PMT detectors. Images were acquired every 5 min, and images for time 0 min were obtained immediately after the first irradiation. Untreated controls were used to check nonoverlapping fluorescence confocal scanning and the impact of 405 nm blue light irradiation on the lysosomal, cellular, and subcellular morphology.

### Spheroid Irradiation and Analysis on Confocal Microscope

HeLa cells were seeded on 96w ultralow attachment U-shape plates (Corning) at the density of 5000 cells/well in the 3D forming medium: DMEM-F12 ham medium supplemented with growth and spheroid forming factors: 2% B27 (Thermo Fisher Scientific Inc., MA, USA), epidermal growth factor (EGF; Sigma-Aldrich, Germany, 20 ng mL-1), fibroblast growth factor (FGF2; Sigma-Aldrich, Germany, 10 ng mL^–1^), and bovine serum albumin (BSA) (Sigma-Aldrich, Germany, 0.15%). After 72 h of incubation, preformed spheroids were transferred as single spheres to Matrigel embed (30 min of embedding) and kept for 24 h in a 3D forming culture medium. Then, the spheroids were treated with tested compounds at the concentration of 2 μM for 5 h, and following that, the spheroids were washed and transferred to confocal 35 mm Petri dishes (Mattek) and irradiated with 405 nm laser light for 5 min at the final power of 1 mW. Spheroids were cultured for a further 24 h postirradiation and, after this period, were processed for further staining with Hoechst 33258 (20 μg mL^–1^), calcein AM (2 μM), and PI (8 μg mL^–1^) for 2 h. Samples were imaged on a confocal microscope Leica CM SP5 (Leica, Germany) in 10 z-stack scans. Images were processed by using ImageJ software.

For detection of the localization in 3D culture of Hela cells, HeLa cells were seeded on 96w ultralow attachment U-shape plates (Corning) at the density of 5000 cells/well in the 3D forming medium: DMEM-F12 ham medium supplemented with growth and spheroid forming factors: 2% B27 (Thermo Fisher Scientific Inc., MA, USA), epidermal growth factor (EGF; Sigma-Aldrich, Germany, 20 ng mL^–1^), fibroblast growth factor (FGF2; Sigma-Aldrich, Germany, 10 ng mL^–1^), and bovine serum albumin (BSA) (Sigma-Aldrich, Germany, 0.15%). After 72 h of incubation, preformed spheroids were transferred as single spheres to Matrigel embed (30 min of embedding) and kept for 24 h in a 3D forming culture medium. Then, the spheroids were treated with tested compounds at the concentration of 2 μM for 5 h, and following that, the spheroids were washed and transferred to confocal 35 mm Petri dishes (Mattek). **Ir1** was excited with a 405 nm blue laser (1 mW), and the detection window was set from 500 to 550 nm. Samples were imaged on a confocal microscope Leica CM SP5 (Leica, Germany). Images were processed by using ImageJ software.

## Supporting Information Available

Synthetic schemes of intermediates **A** and **B**, nuclear magnetic resonance (NMR), mass spectrometry, and stability studies; photophysical properties, X-ray diffraction, and NADH photooxidation; and evaluation for singlet oxygen and hydroxyl radical generation in cell-free media and biological experiments (PDF)
Molecular formula strings and biological data (CSV)
Crystallographic data of **Ir3** (CIF)

## Supplementary Material

jm3c01978_si_002.csv

jm3c01978_si_003.cif

## Accession Codes

CCDC 2302438 contains the supplementary crystallographic data for this paper. These data can be obtained free of charge via www.ccdc.cam.ac.uk/data_request/cif, or by emailing data request@ccdc.cam.ac.uk, or by contacting The Cambridge Crystallographic Data Centre, 12 Union Road, Cambridge CB2 1EZ, UK; fax: + 44 1223 336033.

## Abbreviations Used

3D: three-dimensional
ACQ: aggregation-caused quenching
AIE: aggregation-induced emission
DCM: dichloromethane
DMEM: Dulbecco’s modified Eagle’s medium
DMF: dimethylformamide;
DMSO: dimethyl sulfoxide
DPBF: 1,3-diphenylbenzofuran
dppz: dipyridophenazine
EBSS: Earle’s balanced salt solution
EtOH: ethanol
HPF: 3′-*p*-(hydroxyphenyl)fluorescein
hTERT: human telomerase reverse transcriptase
IC_50_: concentration of the agent inhibiting cell growth by 50%
ICP-MS: inductively coupled plasma mass spectrometry
LDH: lactate dehydrogenase
LLCT: ligand-to-ligand charge transfer
MLCT: metal-to-ligand charge transfer
MS: mass spectrometry
MTT: 3-[4,5-dimethylthiazol-2-yl]-2,5 diphenyl tetrazolium bromide
NADH: nicotinamide adenine dinucleotide (NAD) + hydrogen (H)
NMIBC: nonmuscle invasive cancer
PAGE: polyacrylamide gel electrophoresis
PBS: phosphate buffered saline
PDT: photodynamic therapy
PI: propidium iodine
PMSF: phenylmethylsulfonyl fluoride
PS: phosphatidylserine
PSs: photosensitizers
ROS: reactive oxygen species
RP-HPLC: reversed-phase high performance liquid chromatography
RPMI: Roswell Park Memorial Institute
SD: standard deviation
SDS: sodium dodecyl sulfate
SEM: standard error of the mean
SRB: sulforhodamine B
TOF: turnover frequency
TON: turnover number
