# Supplementary material for: Novel 2-(5-Arylthiophen-2-yl)-benzoazole Cyclometalated Iridium(III) dppz Complexes Exhibit Selective Phototoxicity in Cancer Cells by Lysosomal Damage and Oncosis
Source: J Med Chem. 2023 Dec 23;67(1):691–708. doi: 10.1021/acs.jmedchem.3c01978 (PMC10788912; doi:10.1021/acs.jmedchem.3c01978)
Supplement: Supplementary file 1 — jm3c01978_si_001.pdf [file jm3c01978_si_001.pdf]

Supporting information

Novel 2-(5-arylthiophen-2-yl)-benzoazole cyclometalated iridium(III) dppz complexes exhibit selective phototoxicity in cancer cells by lysosomal damage and oncosis

Jana Kasparkova,[a] Alba Hernández-García,[b] Hana Kostrhunova,[a] Marta Goicuría,[b] Vojtech Novohradsky,[a] Delia Bautista,[c] Lenka Markova,[a] María Dolores Santana,[b] Viktor Brabec*,[a] and José Ruiz*,[b]

[a]*Czech Academy of Sciences, Institute of Biophysics, Kralovopolska 135, CZ-61200 Brno, Czech Republic*
[b]*Departamento de Química Inorgánica, Universidad de Murcia, and Biomedical Research Institute of Murcia (IMIB-Arrixaca), E-30100 Murcia, Spain*
[c]ACTI, *Universidad de Murcia, E-30100, Murcia, Spain*

Table of contents

1. Synthetic routes S1
2. Characterization of ligands S1
3. Characterization of complexes S10
4. Photophysical properties S25
5. Stability studies S27
6. Photo-oxidation of NADH and evaluation for $^1O_2$ and/or •OH generation in cell-free media.... S33
7. Biological assays S38

1. Synthetic routes

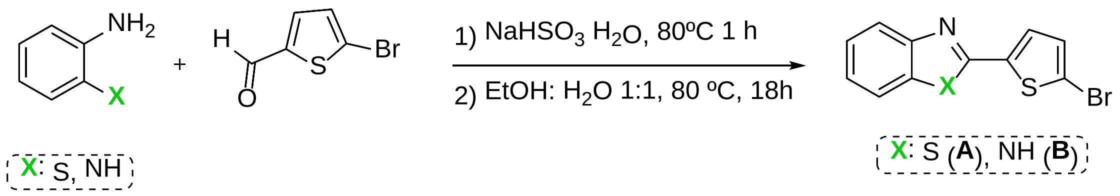

Scheme S1. Synthetic procedure for intermediates **A** and **B**

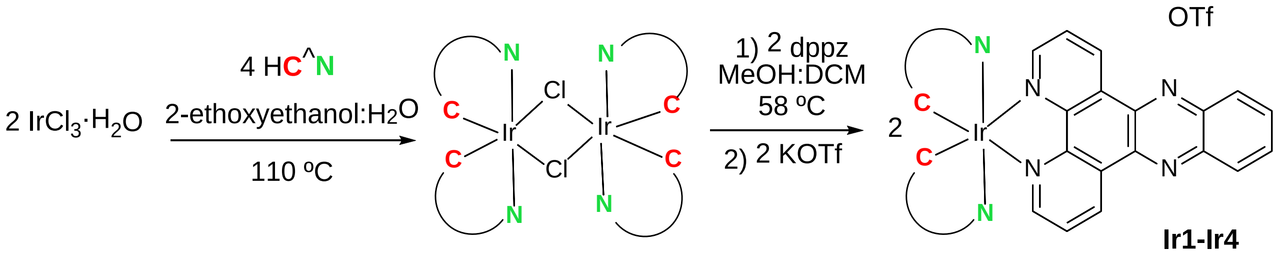

Scheme S2. Synthesis of iridium complexes **Ir1–Ir4** investigated.

2. Characterization of ligands

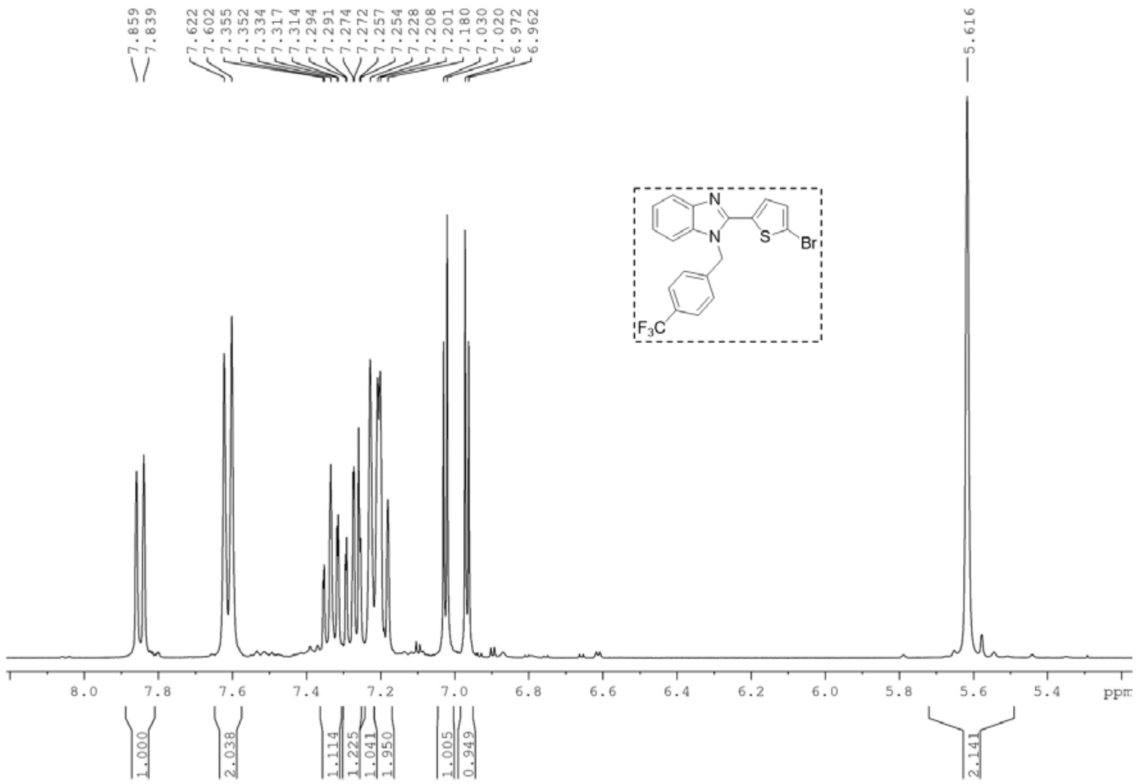

Figure S1. ^{1}H NMR spectrum of **B1** in $CDCl_3$.

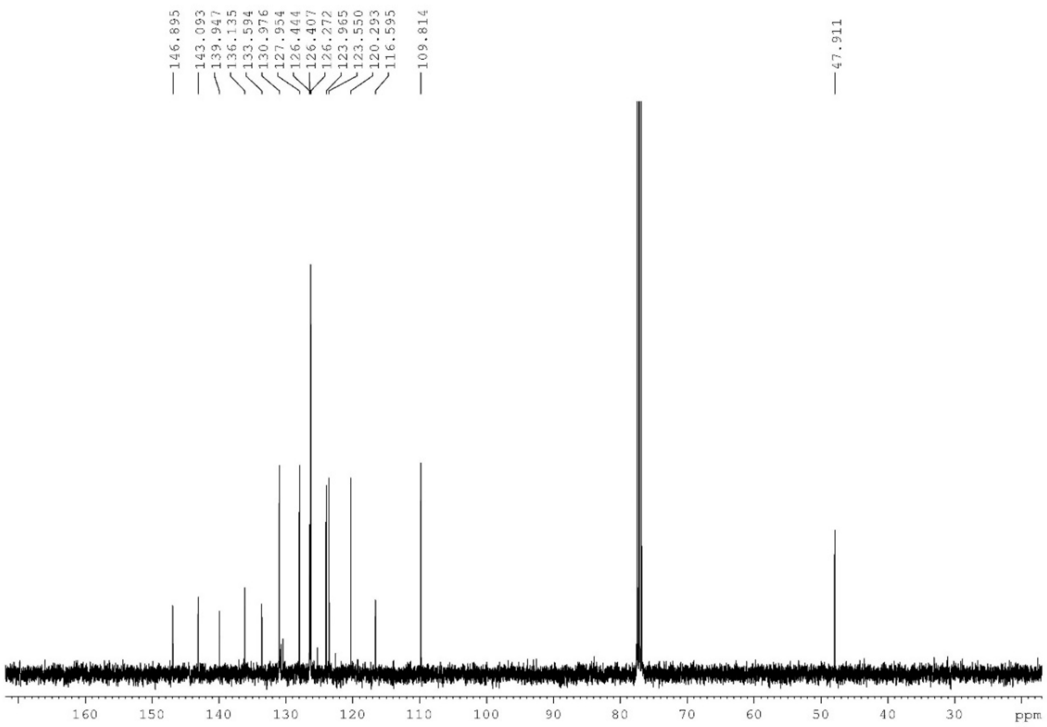

Figure S2. ^{13}C NMR spectrum of **B1** in $CDCl_3$.

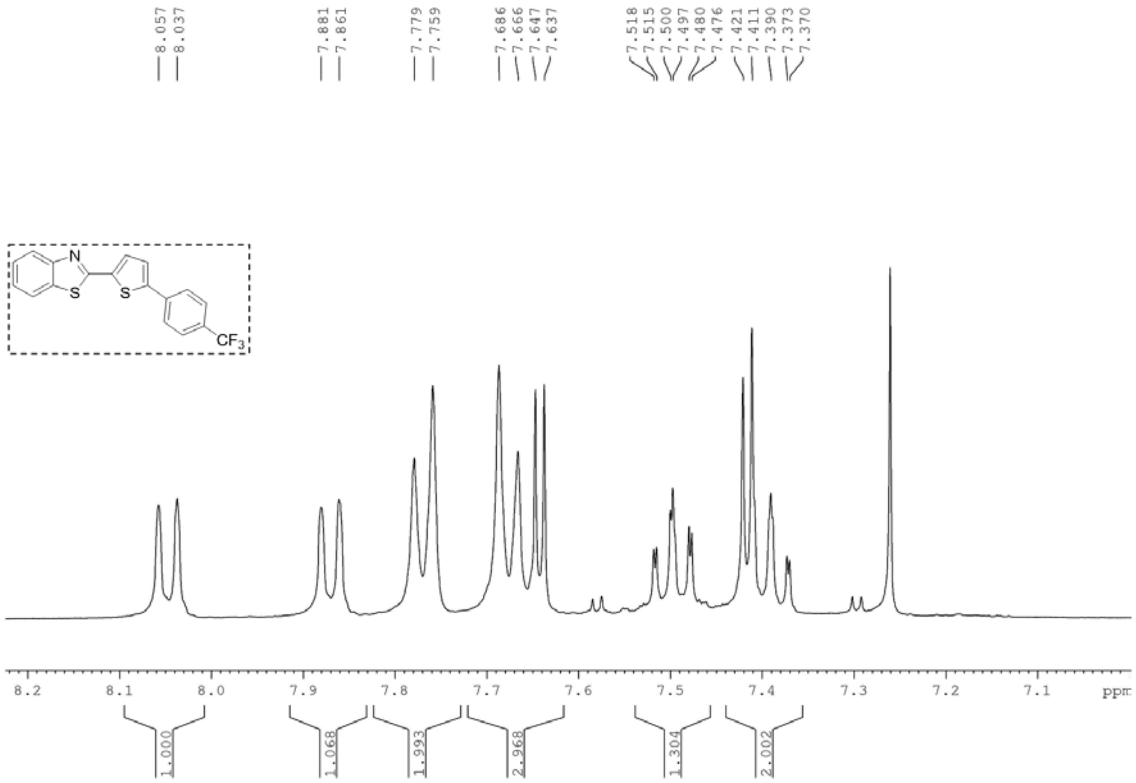

Figure S3. ^{1}H NMR spectrum of **HL1** in $CDCl_3$.

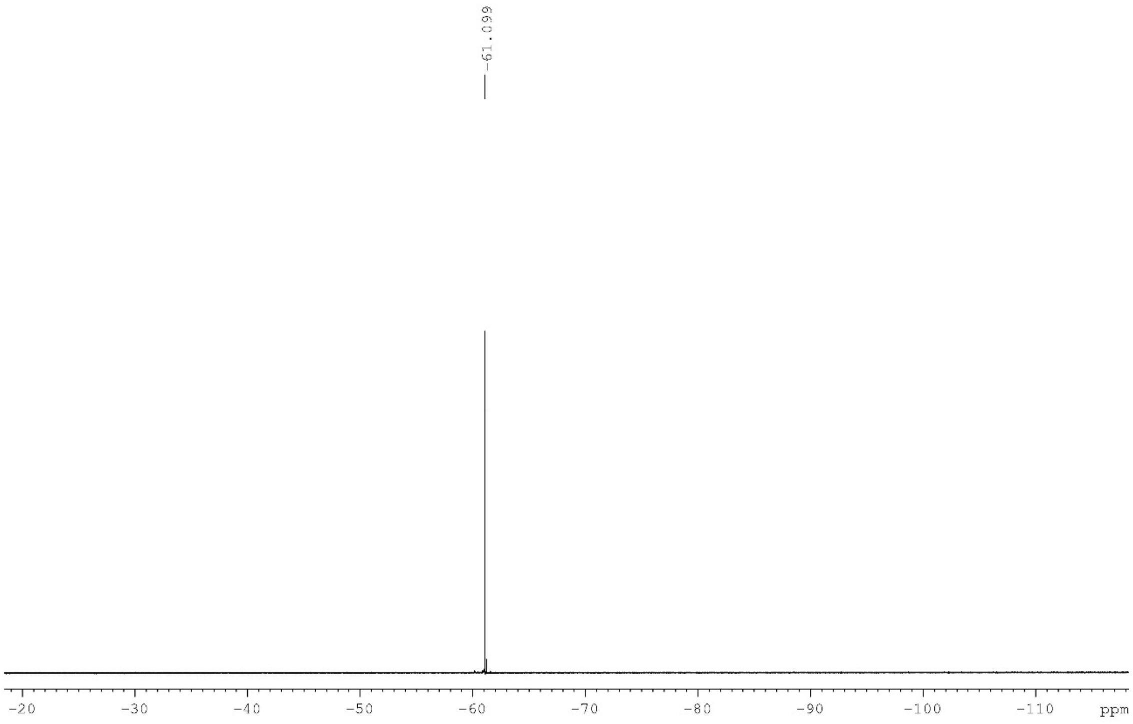

Figure S4. ^{19}F NMR spectrum of **HL1** in DMSO.

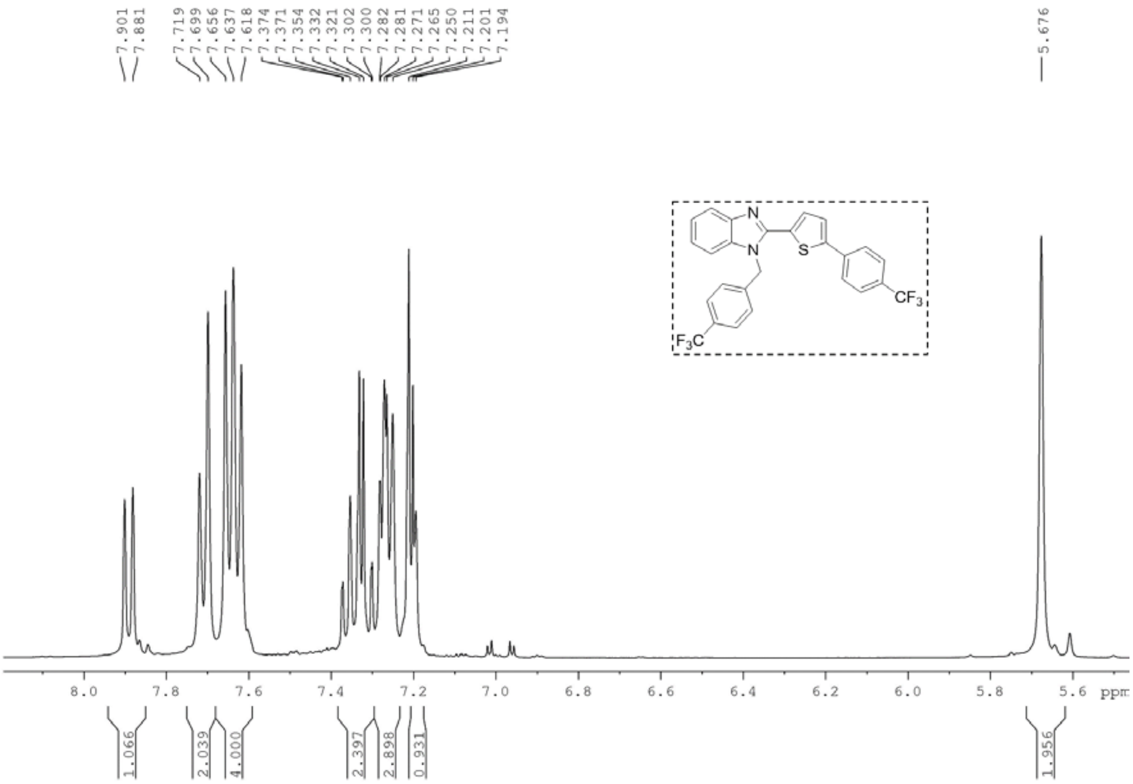

Figure S5. ^{1}H NMR spectrum of **HL3** in $CDCl_3$.

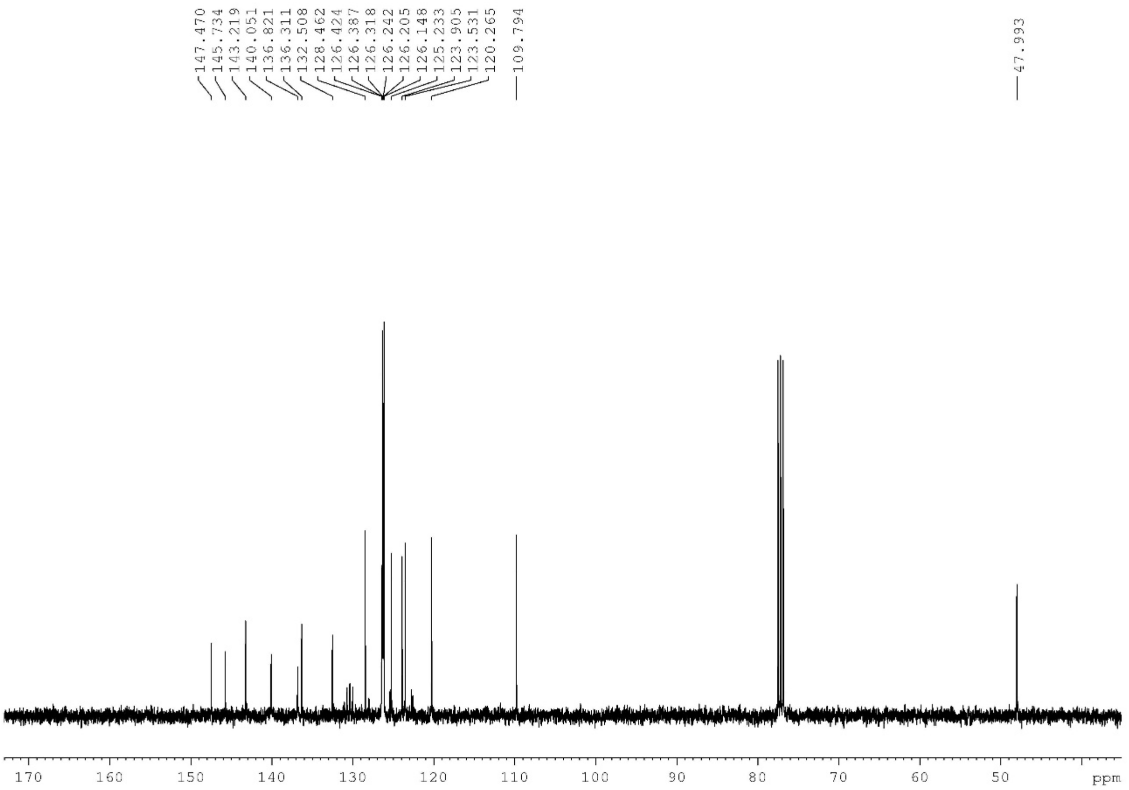

Figure S6. ^{13}C NMR spectrum of **HL3** in $CDCl_3$.

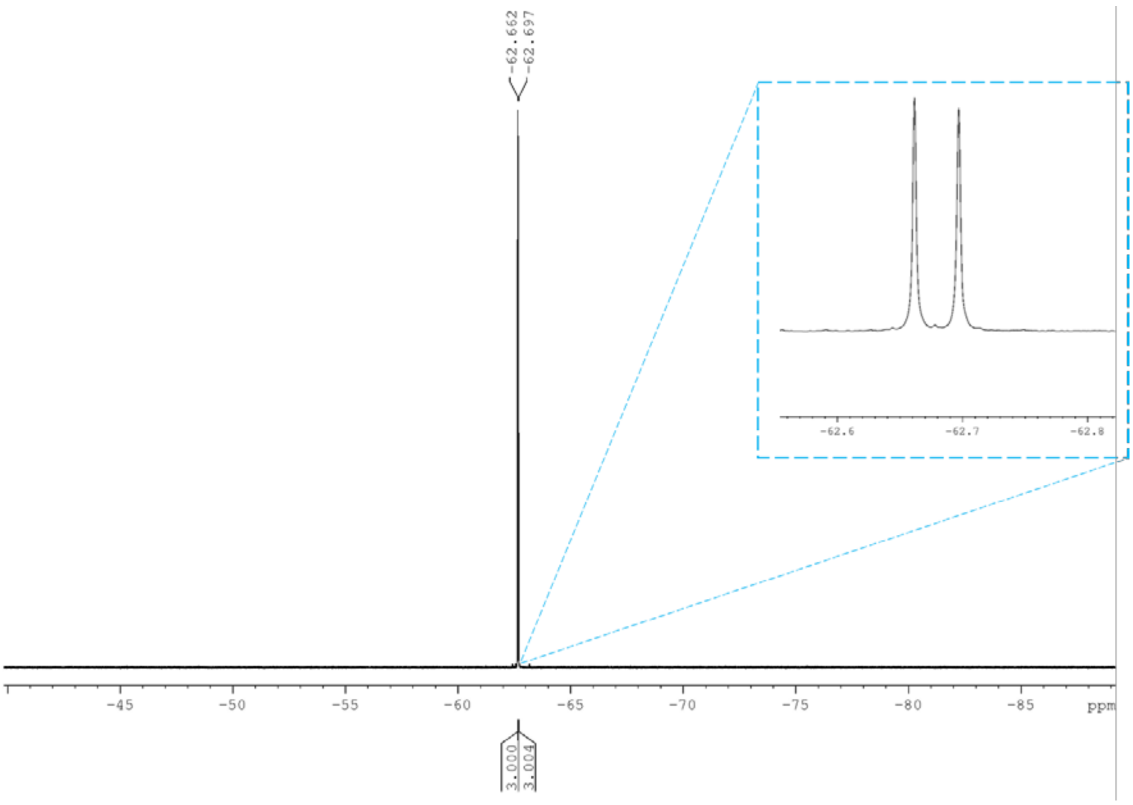

Figure S7. ^{19}F NMR spectrum of **HL3** in DCM.

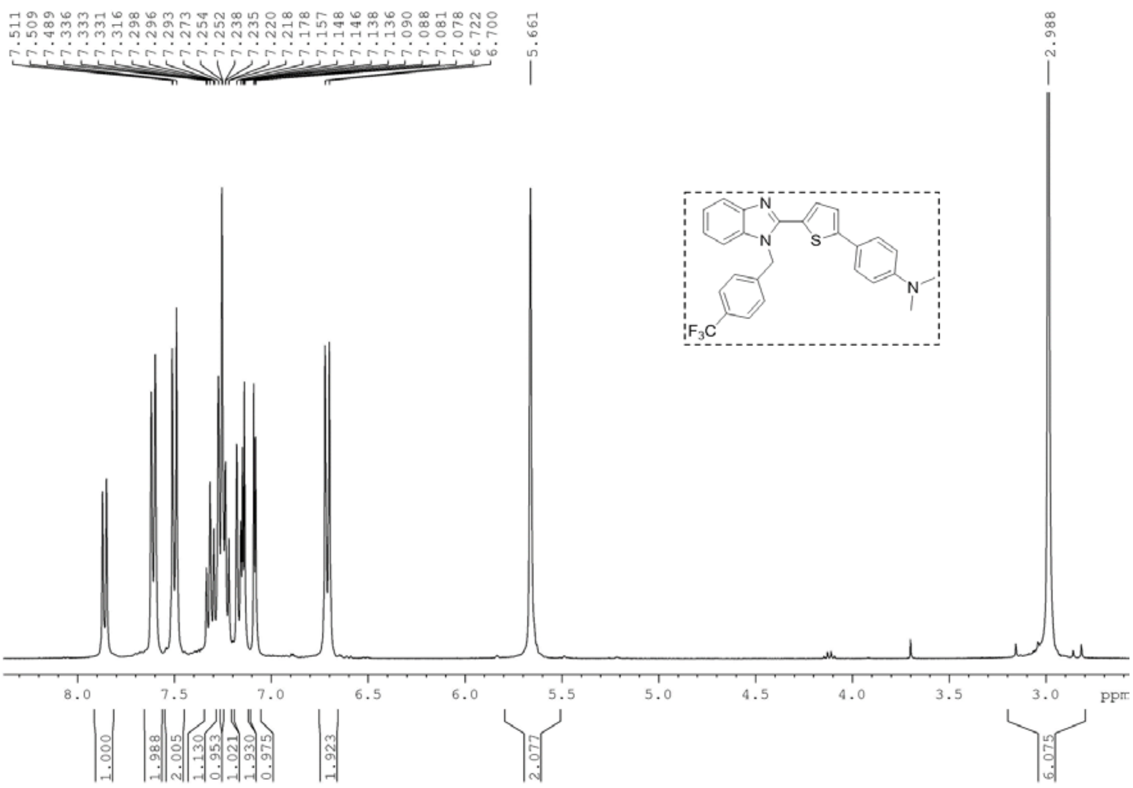

Figure S8. ^{1}H NMR spectrum of **HL4** in $CDCl_3$.

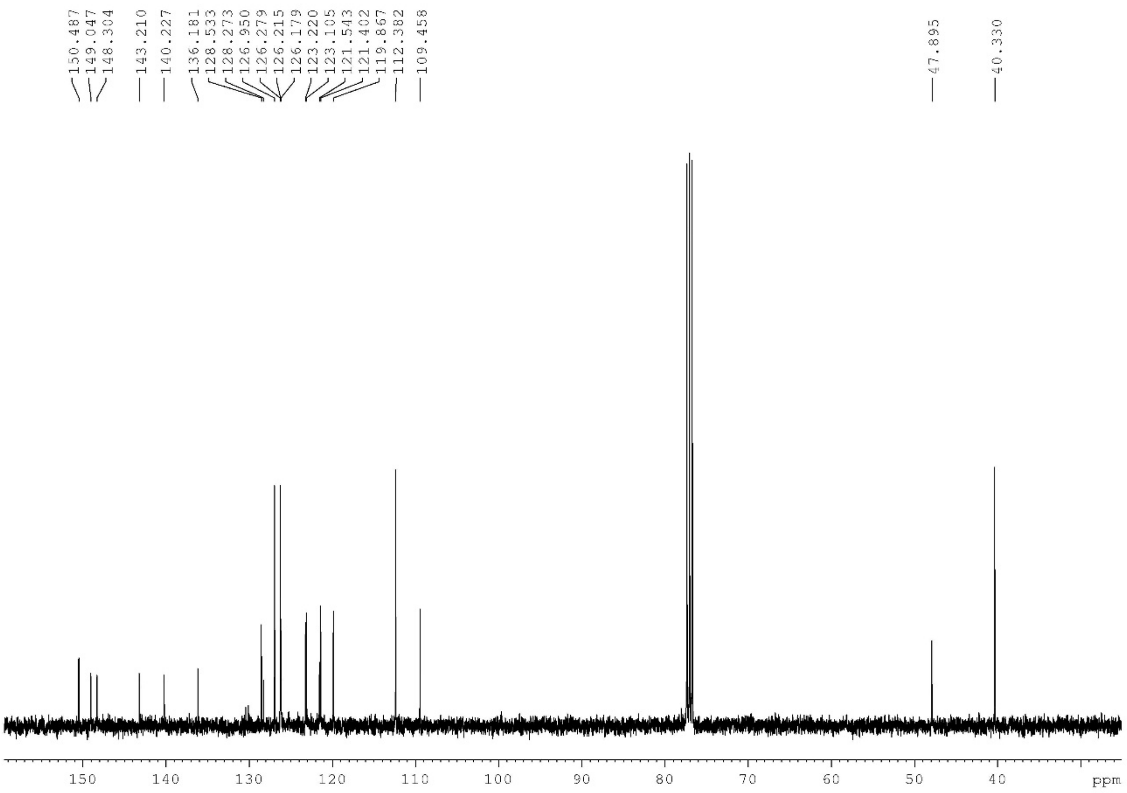

Figure S9. ^{13}C NMR spectrum of **HL4** in $CDCl_3$.

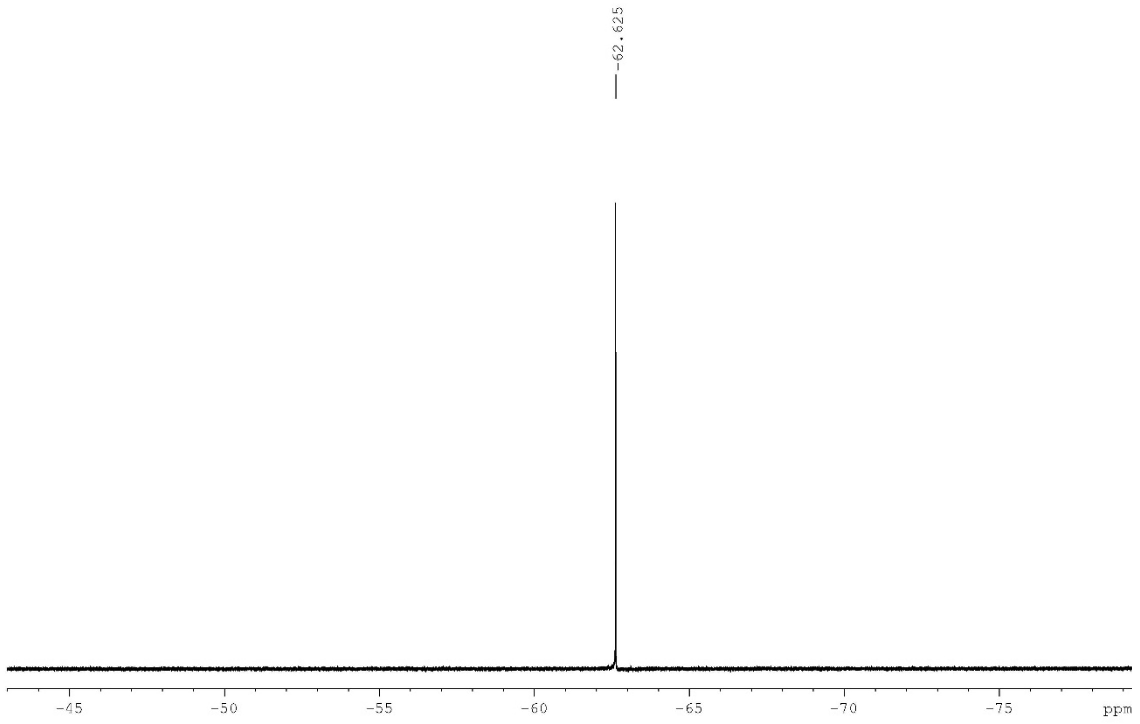

Figure S10. ^{19}F NMR spectrum of **HL4** in $CDCl_3$.

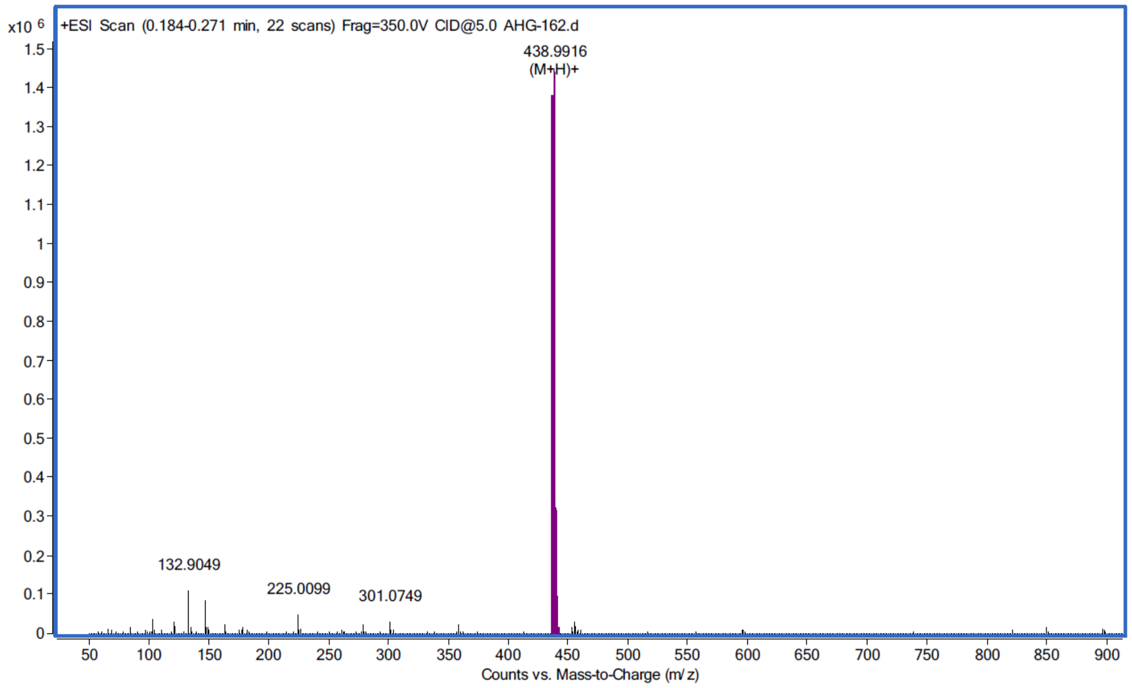

Figure S11. ESI-MS spectrum of **B1** (positive detection mode).

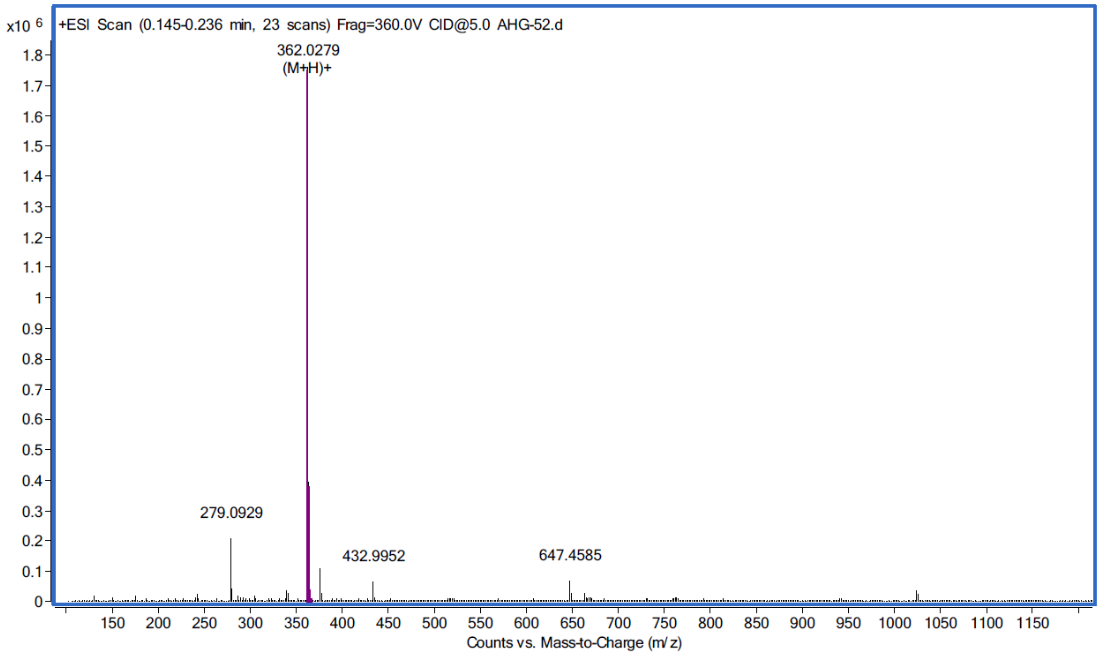

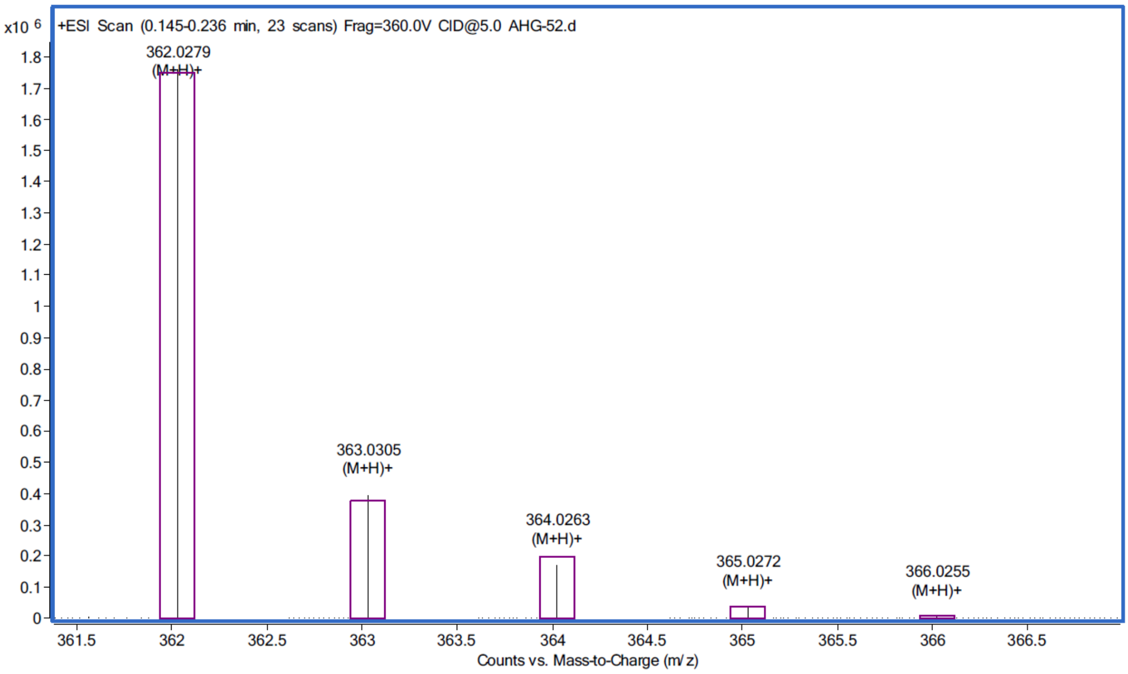

Figure S12. ESI-MS spectrum of complex **HL1** (positive detection mode).

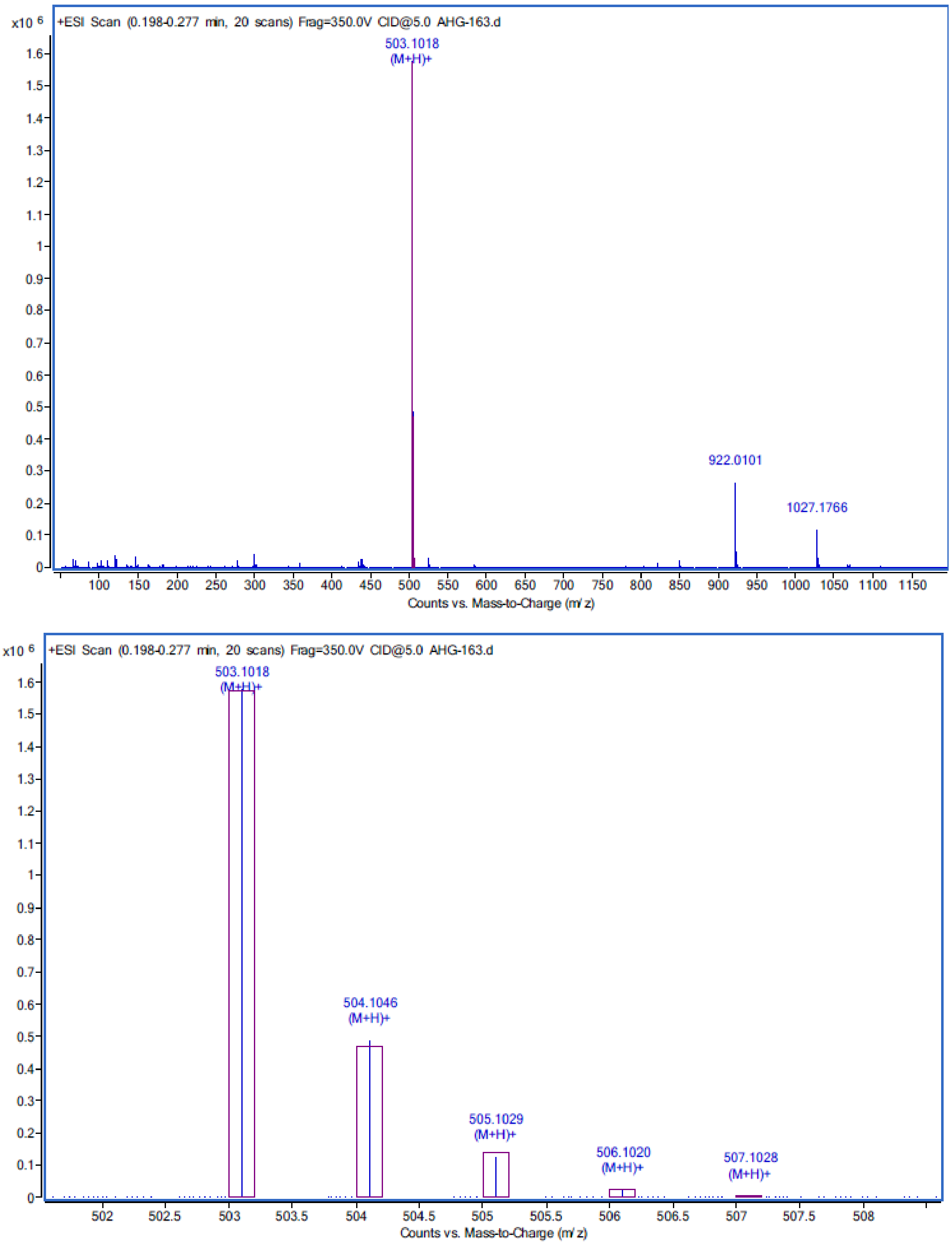

Figure S13. ESI-HRMS spectrum of complex **HL3** (positive detection mode).

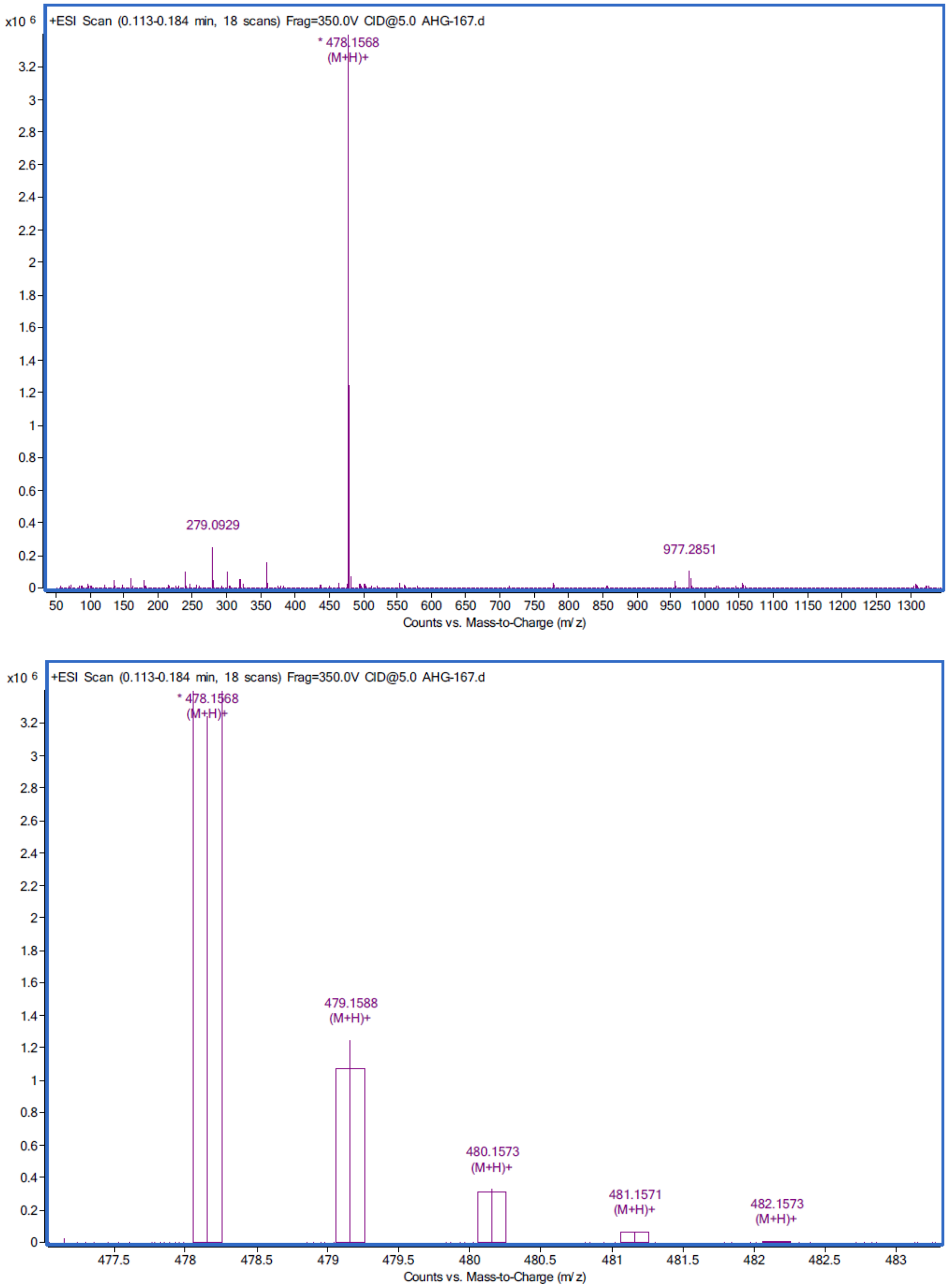

Figure S14. ESI-HRMS spectrum of complex **HL4** (positive detection mode).

3. Characterization of complexes

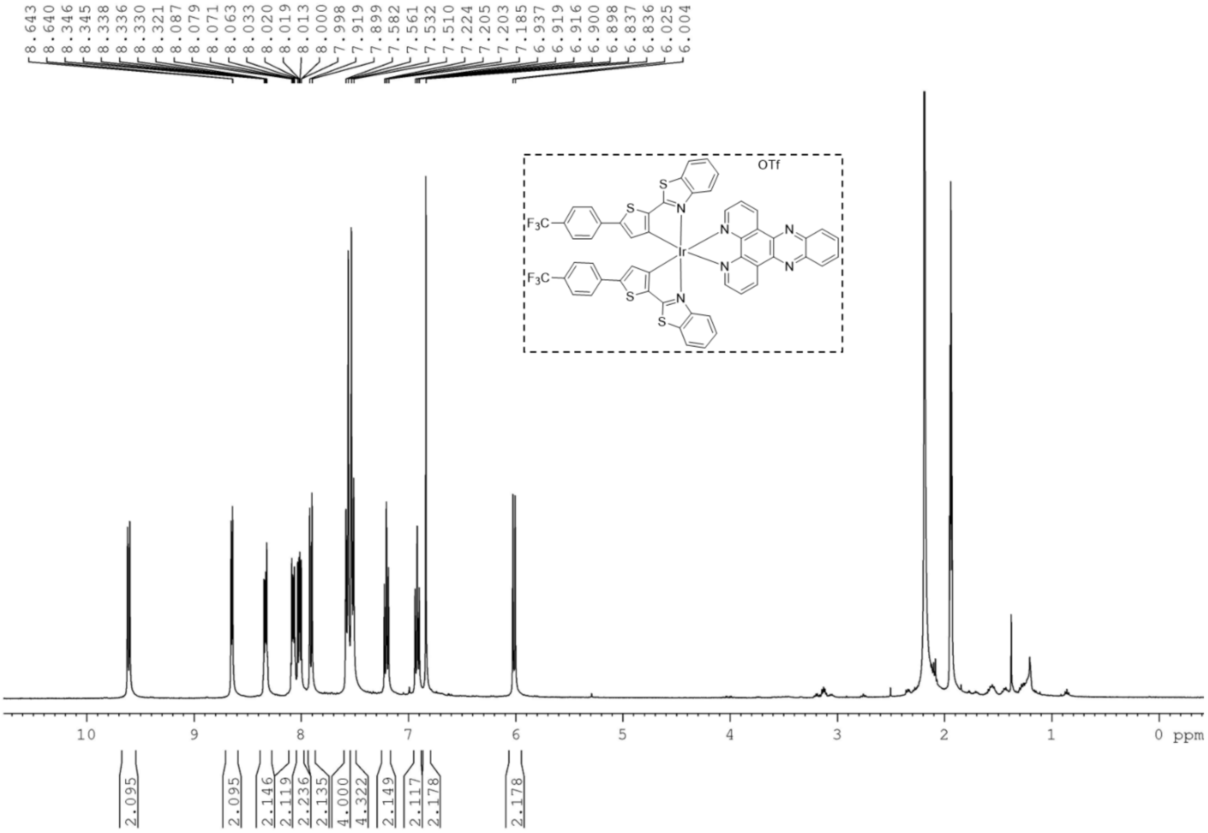

Figure S15. ^{1}H NMR spectrum of **Ir1** in $CDCl_3$.

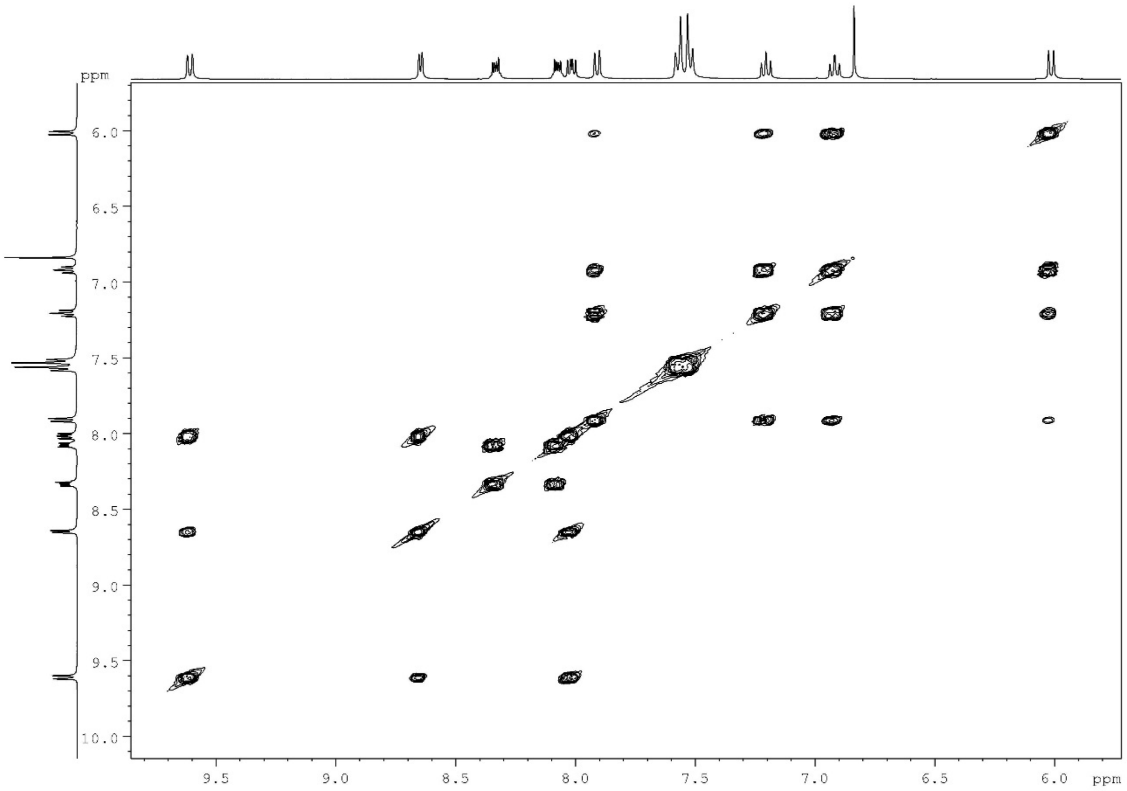

Figure S16. ^{1}H-^{1}H COSY NMR spectrum of **Ir1**.

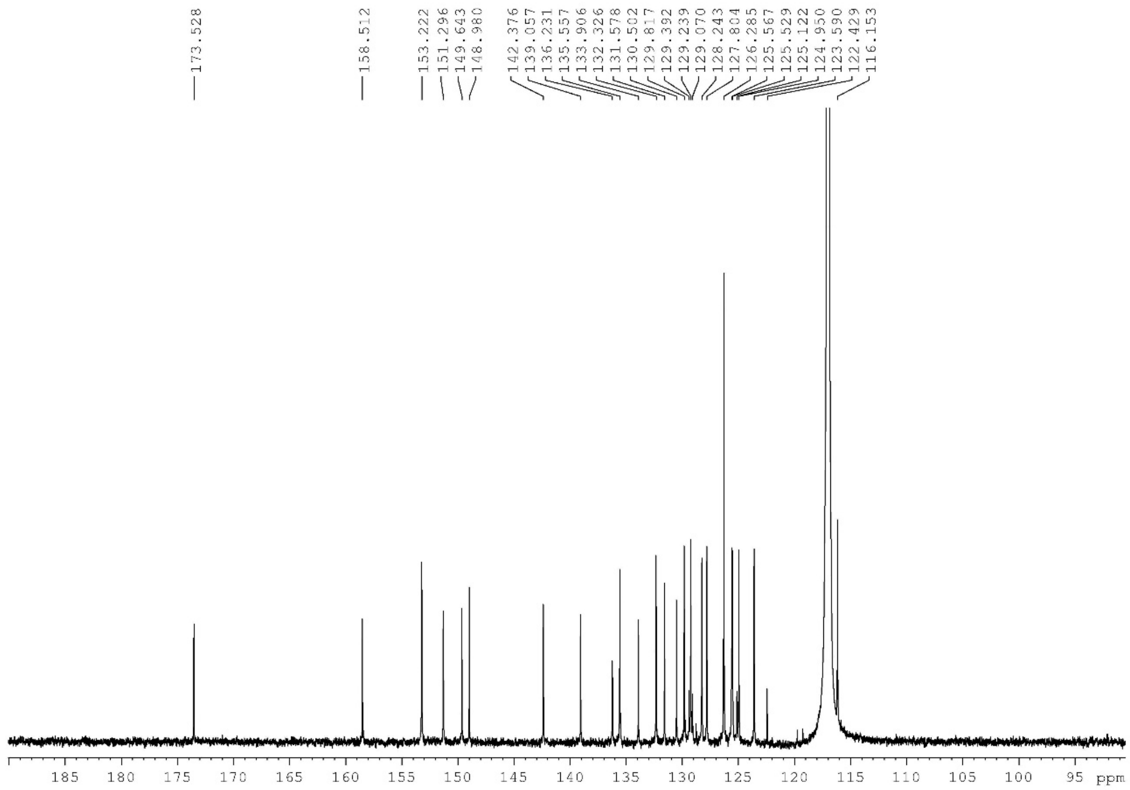

Figure S17. ^{13}C NMR spectrum of **Ir1** in $CDCl_3$.

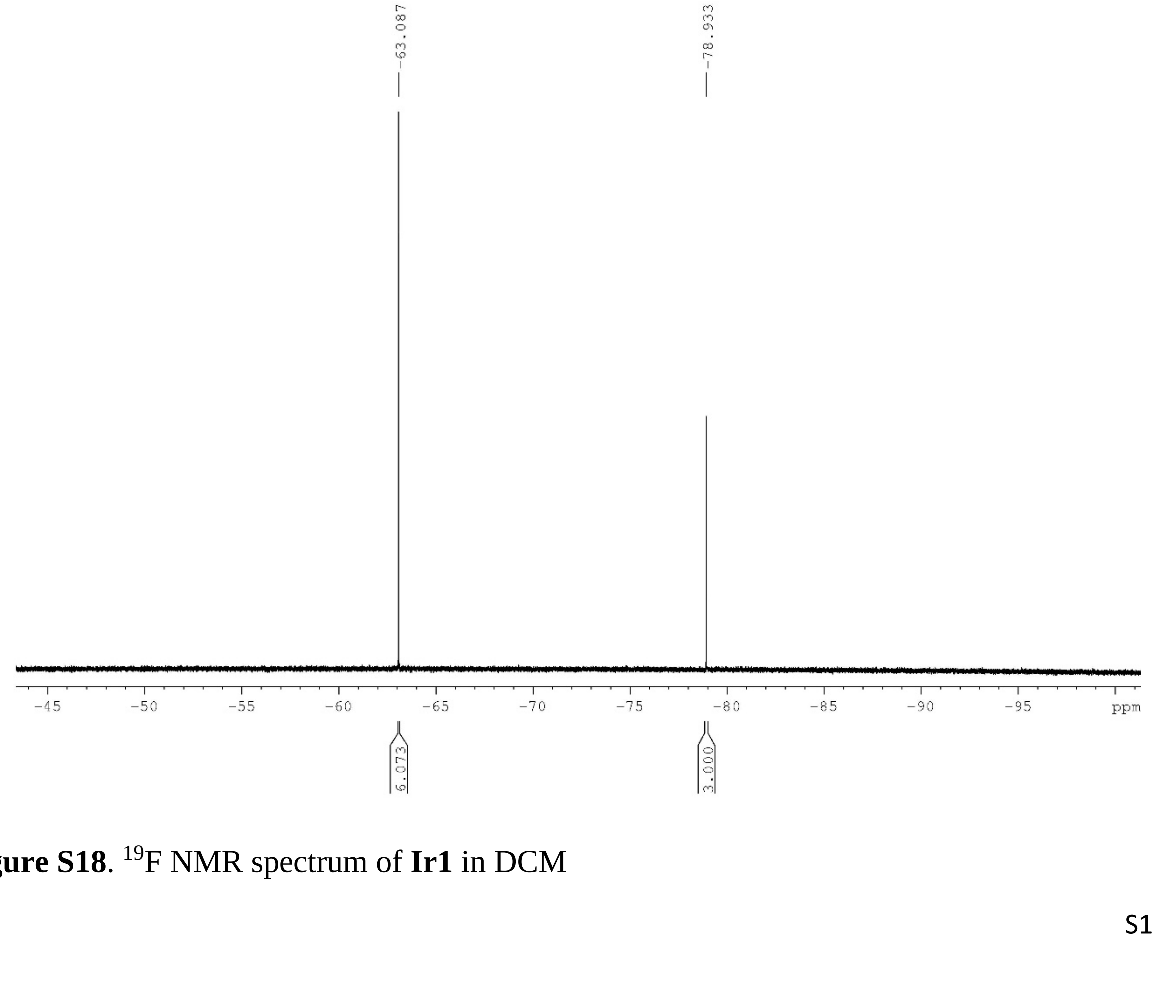

Figure S18. ^{19}F NMR spectrum of **Ir1** in DCM

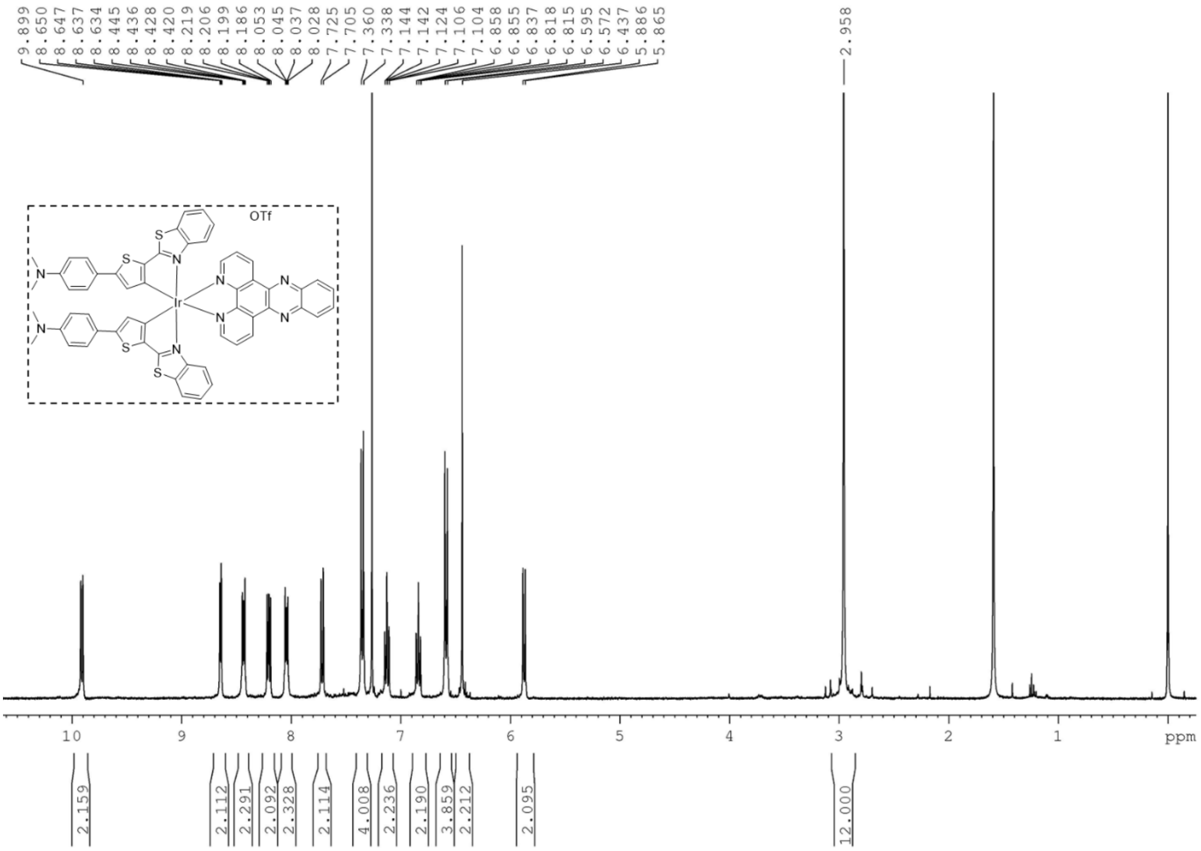

Figure S19. ^{1}H NMR spectrum of **Ir2** in $CDCl_3$.

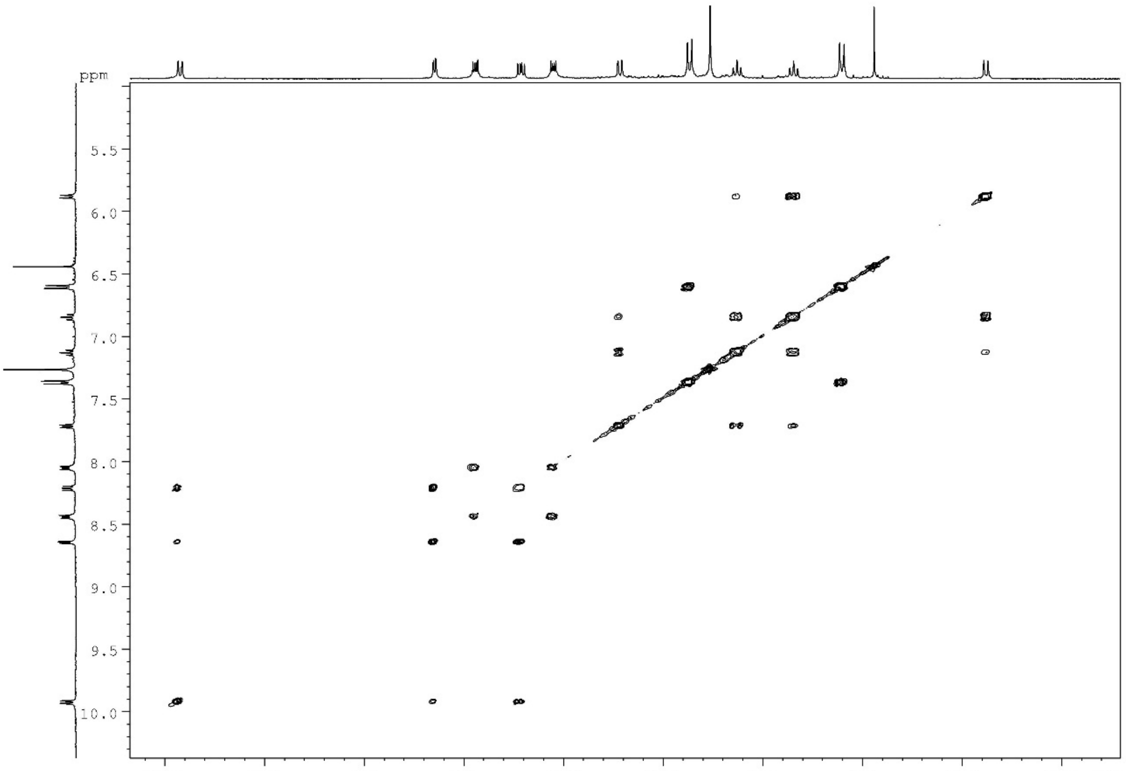

Figure S20. ^{1}H-^{1}H COSY NMR spectrum of **Ir2**.

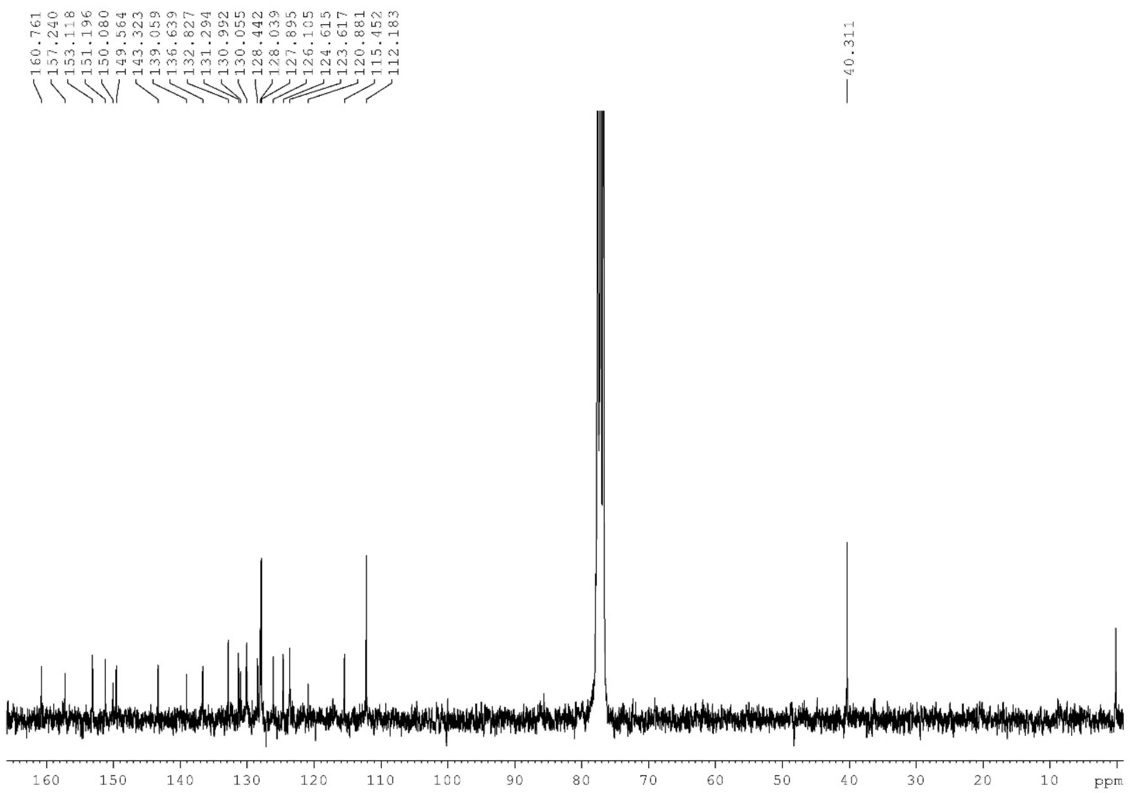

Figure S21. ^{13}C NMR spectrum of **Ir2** in $CDCl_3$.

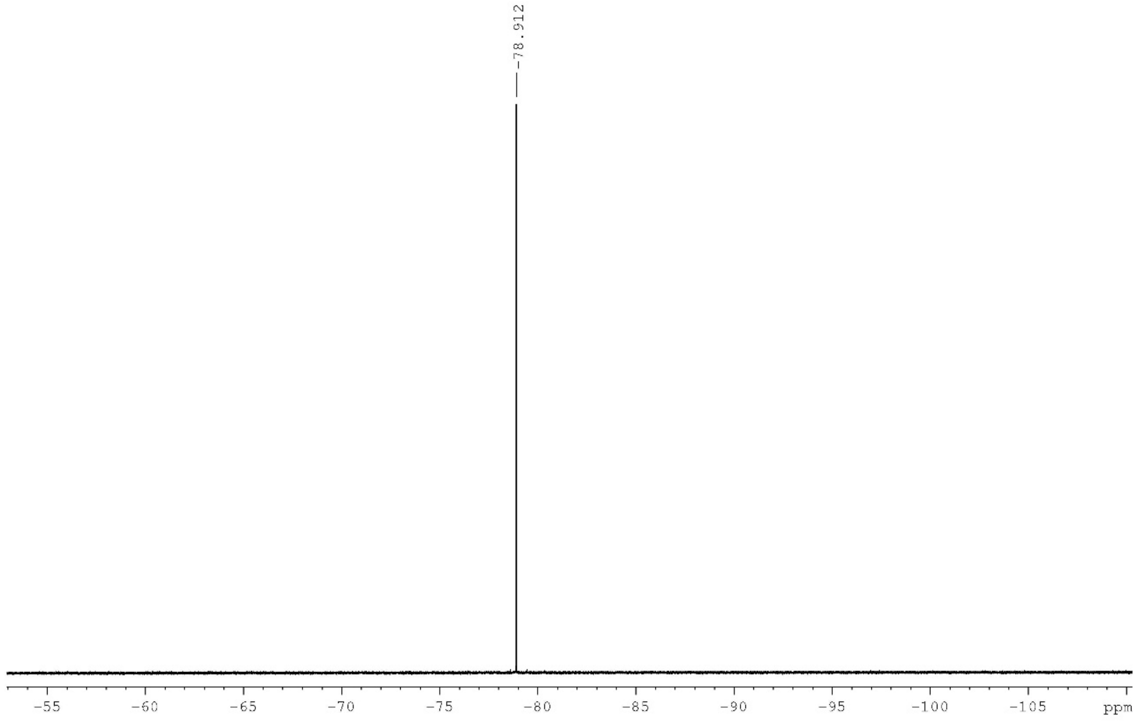

Figure S22. ^{19}F NMR spectrum of **Ir2** in DCM.

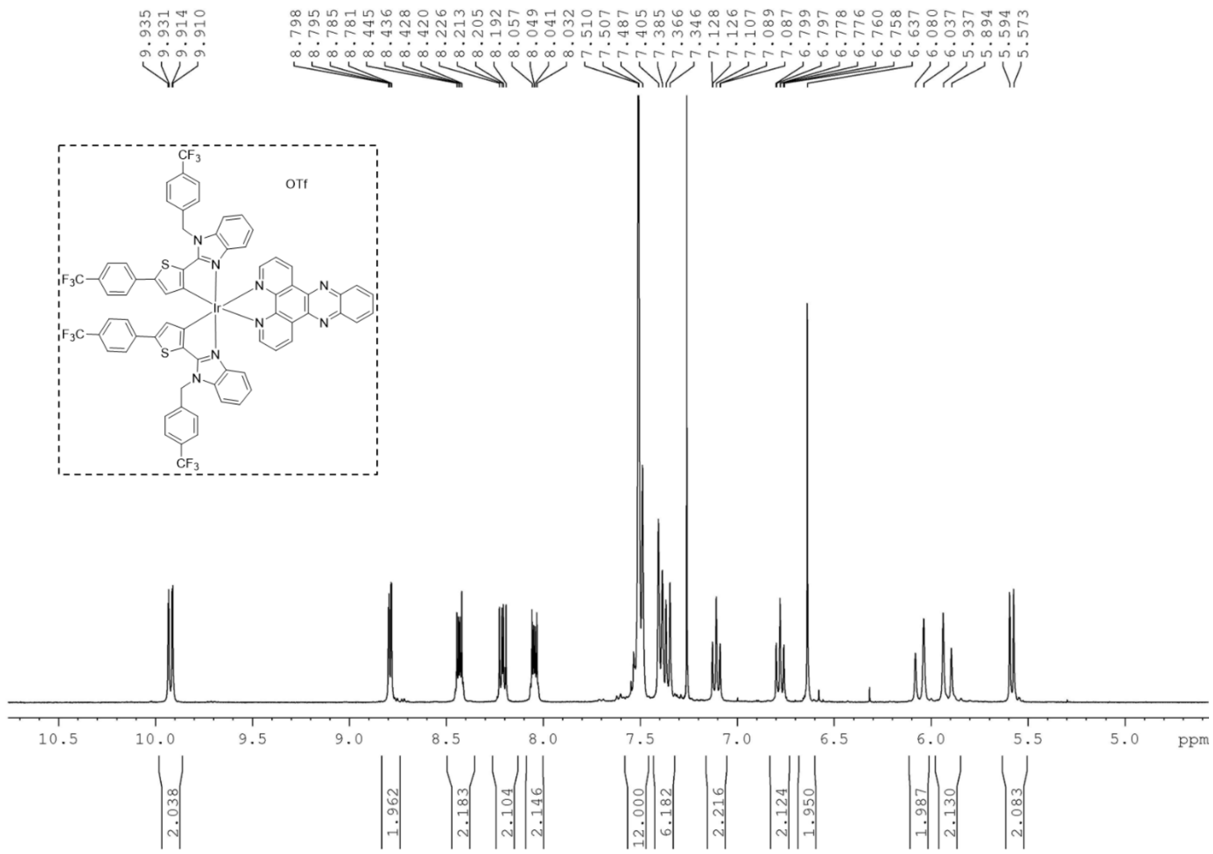

Figure S23. ^{1}H NMR spectrum of **Ir3** in $CDCl_3$.

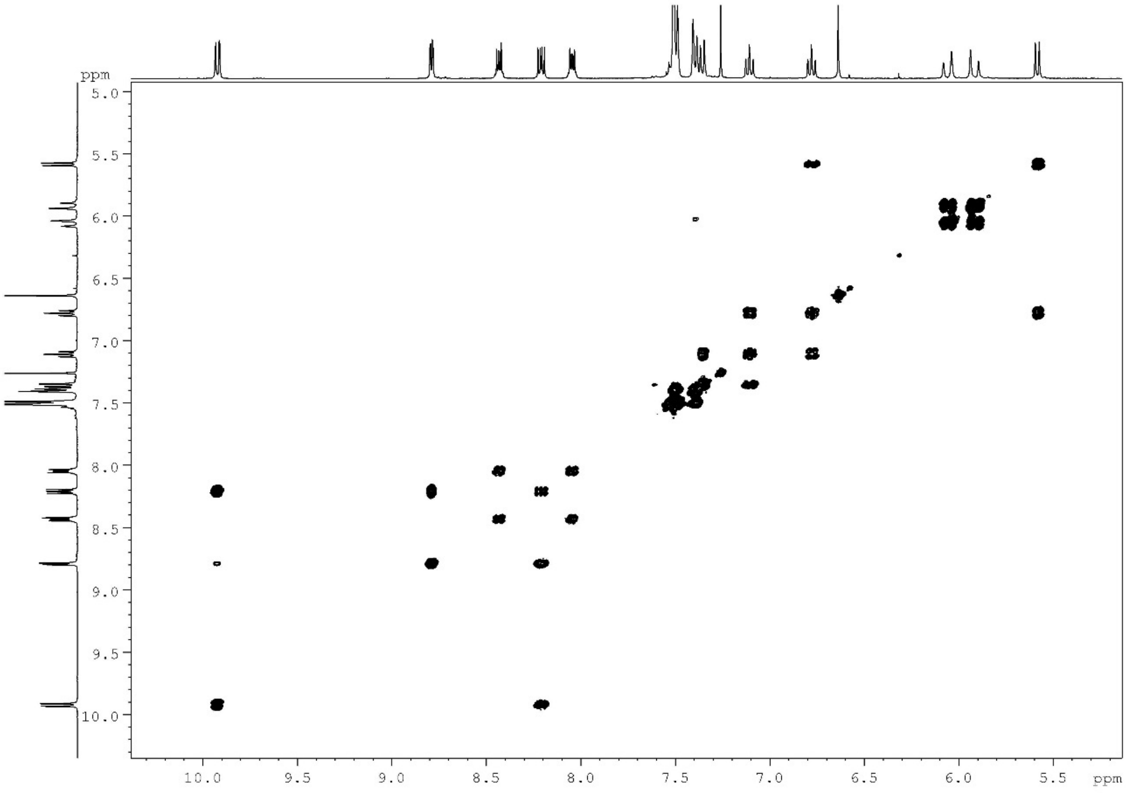

Figure S24. ^{1}H-^{1}H COSY NMR spectrum of **Ir3**

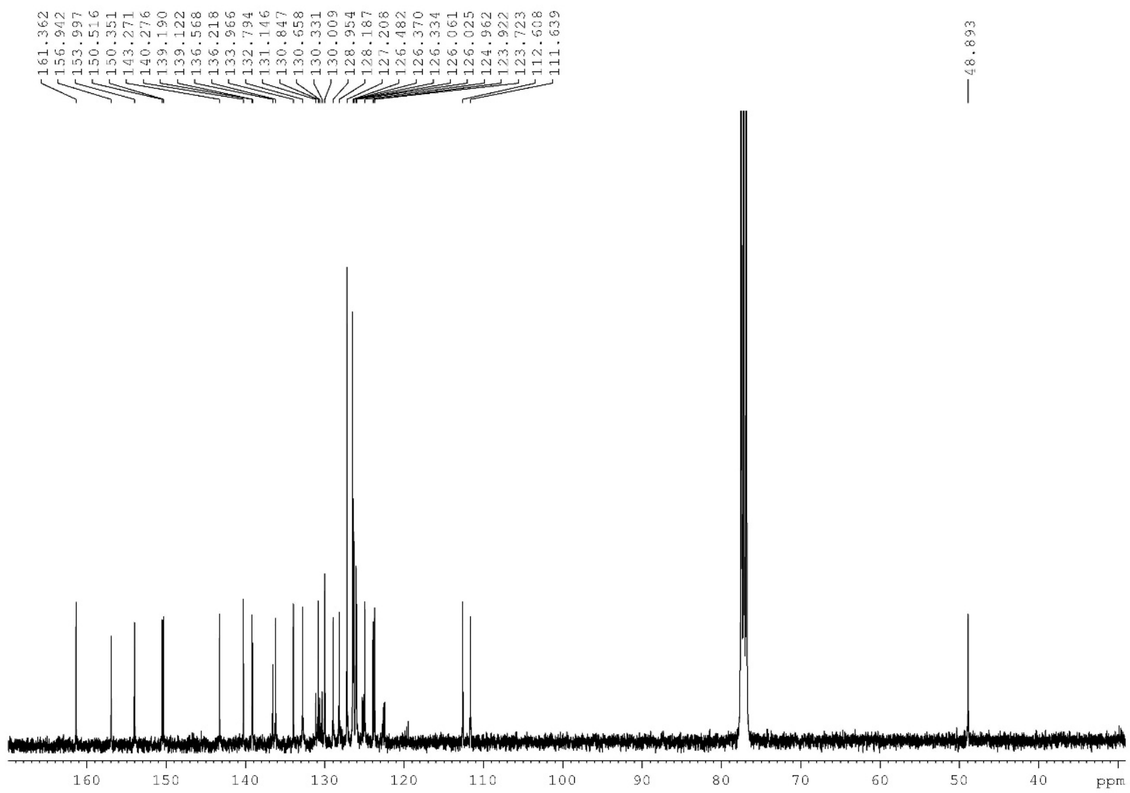

Figure S25. ^{13}C NMR spectrum of **Ir3** in $CDCl_3$.

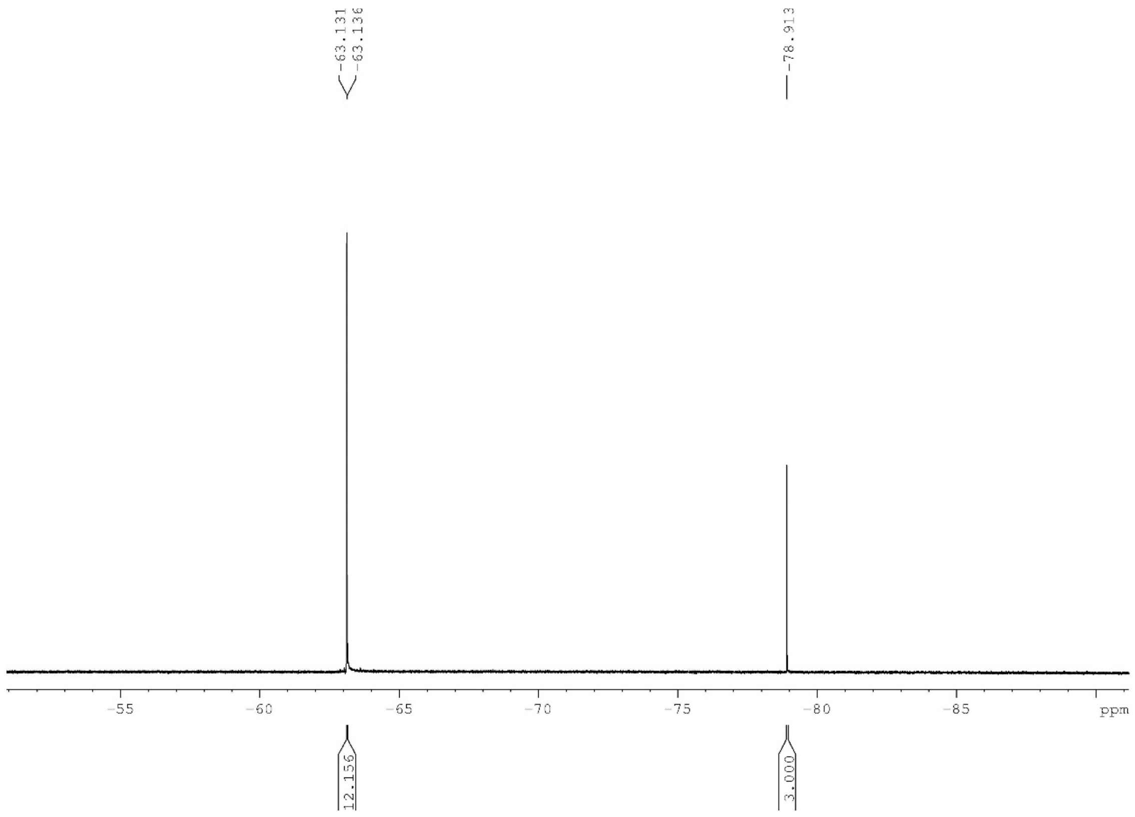

Figure S26. ^{19}F NMR (377 MHz) spectrum of **Ir3** in DCM.

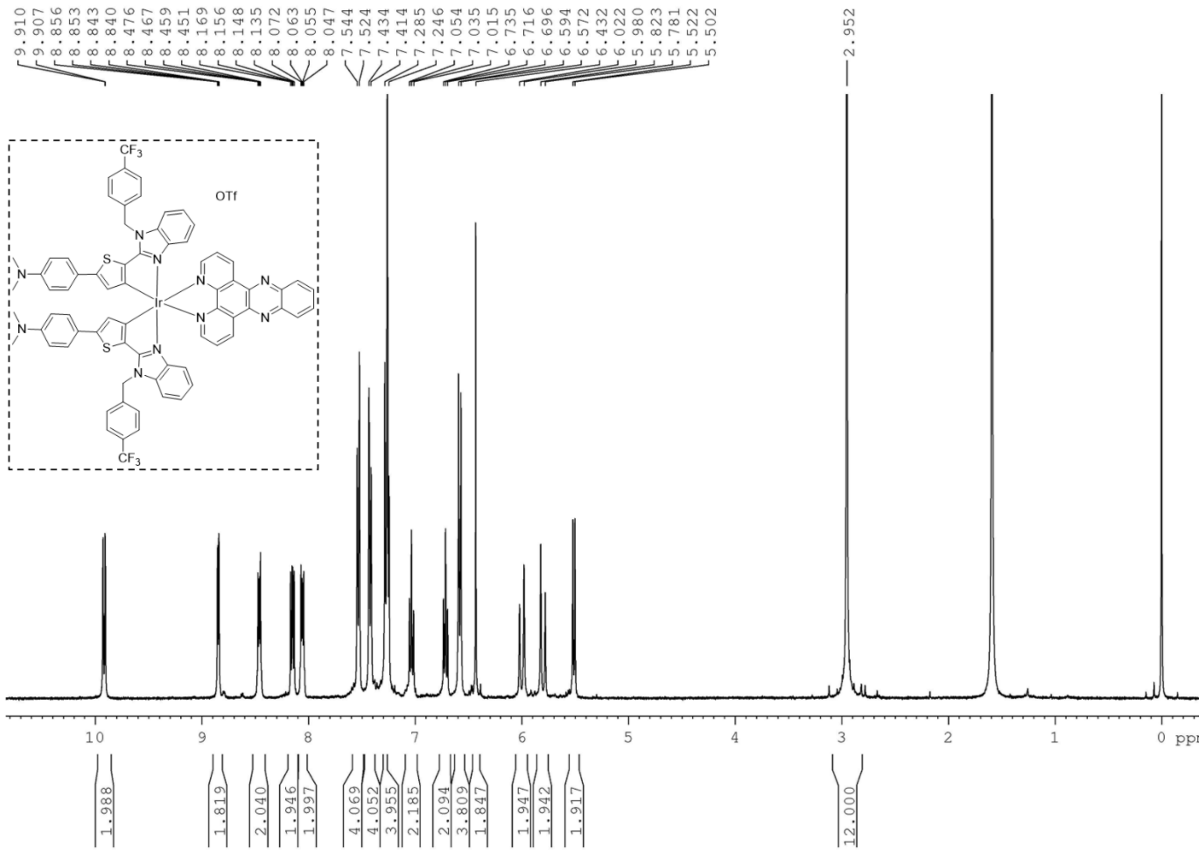

Figure S27. ^{1}H NMR spectrum of **Ir4** in $CDCl_3$.

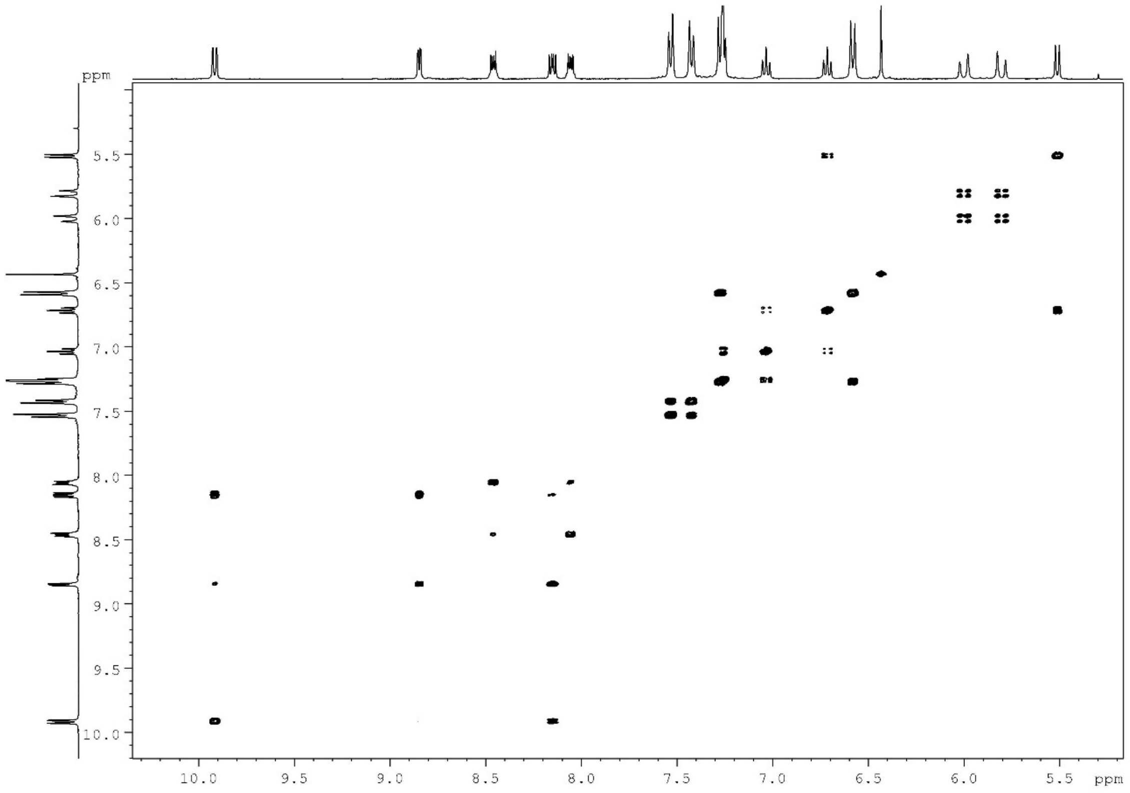

Figure S28. ^{1}H-^{1}H COSY NMR spectrum of **Ir4**.

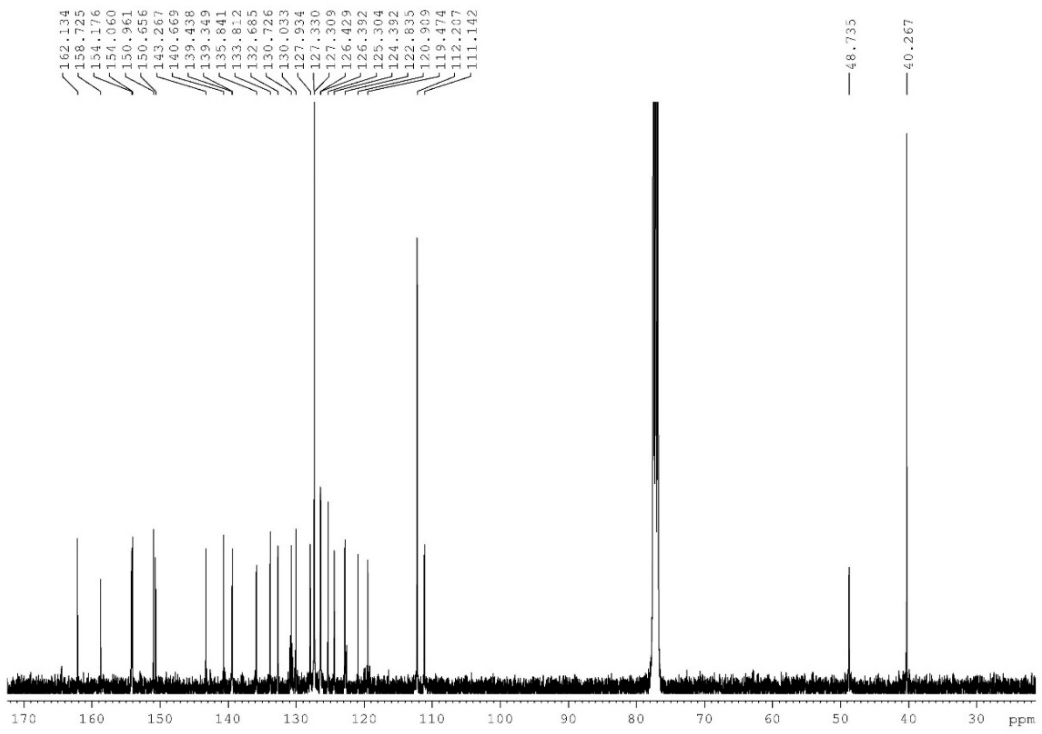

Figure S29. ${}^{13}C$ NMR spectrum of **Ir4** in $CDCl_3$.

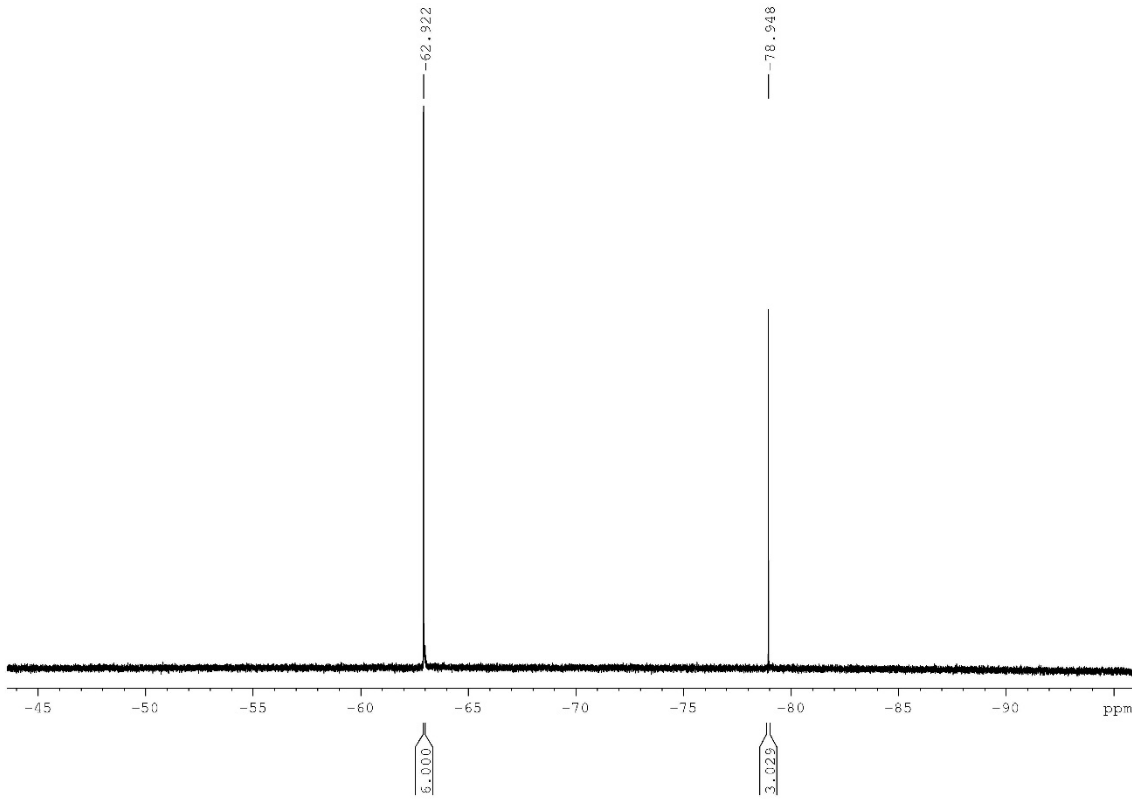

Figure S30. ${}^{19}F$ NMR spectrum of **Ir4** in DCM

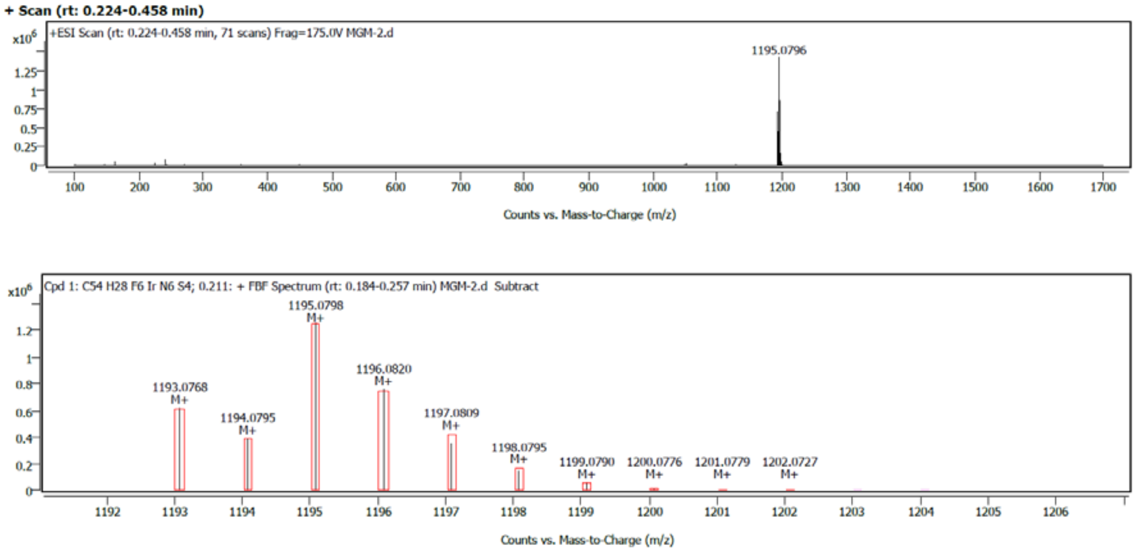

Figure S31. HR-MS spectrum of **Ir1** (positive detection mode).

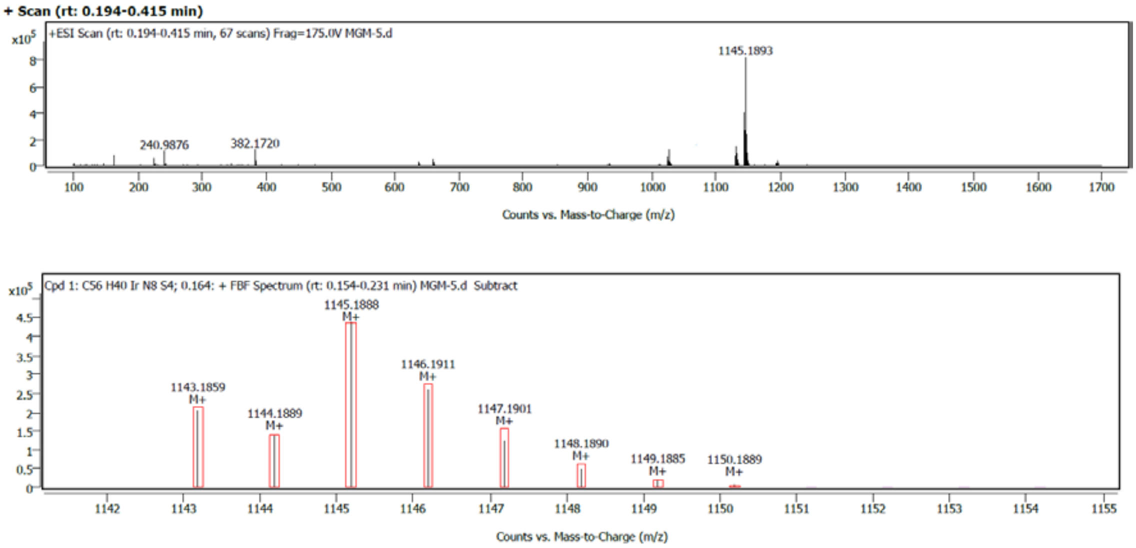

Figure S32. HR-MS spectrum of **Ir2** (positive detection mode).

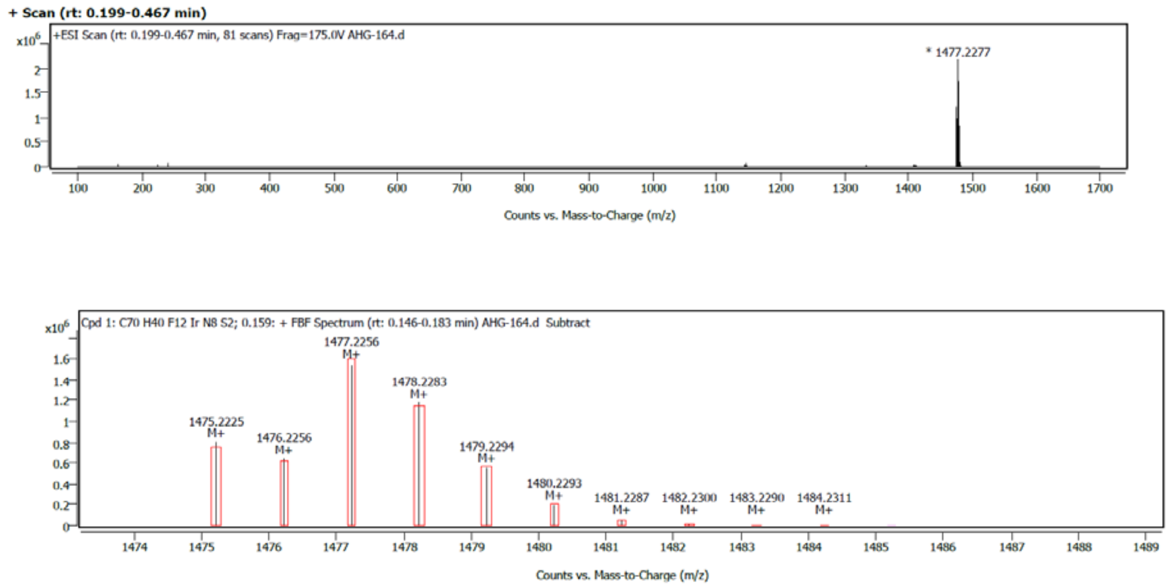

Figure S33. HR-MS spectrum of **Ir3** (positive detection mode).

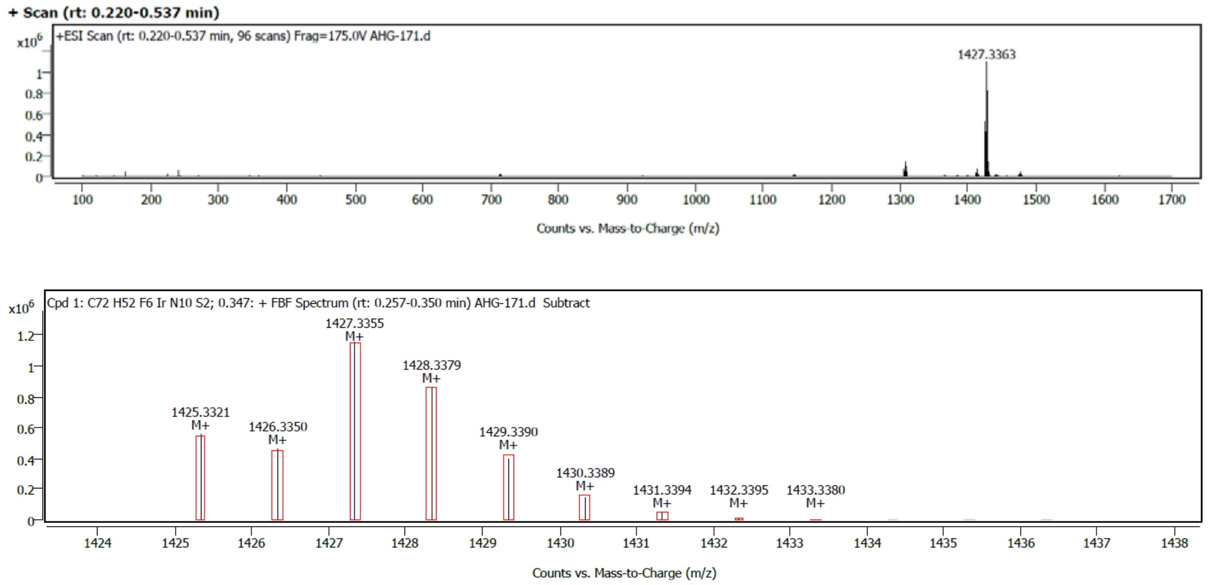

Figure S34. HR-MS spectrum of **Ir4** (positive detection mode).

Table S1. HPLC method

Time (min)	0.1 % formic acid in H_2O	0.1 % formic acid in CH_3CN	Flow (mL/min)
**0**	80	20	0.4
**25**	0	100	

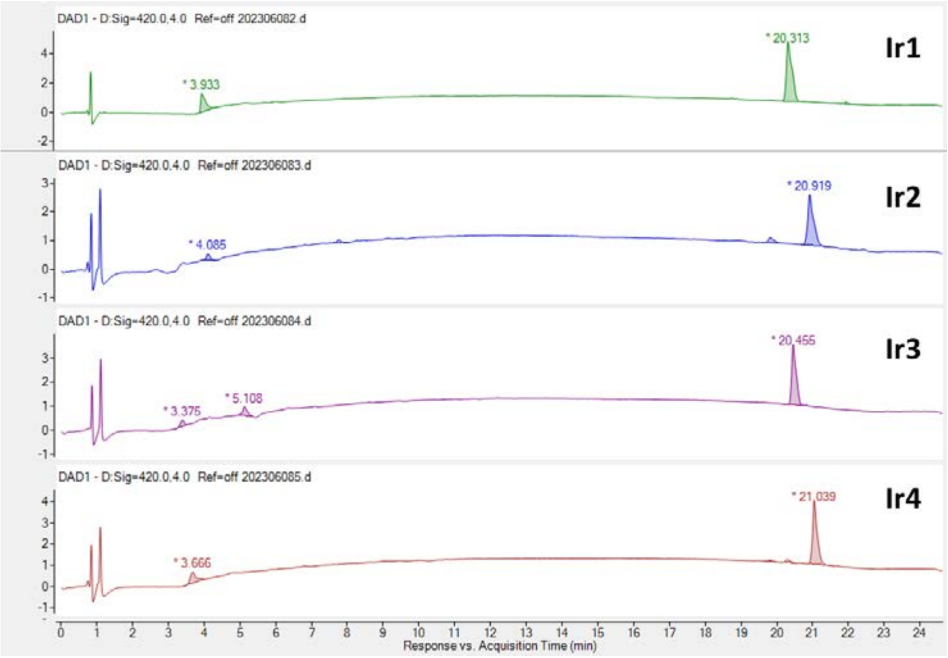

Figure S35. HPLC chromatograms with UV detection at 420 nm of complexes **Ir1-Ir4**.

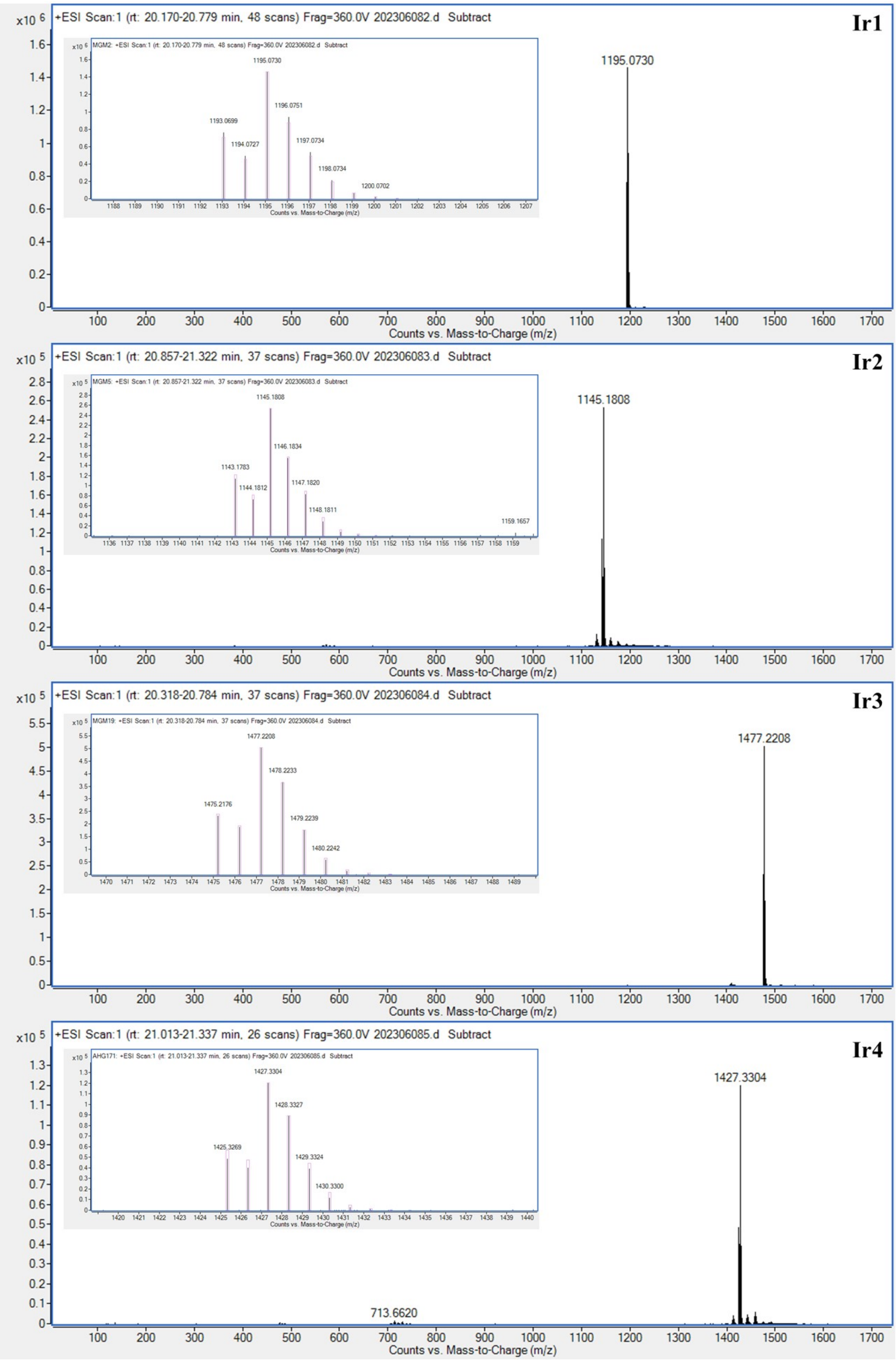

Figure S36. Mass spectra of the 20-21 min peak of chromatograms of **Figure S35** corresponding to the complexes **Ir1-Ir4**.

Table S2. X-ray diffraction data of complex **Ir3**

Empirical formula	C71 H40 F15 Ir N8 O3 S3	
Formula weight	1626,49	
Temperature	100(2) K	
Wavelength	0,71073 Å	
Crystal system	Triclinic	
Space group	P-1	
Unit cell dimensions	a = 14,302(7) Å	α = 78,870(14)°
	b = 15,809(7) Å	β = 66,395(15)°
	c = 17,023(7) Å	γ = 68,46(2)°
Volume	3276(3) Å^3	
Z	2	
Density (calculated)	1,649 Mg/m^3	
Absorption coefficient	2,233 mm^{-1}	
F(000)	1612	
Crystal size	0,125 x 0,080 x 0,020 mm^3	
Theta range for data collection	1,642 to 26,372°	
Index ranges	-17<=h<=17, -19<=k<=19, -21<=l<=21	
Reflections collected	193595	
Independent reflections	13398 [R(int) = 0,0508]	
Completeness to theta = 25.242°	100.0 %	
Absorption correction	Semi-empirical from equivalents	
Max. And min. transmission	0,7457 and 0,6483	
Refinement method	Full-matrix least-squares F^2	
Data / restraints / parameters	13398 / 1139 / 1003	
Goodness-of-fit on F^2	1,096	
Final R indices [I>2sigma(I)]	R1 = 0,0478, wR2 = 0,1288	
R indices (all data)	R1 = 0,0583, wR2 = 0,1437	
Largest diff. Peak and hole	3,062 and -2,900 e.Å^{-3}	

Table S3. Hydrogen bonds for **Ir3** [Å and °]

D-H...A	d(D-H)	d(H...A)	d(D...A)	<(DHA)
C(15)-H(15)...F(83^a)#1	0.95	2.55	3.260(11)	131.8
C(16)-H(16)...O(81^a)#2	0.95	2.43	3.253(13)	145.2
C(16)-H(16)...O(82^a)#1	0.95	2.46	3.154(12)	129.8
C(20)-H(20)...N(1)	0.95	2.62	3.333(7)	132.3
C(62)-H(62B)...S(56)	0.99	2.74	3.459(7)	130.2

Symmetry transformations used to generate equivalent atoms:

#1 -x+1,-y+1,-z   #2 x+1,y,z

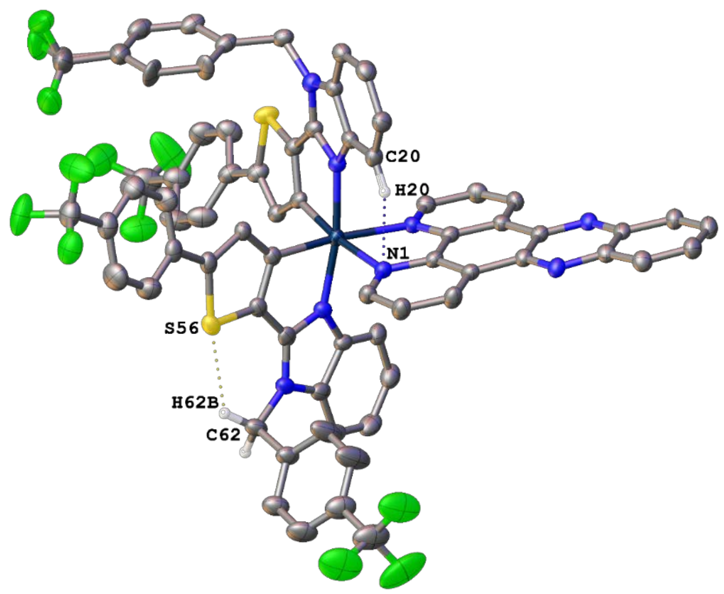

Figure S37. Hydrogen bonds in the structure of **Ir3**. Ellipsoids have been represented at 50 % probability.

Table S4. Geometrical parameters of the π···π stacking interactions for **Ir3.**

	centroid-centroid distance	Offset stacking	θ
Plane 1 –plane 2#2_675 (1-X,2-Y,-Z)	3.772(4)	4.2(3)	1.668
Plane 3-plane 4#2_666 (1-X,1-Y,1-Z)	3.878(5)	9.3(3)	1.965
Plane 4- plane 4#2_566 (-X,1-Y,1-Z)	3.853(5)	1.062	0.0(4)
Plane 2- plane 5#2_675 (1-X,2-Y,-Z)	3.862(4)	1.750	3.1(3)

Plane 1 (C4 C5 C12 C13 C17 C18), Plane 2 (C6 C7 C8 C9 C10 C11),

Plane 3 (C45 C50 C49 C48 C47 C46) and Plane 4 (C56 C61 C60 C59 C58 C57)

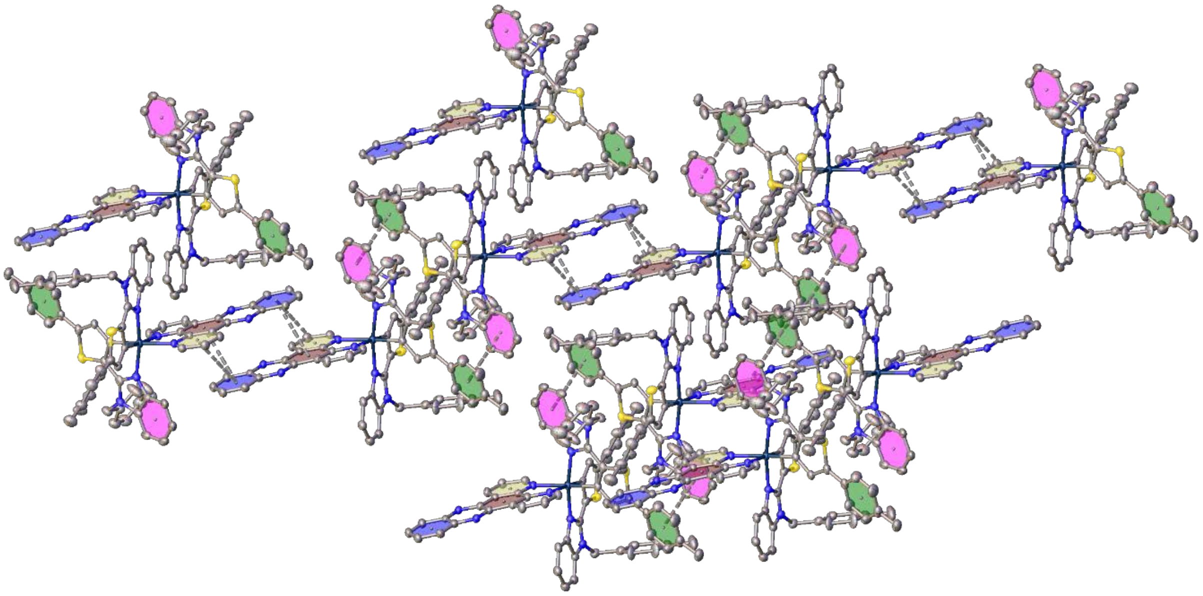

Figure S38. Intermolecular π-π interactions involving the dppz rings of **Ir3**.

Table S5. Geometrical parameters of the CH···π interactions for **Ir3**.

	C-H···centroid ring		C-H···centroid ring angle
	H···Centroid	C···Centroid	
C_1-H_1···Plane 6	2.73	3.520(7)	141°
C_{20}-H_{20}···Plane 7	2.63	3.535(7)	160°
C_{23}-H_{23}···Plane 5	2.58	3.448(7)	152°

Plane 5 (N2 C17 C13 C14 C15 C16), plane 6 (S56, C52, C53, C54, C55) and plane 7 (N1, C1, C2, C3, C4, C18)

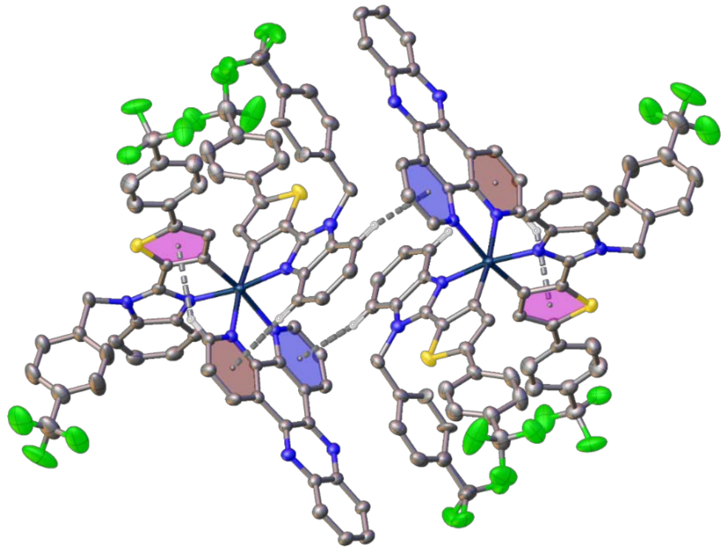

Figure S39. Intermolecular CH-π interactions involving the dppz rings of **Ir3**.

4. Photophysical properties

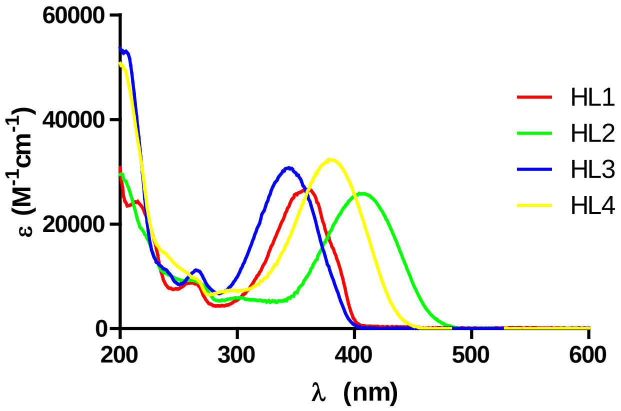

Figure S40. UV/Vis spectra of new pro-ligands **HL1-HL4**.

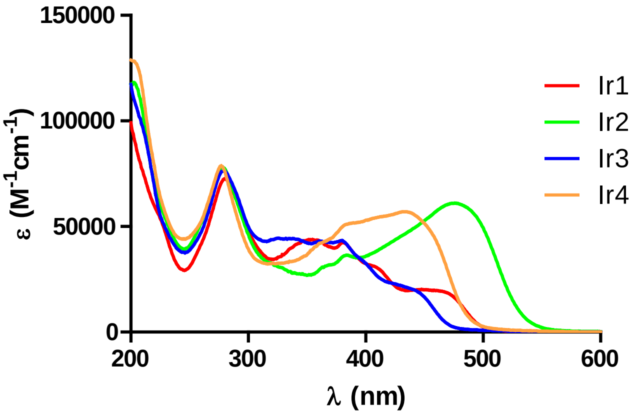

Figure S41. UV/Vis spectra of iridium compounds **Ir1-Ir4** (10 μM) in acetonitrile.

Table S6. Absorption wavelengths (λ_{abs}) and molar extinction coefficient (ε) of complexes in areated acetonitrile and water (1% DMSO)

Complex	Solvent	$\boldsymbol{\lambda}$ (nm) ($\boldsymbol{\varepsilon}$, $M^{-1}cm^{-1}$)
**Ir1**	CH_3CN	280 (72500) 355 (43850) 381 (42340)
	H_2O	284 (57390) 347 (37080) 384 (33430)
**Ir2**	CH_3CN	278 (78050) 384 (36390) 476 (61010)
	H_2O	283 (48630) 389 (22780) 478 (32230)
**Ir3**	CH_3CN	279 (76330) 379 (43330)
	H_2O	284 (59840) 382 (35810)
**Ir4**	CH_3CN	277 (78680) 434 (56980)
	H_2O	281 (49760) 395 (34040) 433 (36390)

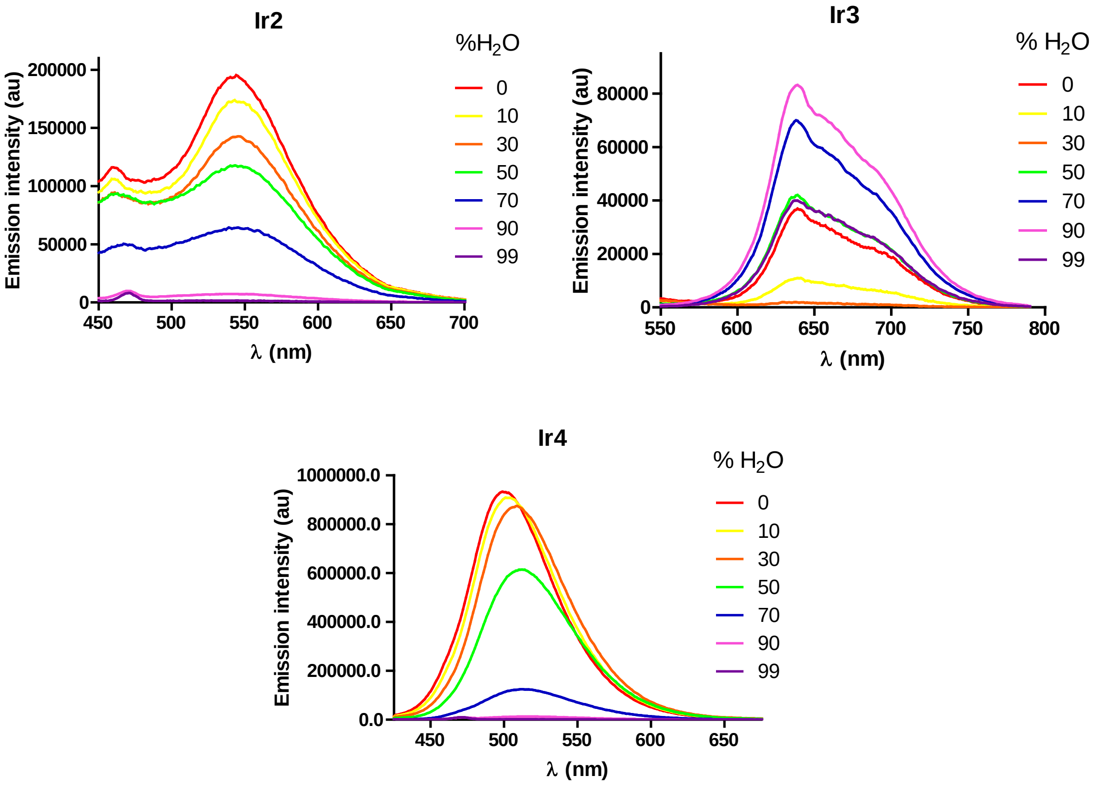

Figure S42. Emission spectra of **Ir2-Ir4** (10 μM) at different water percentage. The excitation wavelength was set to 405 nm.

5. Stability studies

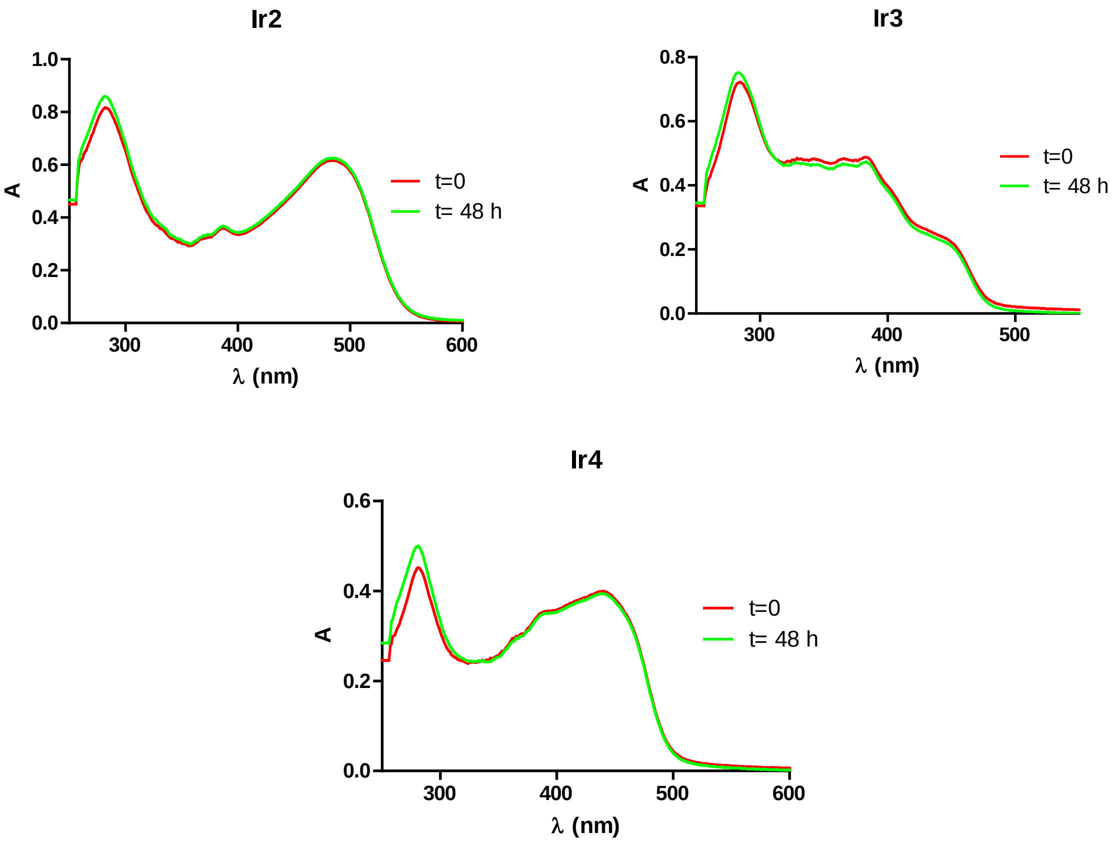

Figure S43. UV-Visible absorption spectra of complexes **Ir2-Ir4** (10 μM) in DMSO at t=0 and after 48 h.

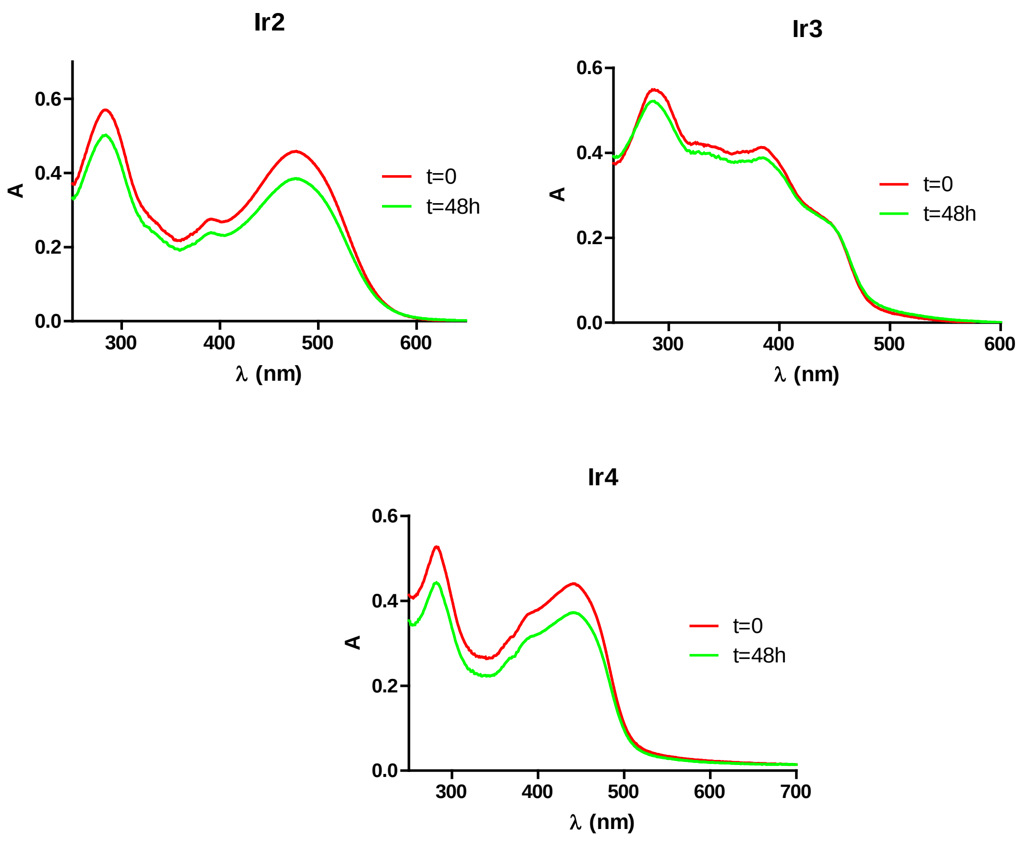

Figure S44. UV-Visible absorption spectra of complexes **Ir2-Ir4** (10 μM) in RPMI (5% DMSO) at t=0 and after incubation at 37ºC for 48 h.

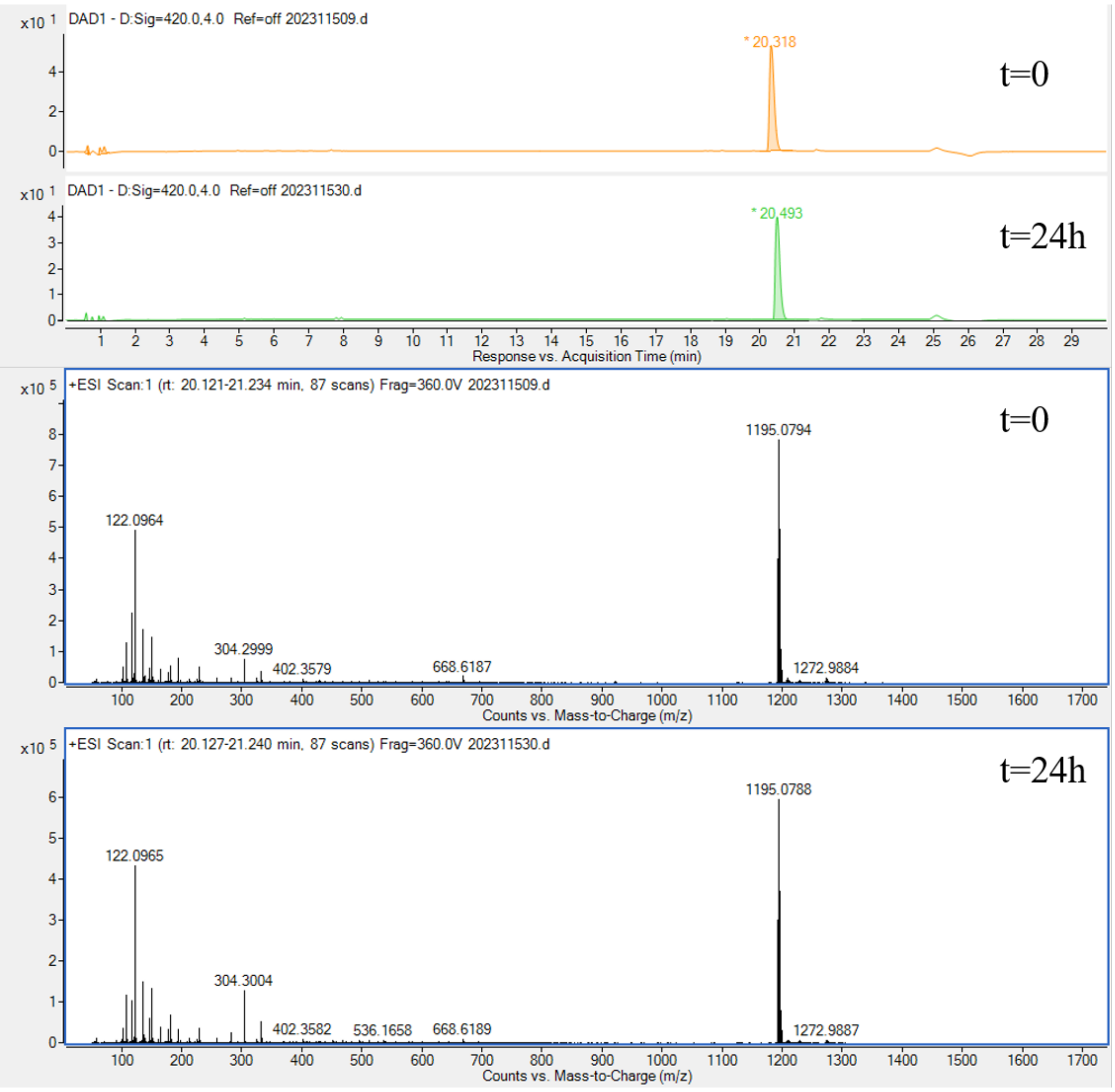

Figure S45. HPLC-MS of **Ir1** (10 μM) in RPMI (1% DMSO) at t= 0 and after 24 h at 37 ºC.

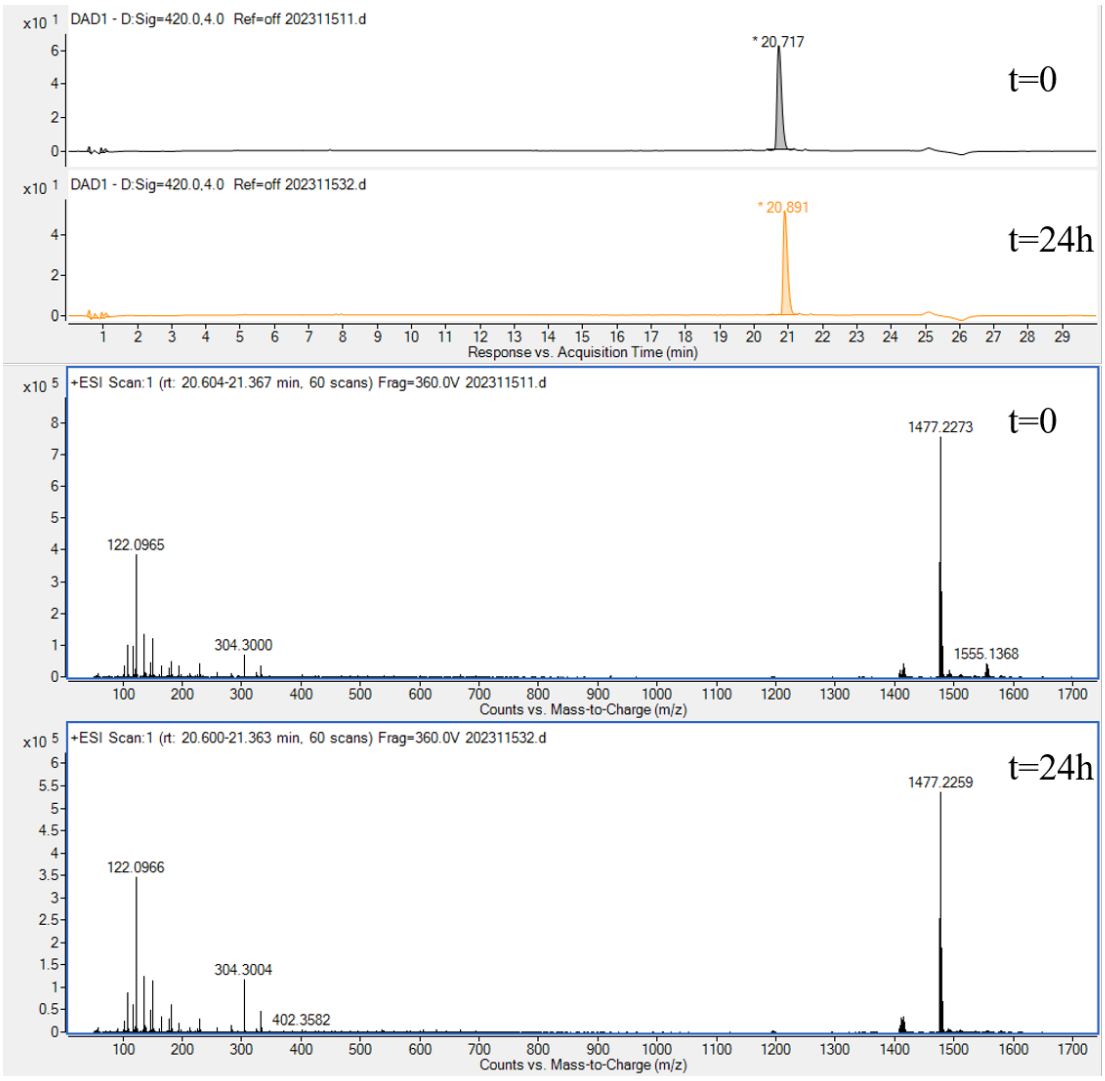

Figure S46. HPLC-MS of **Ir3** (10 μM) in RPMI (1% DMSO) at t=0 and after 24 hours at 37 ºC.

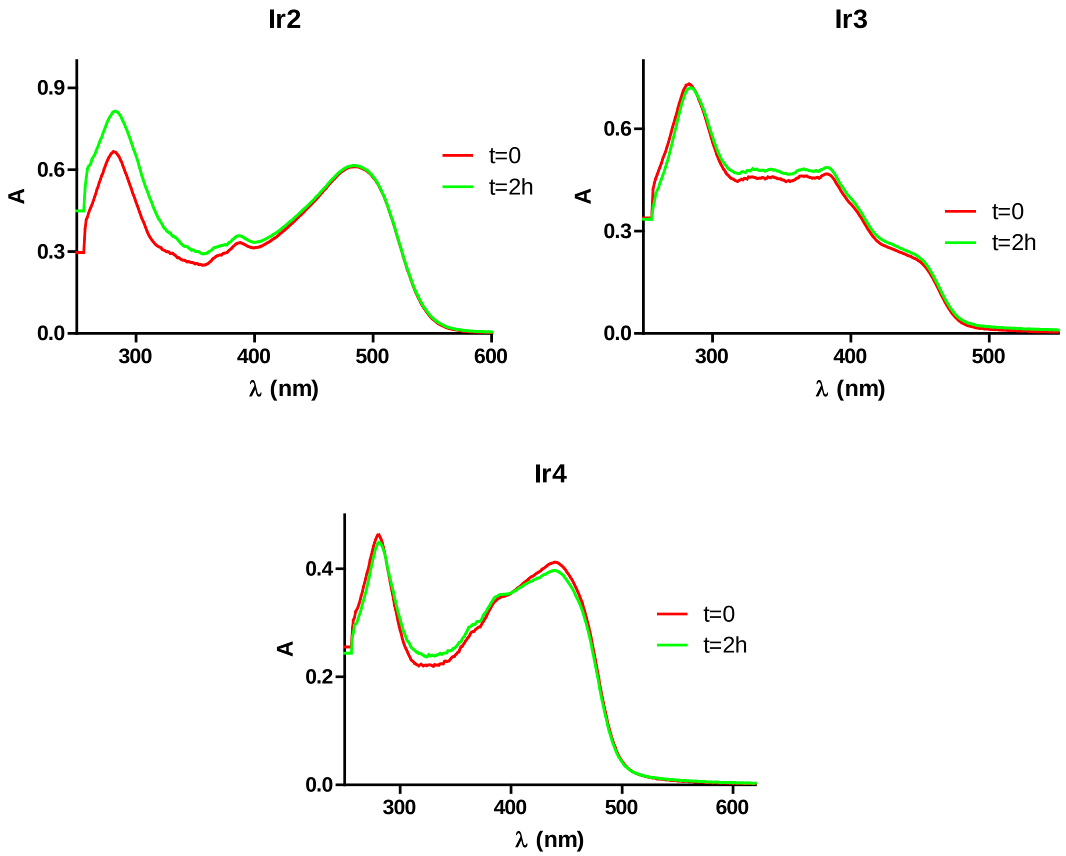

Figure S47. UV-Visible absorption spectra of complexes **Ir2-Ir4** in DMSO (10 μM) of concentration at t=0 and after blue light irradiation (4 mW/cm^2) for 2 h.

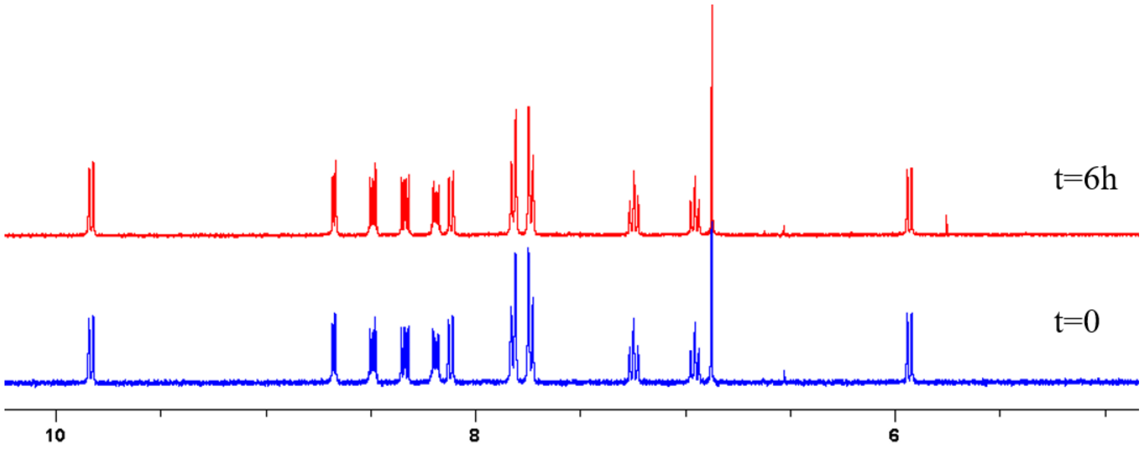

Figure S48. ^{1}H NMR spectra of **Ir1** in DMSO at 25ºC after irradiation with blue light (λ = 465 nm, 4mW/cm^2) at t=0 and t=6 h.

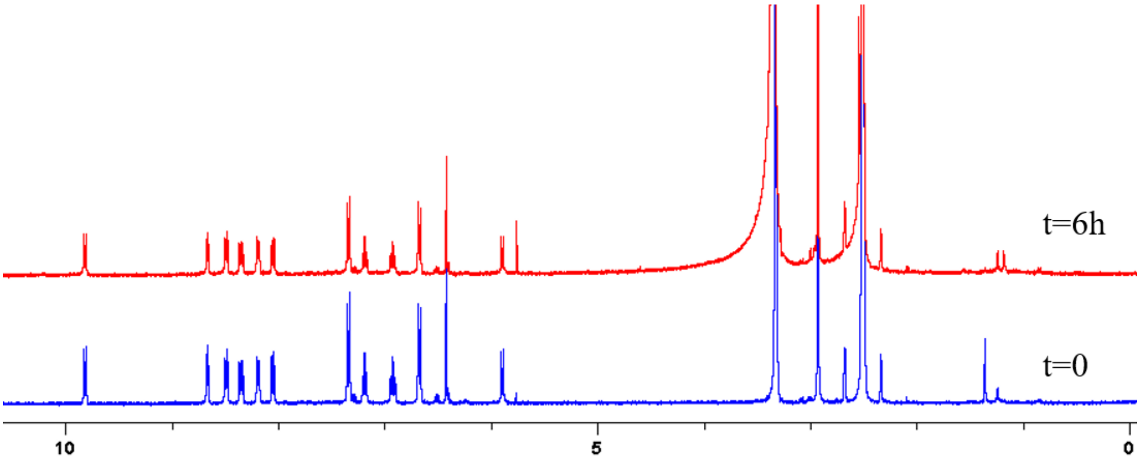

Figure S49. ^{1}H NMR spectra of **Ir2** in DMSO at 25ºC after irradiation with blue light (λ=465 nm, 4mW/cm^2) at t=0 and t=6 h.

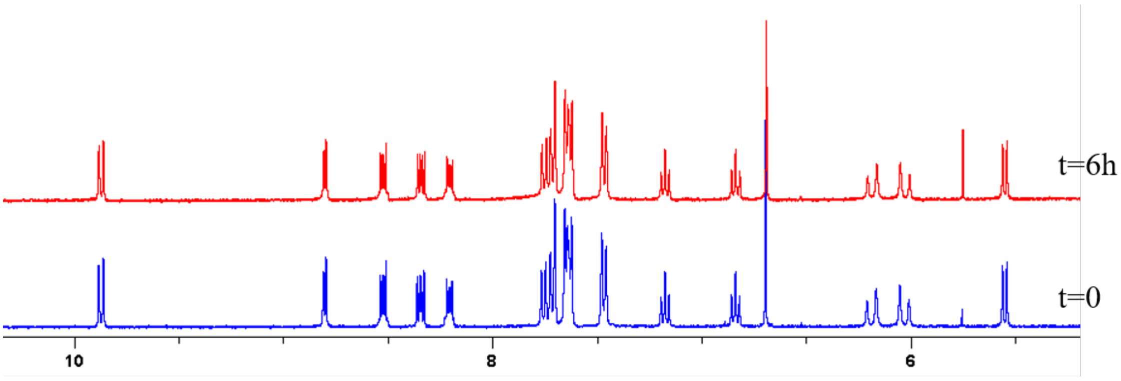

Figure S50. ^{1}H NMR spectra of **Ir3** in DMSO at 25ºC after irradiation with blue light (λ=465 nm, 4mW/cm^2) at t=0 and t=6 h.

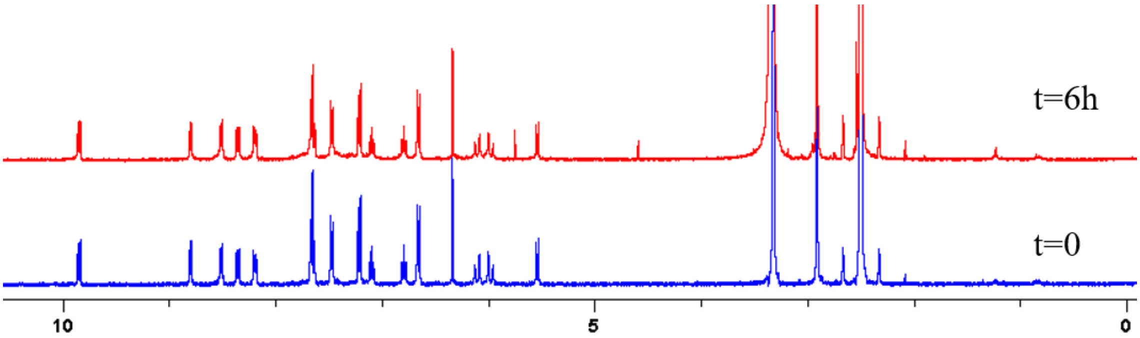

Figure S51. ^{1}H NMR spectra of **Ir4** in DMSO at 25ºC after irradiation with blue light (λ=465 nm, 4mW/cm^2) at t=0 and t=6 h.

6. Photo-oxidation of NADH and evaluation for $^{1}O_2$ and/or •OH generation in cell free media.

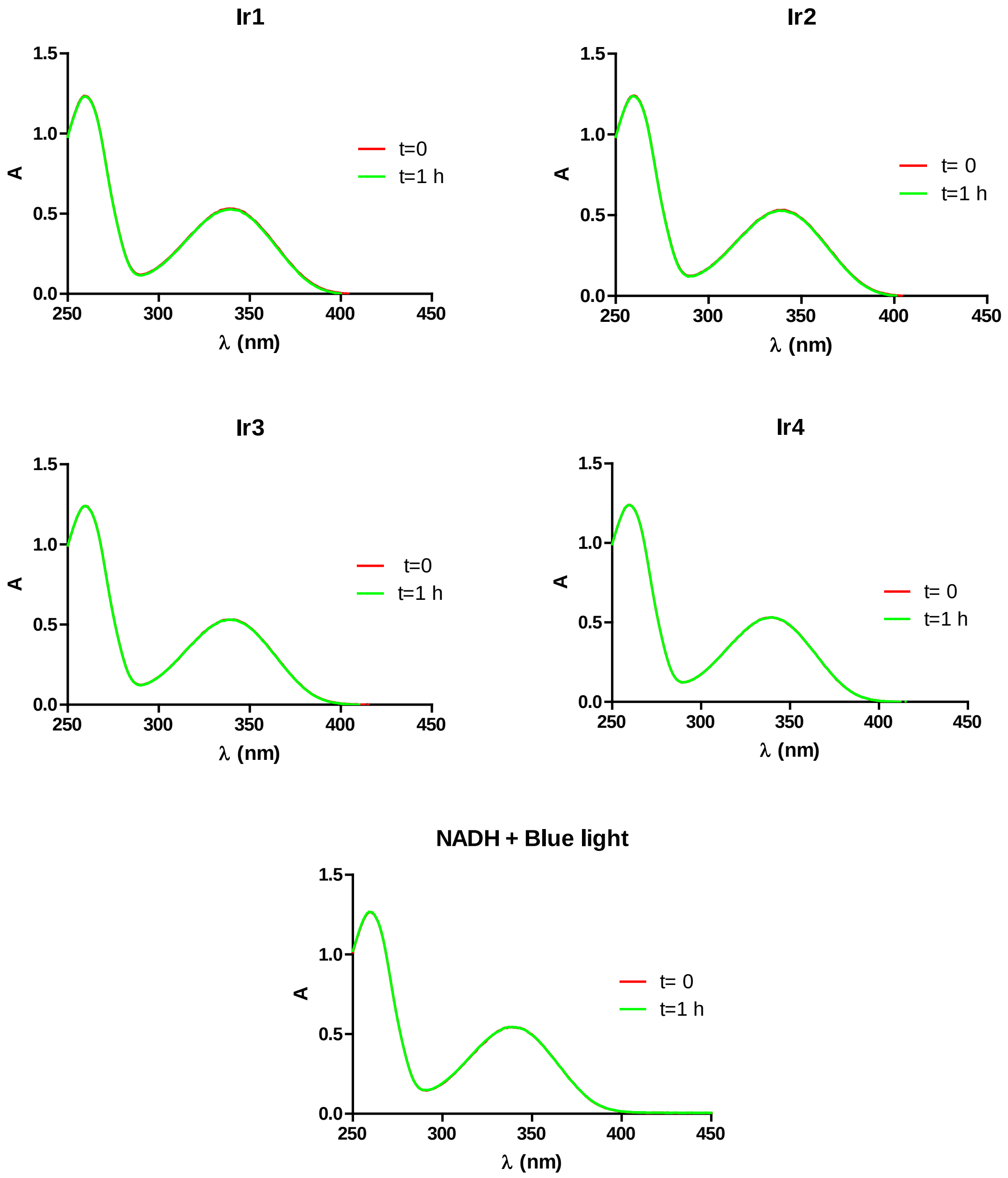

Figure S52. NADH absorption spectra (100 μM) in the presence of complexes **Ir1-Ir4** (1 μM) in PBS (5% DMF) in dark conditions and without using any complex after light irradiation (465 nm, 4.2 mW/cm^2).

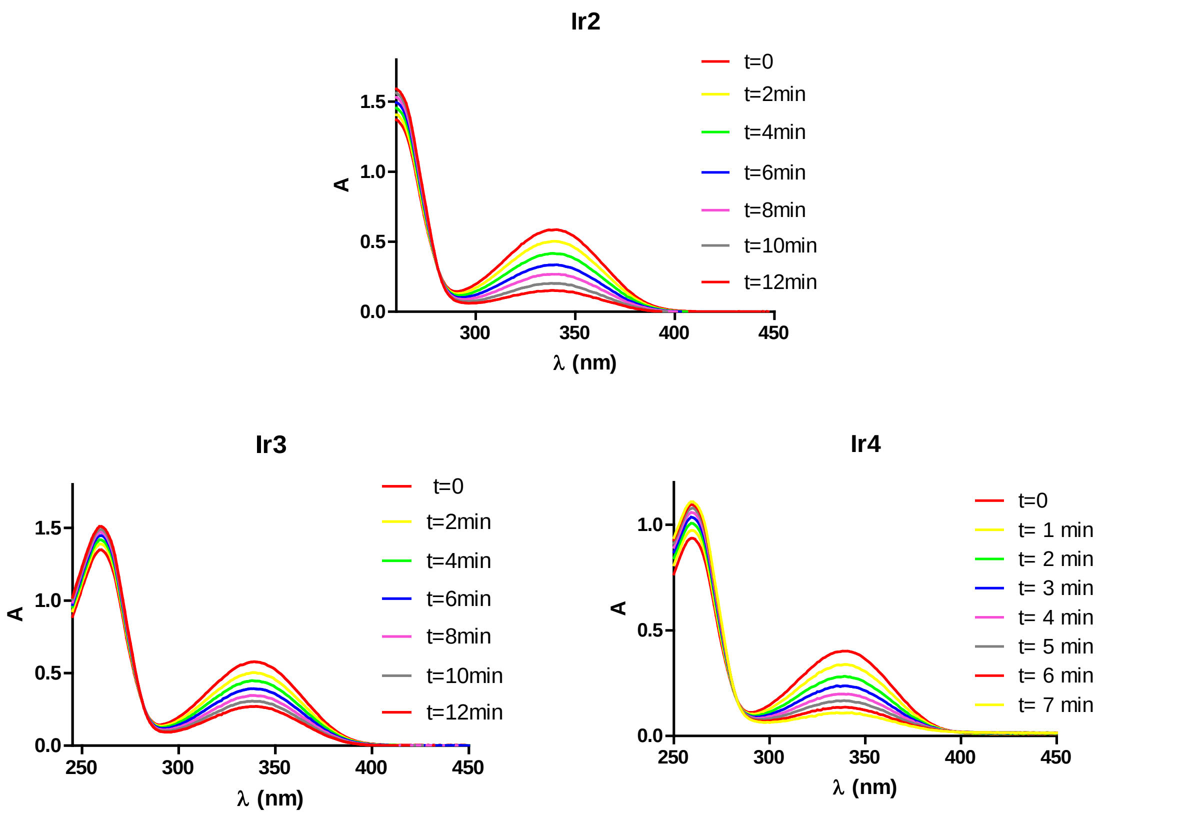

Figure S53. Decreasing of NADH absorption spectra (100 μM) in the presence of complexes **Ir2-Ir4** (1 μM) in PBS (5% DMF) under blue light irradiation (465 nm, 4.2 mW/cm^2).

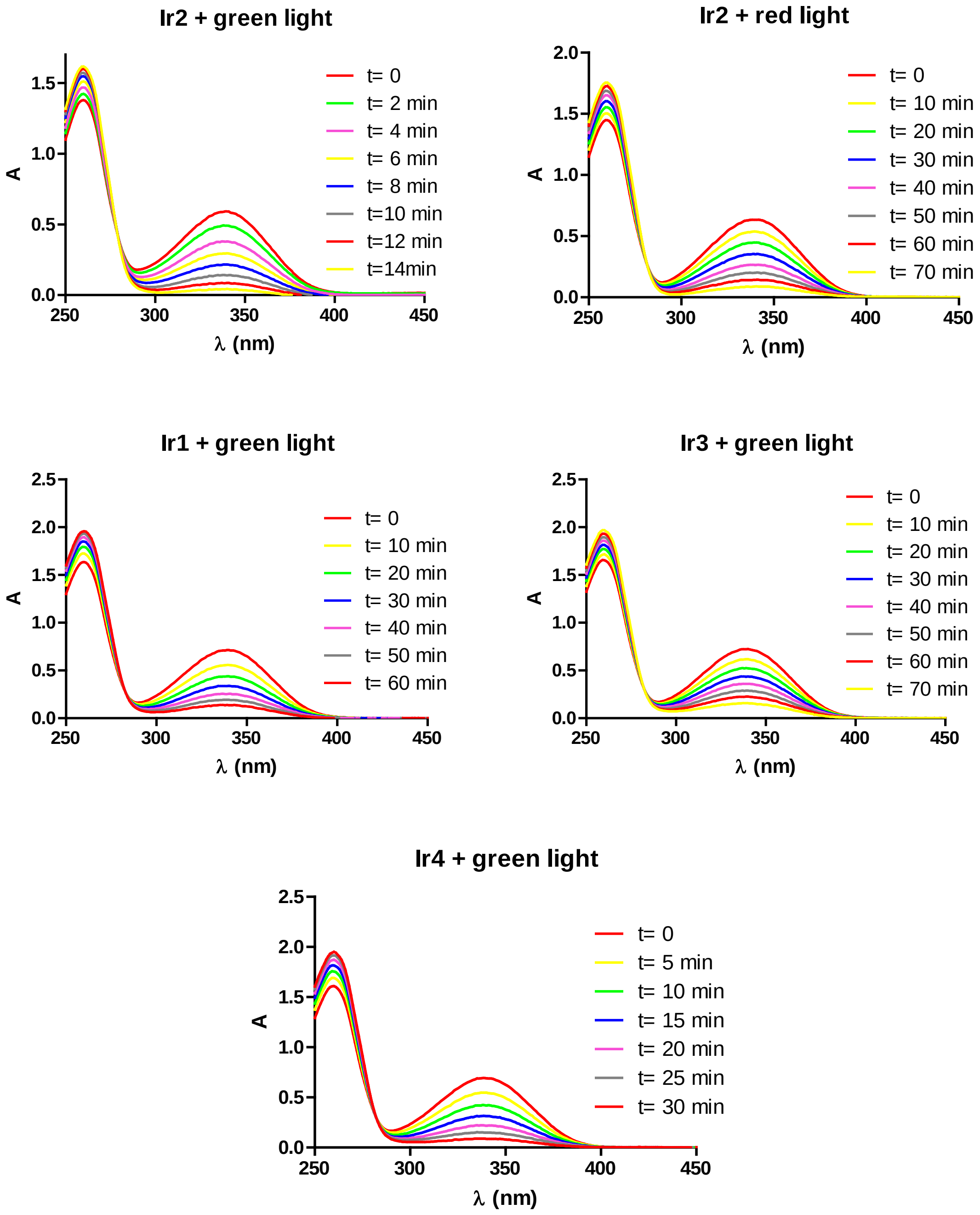

Figure S54. Decreasing of NADH absorption spectra (100 μM) in the presence of complexes **Ir1-Ir4** (5 μM) in PBS (5% DMF) under green (520 nm, 2.0 mW/cm^2) or red-light irradiation (620 nm, 15mW/cm^2).

Table S7. TON and TOF of complexes **Ir1-Ir4**. TON was defined as the number of moles of NADH that Ir complex could oxidize to NAD^+. TOF was obtained from the concentration of oxidized NADH per unit time divided by the concentration of complexes. The concentration of complexes is 1 μM when using blue light (**B**) and 5 μM when using green light (**G**).

Compound	TON (B)	TOF (h^{-1}) (B)	TON (G)	TOF (h^{-1}) (G)
**Ir1**	66.6	307.3	18.2	18.2
**Ir2**	73.5	339.3	16.6	71.3
**Ir3**	52.4	241.9	18.2	15.6
**Ir4**	47	403.1	19.4	38.9

A)

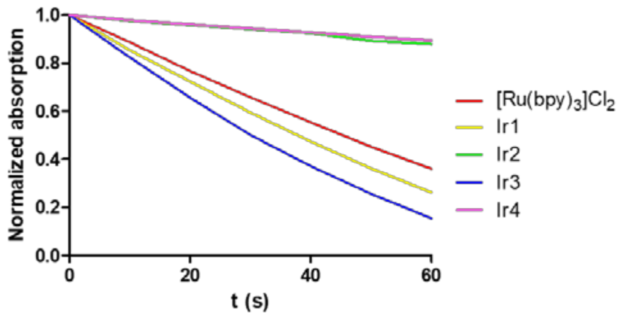

B)

Compound	ϕ_Δ
**Ir1**	63.2
**Ir2**	10
**Ir3**	68.7
**Ir4**	9.2

Figure S55. A) Absorbance decrease of DPBF (50 μM) in presence of complexes **Ir1-Ir4** and the reference $[Ru(bpy)_3]Cl_2$ (5 μM) in aerated acetonitrile when irradiating with blue light (465 nm, 0.5 mW/cm^2). B) Singlet oxygen quantum yields for complexes **Ir1-Ir4.**

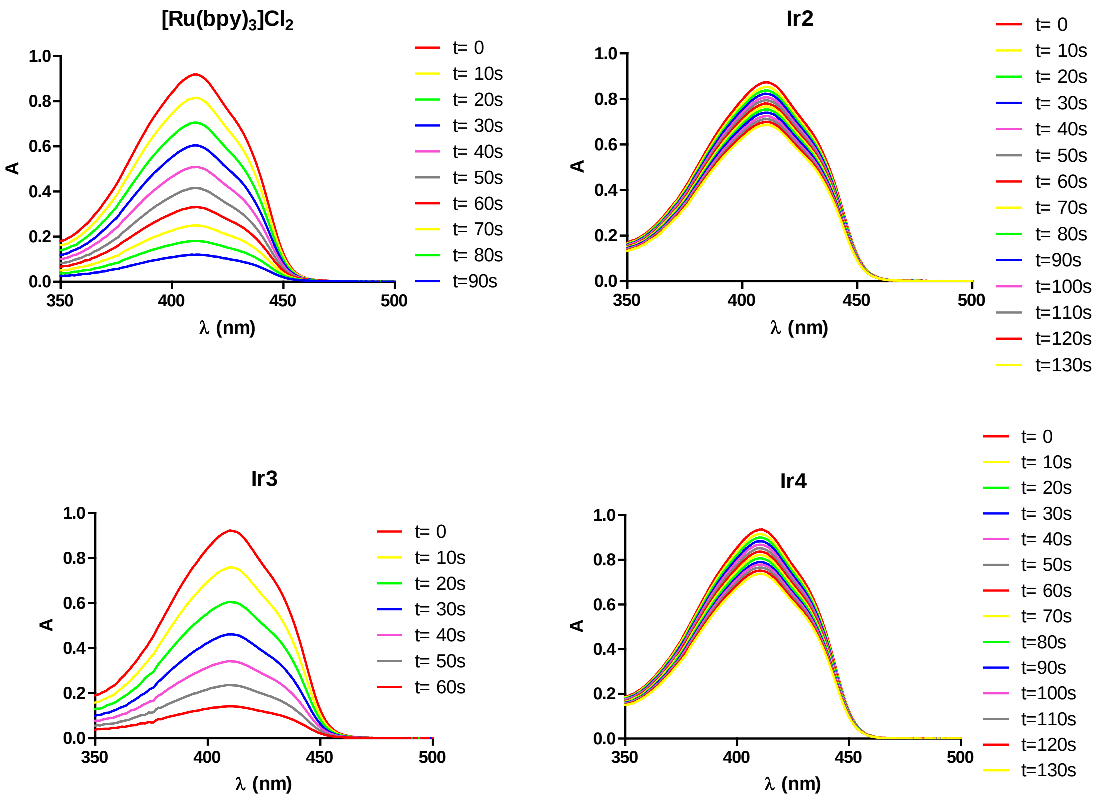

Figure S56. Absorbance decrease of DPBF (50 μM) in presence of complexes **Ir2-Ir4** and the reference $[Ru(bpy)_3]Cl_2$ (5 μM) in aerated acetonitrile when irradiating with blue light (465 nm, 0.5 mW/cm^2).

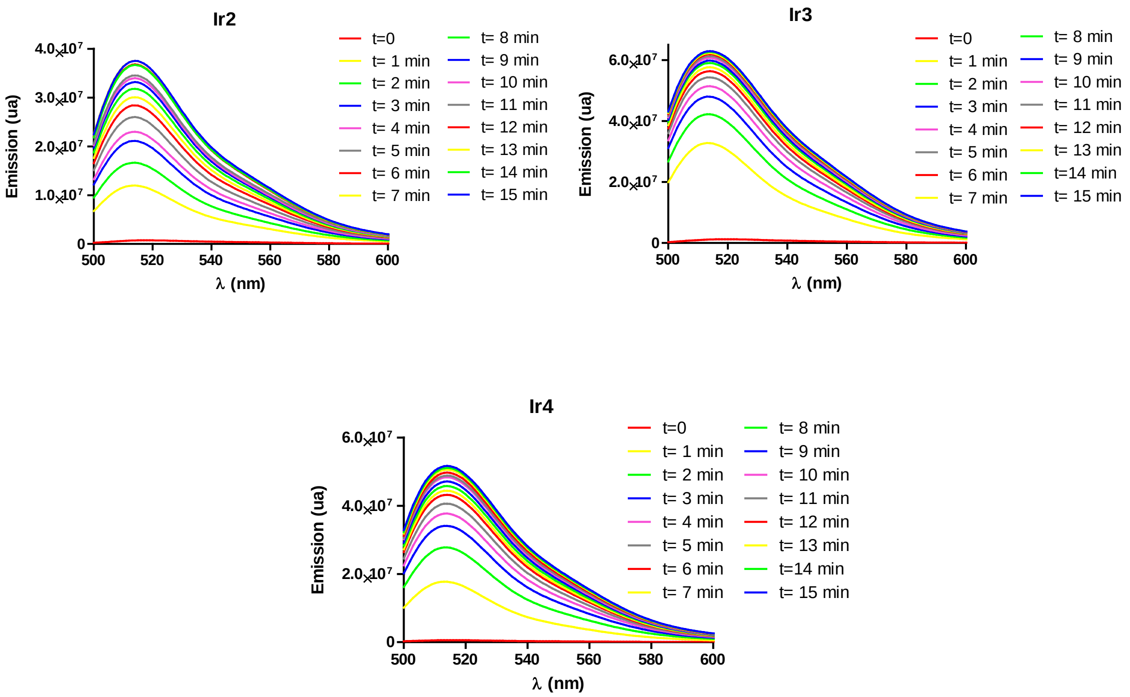

Figure S57. Increase of the fluorescence spectra emission of HPF upon photoirradiation of Ir(III) complexes Ir2-Ir4 at 465 nm (4.2 mW/cm^2) in PBS (5 % DMF). HPF fluorescence was excited at 490 nm.

7. Biological assays

Table S8. IC_{50} values (µM) obtained for HeLa cells treated with the Ir complexes and irradiated by blue light (1 h, λ_{max} = 420 nm, 58 ± 2 W m^{-2}) or sham irradiated as determined by the SRB assay[a]

	irrad	sham irrad
**Ir1**	0.27 ± 0.02	> 50
**Ir2**	0.6 ± 0.1	> 50
**Ir3**	1.1 ± 0.1	> 50
**Ir4**	4 ± 1	> 50

[a]The data are expressed as mean values ± SD, n ≥ 6.

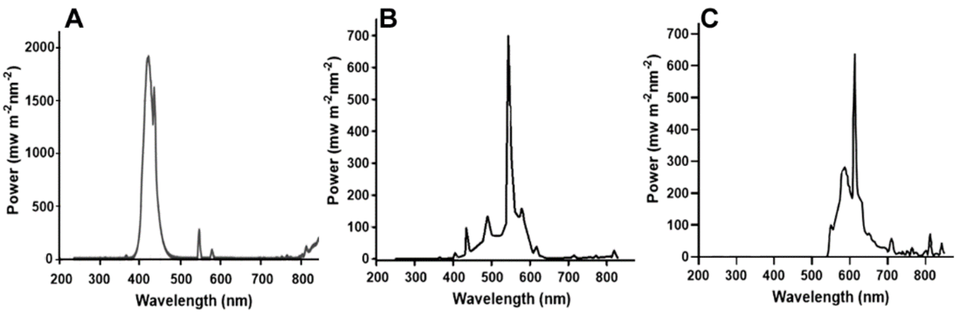

Figure S58. Spectral characteristics of blue (A), green (B), and red (C) light used in the biological experiments.

Table S9. Accumulation of Ir from **Ir1** in Hela cells. Cells were pretreated with indicated endocytosis inhibitor for 1 h and then treated with **Ir1** (3 μM, 2 h, dark)

	ng Ir/10^6 cells[a]
**Ir1**	127 ± 9
**Ir1**+ chloroquine	64 ± 13
**Ir1**+ methyl-β-cyclodextrin	67 ± 5
4 °C	13 ± 2

[a]The data are expressed as mean values ± SD, n=3.

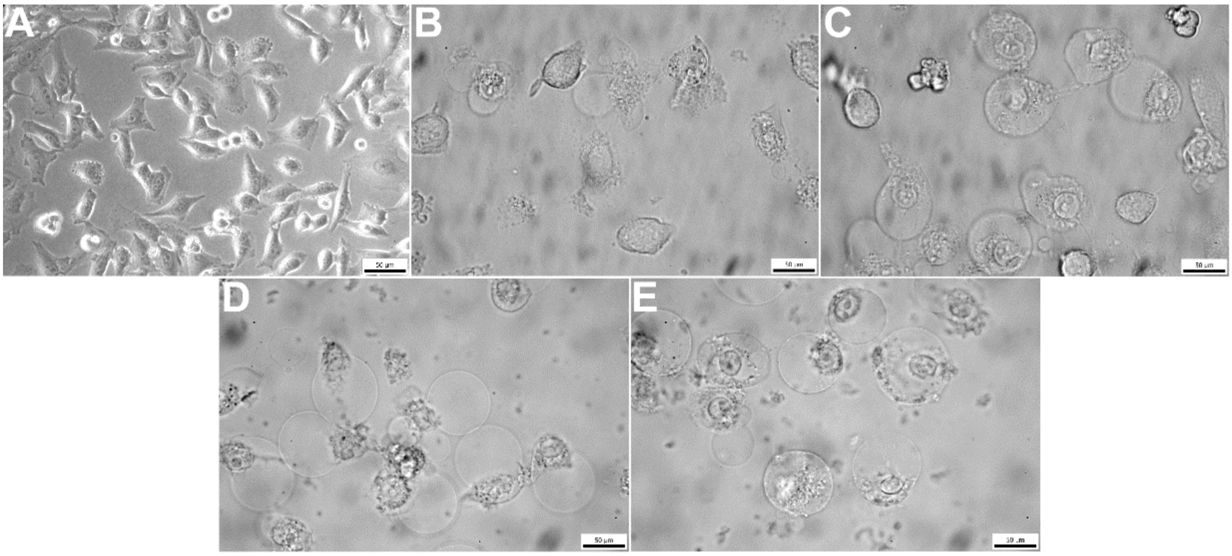

Figure S59. Morphology of the HeLa cells treated with **Ir1** (0.3 µM, panel B), **Ir2** (0.7 µM, panel C), **Ir3** (1.2 µM, panel D), **Ir4** (3.7 µM, panel E) or untreated (panel A) for 1 h, irradiated, and subsequently incubated in compound free media for 2 h in dark. Scale bars represent 50 µm.

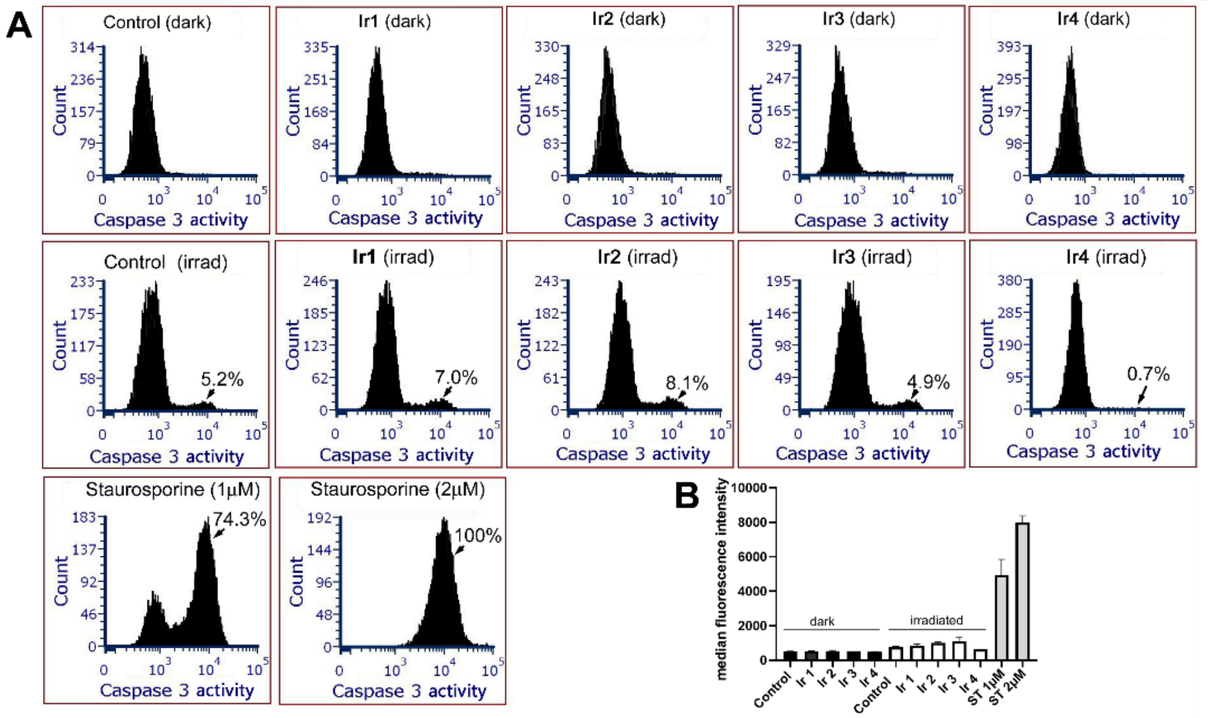

Figure S60. Activation of caspase 3 detected by CellEvent®Caspase3/7 Green Detection Reagent. HeLa cells were treated with **Ir1-4** at their equitoxic concentrations (0.36, 0.8, 1.3, and 4 µM for **Ir1**, **Ir2**, **Ir3**, and **Ir4**, respectively) for 1 h in the dark. Cells were kept in the dark (top panels) or irradiated with blue light (middle panels). Control cells were treated with a DMSO carrier. After 2 h of recovery in compound-free media, cell samples were stained with the CellEvent®Caspase 3/7 Green Detection Reagent, and fluorescence was analyzed by flow-cytometry; 30 000 cells were analyzed in each sample. A) Representative histograms. B) Quantitative evaluation. In irradiated samples, a negligible amount of apoptotic cells are detected (indicated by arrows); however, the apoptotic population is not significantly different from that in control, untreated irradiated cells. In contrast, staurosporine treatment significantly increases the number of apoptotic, caspase-3/7 positive cells (bottom panels).

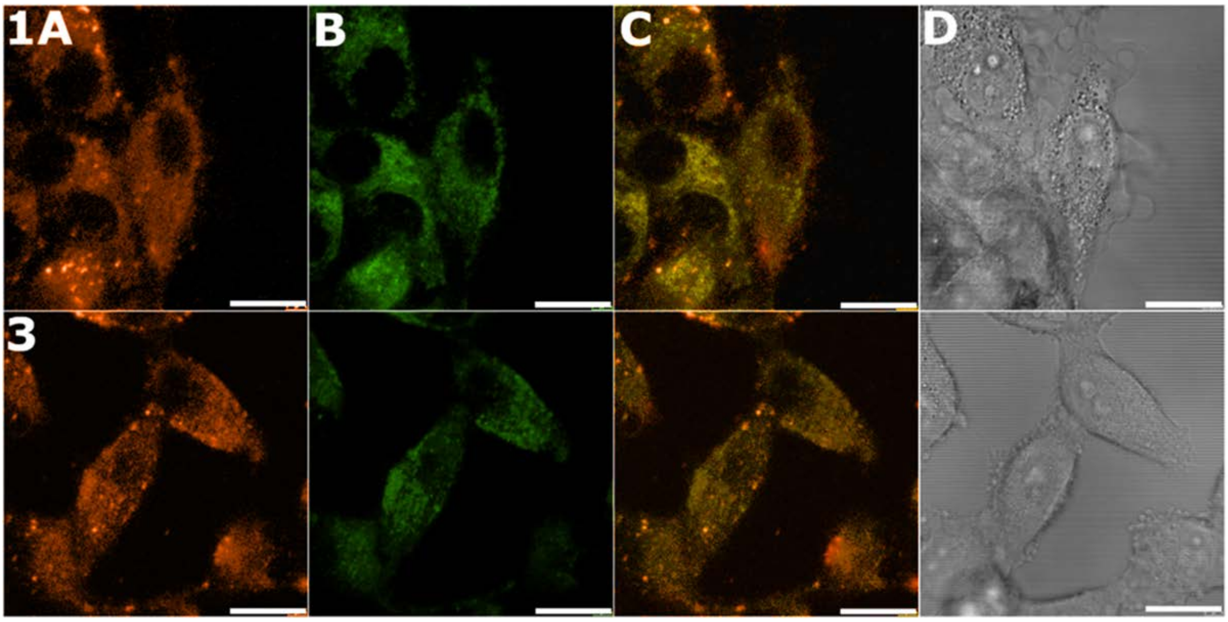

Figure S61. Colocalization of **Ir1** and **Ir3** with lysosomes in Hela cells determined by confocal microscopy. Cells were treated with 2 µM of tested compounds and incubated for 3 h before staining with LysoTracker® Green. Channels: A) fluorescence signal coming from tested iridium compounds, B) LysoTracker® fluorescence, C) overlay of the fluorescence coming from tested compounds and LysoTracker®, D) bright field channel. The scale bar indicates 20 µm.

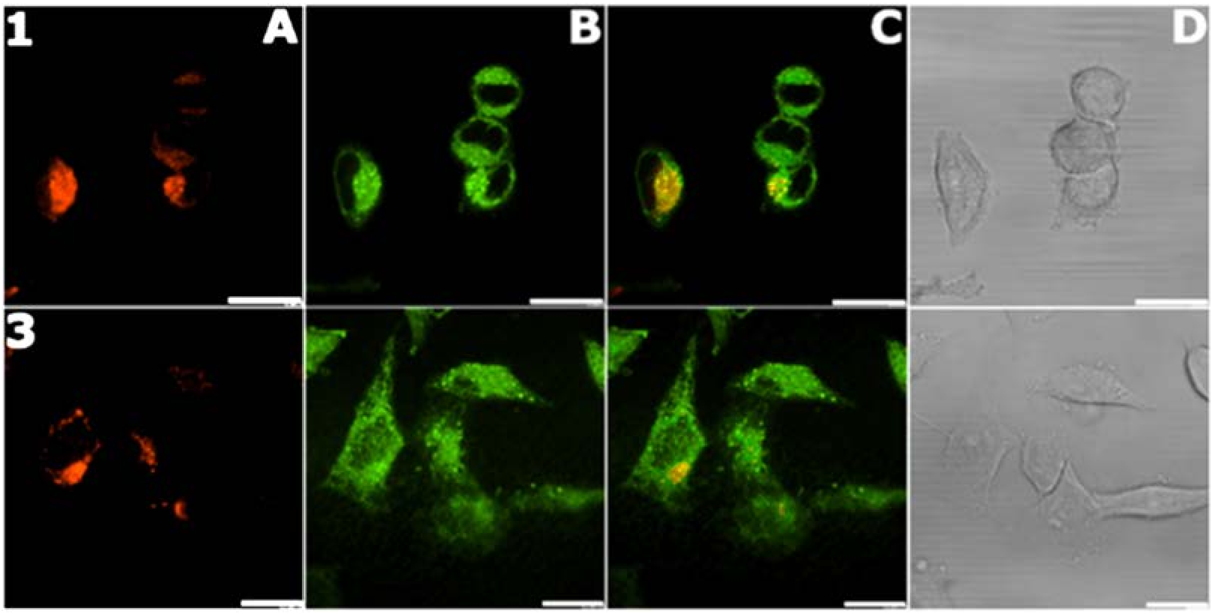

Figure S62. Colocalization of **Ir1** and **Ir3** with mitochondria in Hela cells determined by confocal microscopy. Cells were treated with 2 µM of tested compounds and incubated for 3 h before staining with MitoTracker®. Channels: A) fluorescence signal coming from tested iridium compounds, B) MitoTracker® fluorescence, C) overlay of the fluorescence coming from tested compounds and MitoTracker®, D) bright field channel. For better clarity, the MitoTracker® signal is shown in false colors (green). Details on scanning and image acquisition are provided in the Material and methods section. The scale bar indicates 20 µm.

Table S10: Pearson colocalization coefficients[a] determined for **Ir1** and **Ir3** in HeLa cells co-stained with LysoTracker® or MitoTracker® probes. Pearson colocalization coefficients were determined in ImageJ software by using Costes threshold algorithm with subsequent direct colocalization analysis.

	**Pearson Lyso**	**Pearson Mito**
**Ir1**	0.76 ± 0.07	0.50 ± 0.06
**Ir3**	0.79 ± 0.11	0.42 ± 0.04

[a]Colocalization coefficients calculated from three independent experiments, at least ten images per sample for each experiment.

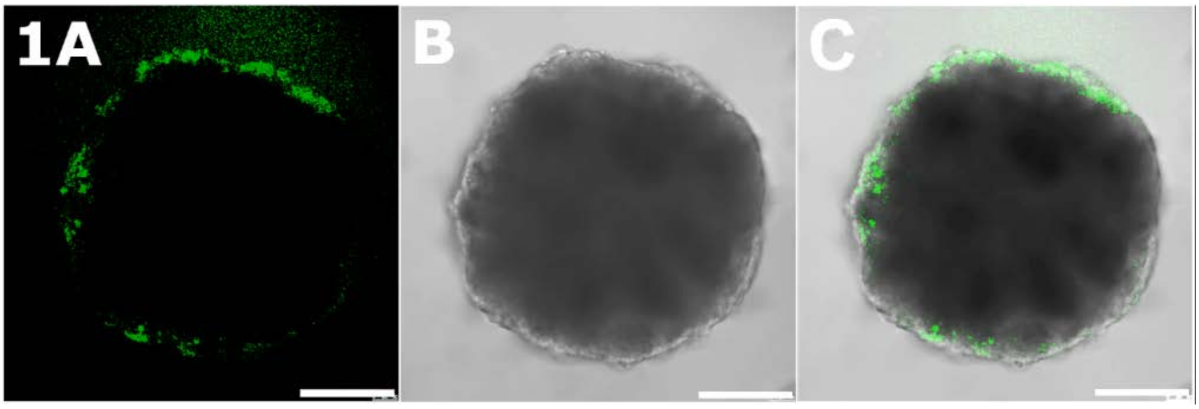

Figure S63. Accumulation of **Ir1** in HeLa spheroid. HeLa cells were treated with 2 µM of the tested compound and incubated for 5 h before analysis on a confocal microscope. Channels: A) Fluorescence from the **Ir1** complex, B) bright-field channel, C) overlay of the fluorescence and bright field channel. The scale bars represent 200 µm.
